# Application of Deep Eutectic Solvents in the Synthesis of Substituted 2-Mercaptoquinazolin-4(3*H*)-Ones: A Comparison of Selected Green Chemistry Methods

**DOI:** 10.3390/molecules27020558

**Published:** 2022-01-16

**Authors:** Mario Komar, Tatjana Gazivoda Kraljević, Igor Jerković, Maja Molnar

**Affiliations:** 1 Faculty of Food Technology Osijek, Josip Juraj Strossmayer University of Osijek, Franje Kuhača 18, HR-31000 Osijek, Croatia; mkomar@ptfos.hr; 2 Department of Organic Chemistry, Faculty of Chemical Engineering and Technology, University of Zagreb, Marulićev Trg 19, HR-10000 Zagreb, Croatia; tgazivod@fkit.hr; 3 Faculty of Chemistry and Technology, University of Split, Ruđera Boškovića 35, HR-21000 Split, Croatia

**Keywords:** deep eutectic solvents, 2-mercaptoquinazolin-4(3*H*)-one, microwave-assisted synthesis, ultrasound-assisted synthesis, green chemistry

## Abstract

In this study, deep eutectic solvents (DESs) were used as green and eco-friendly media for the synthesis of substituted 2-mercaptoquinazolin-4(3*H*)-ones from different anthranilic acids and aliphatic or aromatic isothiocyanates. A model reaction on anthranilic acid and phenyl isothiocyanate was performed in 20 choline chloride-based DESs at 80 °C to find the best solvent. Based on the product yield, choline chloride:urea (1:2) DES was found to be the most effective, while DESs acted both as solvents and catalysts. Desired compounds were prepared with moderate to good yields using stirring, microwave-assisted, and ultrasound-assisted synthesis. Significantly, higher yields were obtained with mixing and ultrasonication (16–76%), while microwave-induced synthesis showed lower effectiveness (13–49%). The specific contribution of this research is the use of DESs in combination with the above-mentioned green techniques for the synthesis of a wide range of derivatives. The structures of the synthesized compounds were confirmed by ^1^H and ^13^C NMR spectroscopy.

## 1. Introduction

**Figure 1:**
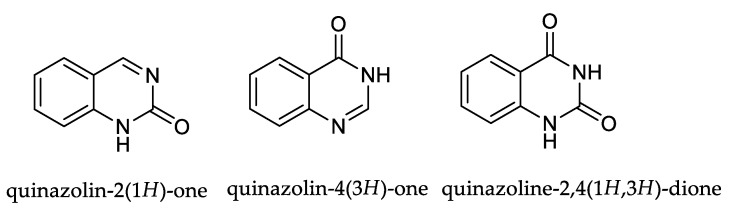
Structure of quinazolinone and quinazolinedione cores [1].

Quinazolinones, as oxygen- and nitrogen-containing fused heterocyclic compounds, are an important scaffold in pharmaceutical and medicinal chemistry showing significant biological activities. They exist in the form of two isomers. The keto functionality may be present on the second, fourth, or both carbon atoms of the quinazoline nucleus (Figure 1) [1].

**Figure 2:**
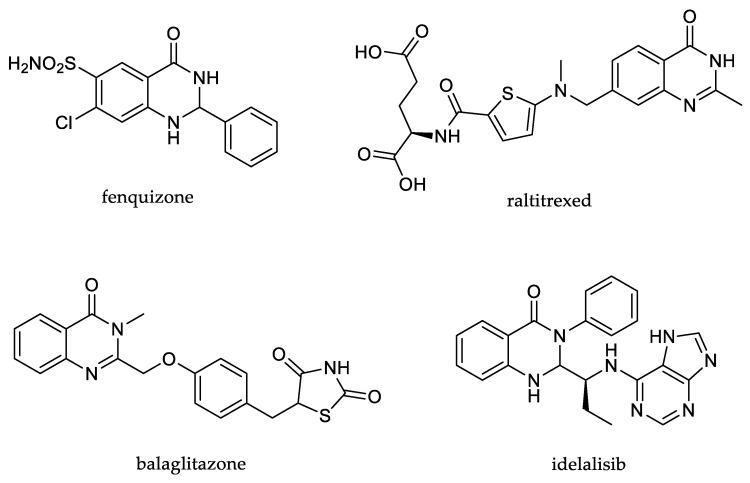
Quinazolinone-based registered drugs [12].

Exceptional biological activities of quinazolinone derivatives have been demonstrated in previous studies investigating their antibacterial [2], antitumor [3], anticonvulsant [4] and anti-inflammatory [5] and enzyme inhibitory activity [6,7]. These compounds have also been found to act via various targets such as tubulin [8], dihydrofolate reductase [9], histone deacetylase [10], COX-1/COX-2 and 15-LOX [11]. Numerous registered drugs contain quinazolinone core in their structure, such as fenquizone, which is used as a diuretic, raltitrexed, used for selective treatment of different cancers, while balaglitazone is effective anti-diabetic drug and idelalisib as an antineoplastic agent (Figure 2) [12]. An extraordinary biological activity makes them an interesting target for medicinal investigations; therefore, their preparation has been intensified lately [13]. 2-Mercaptoquinazolinones have been a fruitful source in pharmaceutical and medicinal chemistry considering their capability of interacting in hydrogen bonding and π-π stacking with biological targets [14].

The synthesis of quinazolinone derivatives has been the focus of numerous studies for decades, and is therefore well described in the literature. There are many different methods for preparing these extensively studied heterocyclic compounds. Among them, the main synthetic routes use anthranilic acid [15,16,17,18,19], anthranilate [20], isatoic anhydride [21,22,23], benzoxazinone [24,25,26,27] and 2-iodoaniline [28] as convenient precursors. For preparation of 2-mercaptoquinazolinone derivatives, anthranilic acid is the most common starting material. Moussa et al. [11] performed a condensation by heating anthranilic acid and four aromatic isothiocyanates in ethanol for 8 h. The same procedure was performed between 5-iodoanthranilic acid and *p*-chlorophenyl isothiocyanate under reflux for 4 h with 75% yield [29]. Numerous reports have demonstrated the use of triethylamine as a base catalyst in ethanol for condensation of anthranilic acid and appropriate isothiocyanate for 2–4 h [30,31,32].

Lately, many endeavors have been directed towards much safer, economically acceptable, and environmentally friendly protocols for the preparation of quinazolinones [27,33,34]. Many green procedures use safer solvents and tend to minimize the use of toxic catalysts. Therefore, a novel class of green solvents, deep eutectic solvents (DESs), has found application in green synthetic processes, often serving as both solvents and catalysts. First reported in 2003 [35], DESs are defined as a mixture of two or more components with lower melting point than each individually. Today, DESs are classified into five types based on their general formula [36]. Commonly used, Type III DESs [36], are often formed of hydrogen bond acceptors (HBA), such as quaternary ammonium salts, and hydrogen bond donors (HBD) in different molar ratios. The HBDs employed are often urea and its derivatives, polyols, carbohydrates, and dicarboxylic acids. Their green character is manifested in easy preparation, chemical stability, non-volatility, biodegradability, and non-flammability. These are the reasons they have been increasingly used in the synthesis of heterocycles, both as catalysts and solvents [37,38,39]. Hence, a suitable combination of starting components can improve the desired physicochemical properties of DESs for many processes over conventional ones. As green solvents, DESs can be combined with other green chemistry techniques to minimize the adverse environmental effects [40].

In the last two decades, microwave heating has been intensively used in different chemical transformations. Irradiation of polar or less polar molecules due to the absorption of microwaves converted into the heat causes reduced reaction time as well as energy consumption [41]. Besides microwave-assisted synthesis, another efficient green method used to accelerate the organic conversions is an ultrasound-assisted synthesis. The generated high local pressure and temperature inside the bubbles, which collapse, results in remarkable advantages such as enhanced yields, shorter reaction time and better selectivity [42].

Choline chloride-based DESs have been in focus of our investigation for some time, and we found them to be suitable for different chemical processes, both heterocyclic compounds synthesis [17,25,33,43] and extraction [44]. In this regard, the objectives of this research were focused on the investigation on finding an appropriate Type III [36], choline chloride-based deep eutectic (ChCl-based DES) solvent for the synthesis of 3-substituted 2-mercaptoquinazolin-4(3*H*)-one derivatives, as well as on the influence of some reaction parameters such as temperature and reaction time. Furthermore, the use of several suitable green chemistry methods (microwave-assisted synthesis, and ultrasound-assisted synthesis) was also investigated. The DES was recovered and recycled at least several times. Remarkably, this is the first extensive study of the application of 20 choline chloride-based DESs in the synthesis of substituted 2-mercaptoquinazolin-4(3*H*)-ones in a combination with above mentioned green synthetic methods and their comparison.

## 2. Results and Discussion

**Scheme 1:**
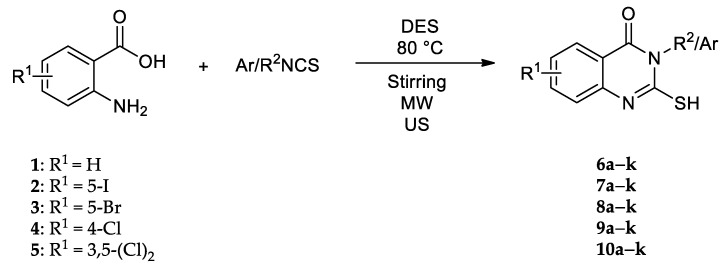
Synthesis of substituted 2-mercaptoquinazolin-4(3*H*)-ones by green methods.

Conventionally, 2-mercaptoquinazolin-4(3*H*)-ones have usually been synthesized from anthranilic acid and isothiocyanate using ethanol as a solvent and triethylamine as a base catalyst. Triethylamine is commonly employed in a stoichiometric amount. Novel green techniques in the synthesis of quinazolinones have attracted considerable attention reducing the adverse effects on the environment. Herein, we describe a synthesis of 54 substituted 2-mercaptoquinazolin-4(3*H*)-one derivatives **6a**–**10k** from anthranilic acids **1**–**5** and aliphatic and aromatic isothiocyanates (Scheme 1) using DESs by using microwave and ultrasound promoted synthesis. Structures of all synthesized compounds were confirmed by NMR spectroscopy (spectra were shown in Supplementary Material). Chemical shifts in ^1^H NMR spectra indicate that all compounds are in thiol form, where the characteristic shift for thiol proton is usually found around 12 ppm. All other characteristic chemical shifts indicate the presence of quinoline core, as well as aromatic and aliphatic substituents.

The first step of this investigation began with optimization of the reaction conditions using anthranilic acid **1** and phenyl isothiocyanate as model substrates. As our previous results [33] indicated that products could be easily synthesized in ChCl:urea (1:2) DES, we investigated the influence of reaction temperature (40 °C, 60 °C, and 80 °C) and time (30 min, 1 h and 2 h) in this DES. The consumption of the reactants was monitored with thin layer chromatography (TLC). As expected, the highest temperature of 80 °C after 1 h and 2 h provided the best product yields equally (63%), while 40 °C and 60 °C after 2 h provided 14%, and 29%, respectively. Therefore, we used the temperature of 80 °C for all reactions performed in the remaining 19 DESs (Table 1). A temperature of 80 °C was also suitable for the DESs such as ChCl:fructose (2:1), ChCl:glucose (2:1), ChCl:citric acid (1:1), ChCl:tartaric acid (1:1), which are too viscous at lower temperatures, but yet not degraded at this temperature.

The used molar ratio of the components was chosen upon literature data described before [35,37,45,46]. The product yields are shown in Table 1.

Results in Table 1 indicate that the best yield was obtained in choline chloride:urea (1:2) DES at 80 °C This yield was lower than the one obtained in our previous work [33], but this is probably due to the lower reaction temperature. As already mentioned, the temperature of 80 °C was chosen as suitable for the sugar-based DESs, since they decompose at higher temperatures. As conventional synthesis of 2-mercaptoquinazolin-4(3*H*)-ones is usually base catalyzed (often by triethylamine) it is not surprising that choline chloride:urea (1:2), a basic DES [47], gave the best yields. It can be concluded that the formation of quinazolinones in DESs is pH-dependent, with higher yields in basic DESs.

Therefore, all desired products **6a**–**10k** (Scheme 1) were synthesized in ChCl:urea (1:2) DES by simple stirring of the reaction mixture at 80 °C (Table 2).

In general, the compounds with aliphatic groups at C-3 of quinazolinone core were obtained in lower yields, probably due to their higher solubility in water during the isolation.

To explore the efficiency of current procedure, we performed the microwave and ultrasound induced synthesis of all compounds in the same solvent, as well. Microwave-induced synthesis of the model reaction was performed at 80 °C and 1800 W for 15 min, 30 min and 60 min. The desired product **6d** was obtained in traces after 15 min and 30 min, and only in 22% after 60 min. In this manner, the synthesis of all other derivatives was performed at 80 °C and 1800 W for 60 min (Table 2).

The ultrasound induced synthesis of the model reaction was performed at 50 W and 80 °C for 15 min, 30 min and 60 min affording the desired product **6d** in 14%, 15%, and 34%. Therefore, all the reactions were performed for 60 min at 50 W and 80 °C, and the results are shown in Table 2.

Besides its desirable character, the synthesis in DES is characterized by the simplicity of the postsynthetic procedures, which include the addition of water into reaction mixture followed by the product precipitation. The products are pure and only several derivatives **6c**, **6j**, **7b**, **9b**, **9c**, **9i** and **10a**–**10k** were recrystallized from ethanol. Although some other authors have obtained higher yields of some derivatives (Table 2), their synthetic procedures often included long reaction times [48,52], reflux conditions [3,29,50,51,52,53,55,57], use of different catalysts, and extensive postsynthetic procedures using different harmful organic solvents [29,50,53,54,55].

The recyclability of choline chloride:urea (1:2) DES in this investigation was examined using 5-bromoanthranilic acid **3** and *p*-tolyl isothiocyanate under optimal conditions (Table 3). After the consumption of 5-bromoanthranilic acid, the reaction mixture was cooled and diluted with water. The desired product was filtered off and washed with water. The water was removed under vacuum and DES was recovered and re-used for the same reaction. The reusability proceeded four times with no significant loss of activity.

**Scheme 2:**
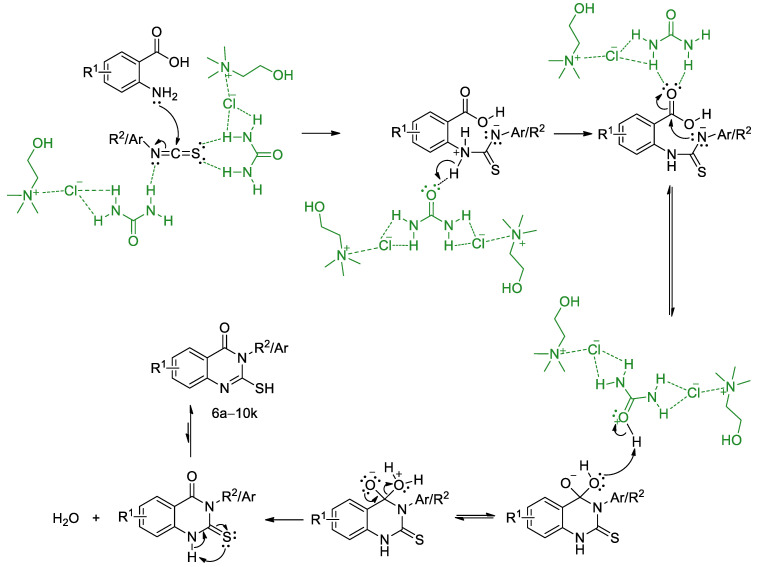
A plausible mechanism for the synthesis of substituted 2-mercaptoquinazolin-4(3*H*)-ones.

According to the conducted experiments and reported data [31], a plausible mechanism for the condensation of anthranilic acids **1–5** and isothiocyanates is presented in Scheme 2. The first step includes a nucleophilic attack of a nitrogen atom to an electron-deficient carbon atom from isothiocyanate in the presence of choline chloride:urea (1:2) DES (marked green on Scheme 2). Subsequently, the intermediate undergoes cyclization and the required quinazolinone is formed through dehydration.

The greenness of this process is presented by calculation of the Sheldon’s *E*-*factor*:

(1) $$E-factor=\frac{kg\;\left(waste\right)}{kg\;\left(product\right)}\;=\frac{0.00013\;\mathrm{kg}}{\;0.000265\;\mathrm{kg}}=0.49$$

## 3. Experimental Section

### 3.1. Materials and Methods

All chemicals and solvents were purchased from commercial suppliers and used without purification. Choline chloride (99%), *N*-methylurea (97%), 1,3-dimethylurea (98%), thiourea (99+%), acetamide (99%), butane-1,4-diol (99+%), xylitol (99+%), oxalic acid (98%, anhydrous), levulinic acid (98+%), malic acid (99+%), malonic acid (99%), anthranilic acid (98+%), 5-iodoanthranilic acid (98%), 5-bromoanthranilic acid (97%), 4-chloroanthranilic acid (98%), methyl isothiocyanate (97%), ethyl isothiocyanate (96%), allyl isothiocyanate (94% stabilized with 0.01% α-tocopherol), phenyl isothiocyanate (98%), benzyl isothiocyanate (98%) were purchased from Acros Organics (Geel, Belgium). 4-Methylphenyl isothiocyanate (97%), 3,5-dichloroanthranilic acid (97%) were purchased from Alfa Aesar (Lancashire, UK). 3-Bromophenyl isothiocyanate (97%), 3-methoxyphenyl isothiocyanate (987+%) were purchased from Maybridge (Maybridge Chemical Company Ltd., Altrincham, UK). Urea (p.a.), glycerol (redistilled, p.a.), D(+)-glucose (p.a.), citric acid (p.a.) were purchased from Gram Mol. Ethane-1,2-diol (p.a.) was purchased from Carlo Erba (Carlo Erba Reagents GmbH, Emmendingen, Germany). Sorbitol (lab. reag. grade), D(+)-fructose (lab. reag. grade), L(+)-tartaric acid (lab. reag. grade) were purchased from Fischer Scientific (Fisher Scientific UK Ltd., Loughborough, UK). 4-Fluorophenyl isothiocyanate (98%), 4-chlorophenyl isothiocyanate (99%), 3-chlorophenyl isothiocyanate (98%), maleic acid (reag. plus. ≥99% HPLC) was purchased from Sigma Aldrich (Sigma Aldrich Chemie GmbH, München, Germany). Lactic acid (min. 88%, p.a.) was purchased from T.T.T. (T.T.T. d.o.o., Sveta Nedelja, Croatia). Melting points (uncorrected) were determined in open capillaries on Electrothermal melting point apparatus (Electrothermal Engineering Ltd., Rochford, UK). For monitoring the progress of a reaction, thin layer chromatography (TLC) was performed on pre-coated Merck (Darmstadt, Germany) silica gel 60F-254 plates using benzene:acetic acid:acetone (8:1:1, *v/v*) as solvent system and the spots were detected under UV light (254 nm). ^1^H and ^13^C NMR spetra were recorded on a Bruker 600 MHz spectrometer (Bruker Biospin, Rheinstetten, Germany) at 298 K in dimethylsulfoxide (DMSO-*d*_6_). The synthesis by mixing and heating was performed on a magnetic stirrer (IKA RH basic 2, Staufen, Germany). Microwave-assisted synthesis was performed using Milestone flexiWAVE reactor (Milestones Srl, Milan, Italy) with the power at 1800 W. Ultrasound-assisted synthesis was performed in temperature controlled ultrasonic bath (Elma P70 H, Singen, Germany) with frequency at 37 Hz and the power at 50 W.

### 3.2. Preparation of DESs

DESs were prepared by mixing choline chloride with appropriate amides, carbohydrates, alcohols and carboxylic acids in a different molar ratio, as listed in Table 1. The mixtures were heated at 80 °C with continuous stirring until a clear liquid was obtained. The prepared DESs was used as such as described in Section 3.3, Section 3.4 and Section 3.5.

### 3.3. Synthesis of Substituted 2-Mercaptoquinazolin-4(3H)-Ones by Mixing and Heating Preparation of DESs

Anthranilic acid **1–5** (1 mmol) and corresponding isothiocyanate (1.2 mmol) were added to 10 mL of DES and stirred at 80 °C for 1 h. Upon completion of the reaction, water was added, and the precipitated product was filtered off. When necessary, a recrystallization was performed using ethanol.

### 3.4. Microwave-Assisted Synthesis of Substituted 2-Mercaptoquinazolin-4(3H)-ones

Anthranilic acid **1–5** (1 mmol) and corresponding isothiocyanate (1.2 mmol) in 10 mL of DES were stirred under microwave irradiation (1800 W) at 80 °C for 1 h. Upon completion of the reaction, water was added, and the precipitated product was filtered.

### 3.5. Ultrasound-Assisted Synthesis of Substituted 2-Mercaptoquinazolin-4(3H)-ones

Anthranilic acid **1–5** (1 mmol) and corresponding isothiocyanate (1.2 mmol) in 10 mL of DES were subjected to ultrasonic irradiation at 50 W and 80 °C for 1 h. Upon completion of the reaction, water was added, and the precipitated product was filtered.

### 3.6. Recycling of DES

5-Bromoanthranilic acid **3** (216 mg; 1 mmol) and *p*-tolyl isothiocyanate (165 µL; 1.2 mmol) in 10 mL of DES were mixed and heated on magnetic stirrer at 80 °C for 1 h. Upon addition of the water, a crude product was precipitated, and the water was evaporated. The procedure was repeated four times. The Sheldon’s *E*-*factor* was calculated according to the following formula as described in our previous work [43]:

(2) $$E-factor=\frac{kg\;\left(waste\right)}{kg\;\left(product\right)}\;$$

### 3.7. Characterization of Compounds

Spectra Are Shown in Supplementary Materials.

#### 3.7.1. 2-Mercapto-3-methylquinazolin-4(3*H*)-one (**6a**)

Pale yellow powder; mp = 264–265 °C (262–264 °C [48]); *R*_f_ = 0.79; ^1^H NMR (DMSO-*d*_6_, 400 MHz) δ 12.9 (1H, s, –SH), 7.93 (1H, d, *J* = 7.54 Hz, arom.), 7.73 (1H, m, arom.), 7.37 (1H, d, *J* = 7.91 Hz, arom.), 7.32 (1H, t, *J* = 7.54 Hz, arom.), 3.64 (3H, s, -CH_3_). ^13^C NMR (CDCl_3_, 75 MHz) δ 175.3, 159.6, 138.9, 135.3, 127.2, 124.3, 115.5, 33.2.

#### 3.7.2. 3-Ethyl-2-mercaptoquinazolin-4(3*H*)-one (**6b**)

Pale yellow powder; mp = 255 °C (246–248 °C [50]); *R*_f_ = 0.82; ^1^H NMR (DMSO-*d*_6_, 300 MHz) δ 12.9 (1H, s, -SH), 7.93–7.96 (1H, m, arom.), 7.69–7.75 (1H, m, arom.), 7.36 (1H, d, *J* = 8.29 Hz, arom.), 7.29–7.34 (1H, m, arom.), 4.41–4.48 (2H, q, *J* = 7.16 Hz, -CH_2_-), 1.20–1.25 (3H, t, *J* = 6.97 Hz, -CH_3_). ^13^C NMR (CDCl_3_, 75 MHz) δ 174.7, 158.9, 138.9, 135.3, 127.2, 124.4, 115.5, 99.5, 11.9.

#### 3.7.3. 3-Allyl-2-mercaptoquinazolin-4(3*H*)-one (**6c**)

Pale yellow powder; mp = 206–208 °C (206 °C [33]); *R*_f_ = 0.77; ^1^H NMR (DMSO-*d*_6_, 300 MHz) δ 12.9 (1H, s, -SH), 7.94–7.96 (1H, d, *J* = 7.54 Hz, arom.), 7.71–7.76 (1H, m, arom.), 7.36–7.41 (1H, d, *J* = 8.29 Hz, arom.), 7.30–7.33 (1H, t, *J* = 7.54, 7.54 Hz, arom.), 5.87–5.96 (1H, m, -CH-), 5.17 (2H, d, *J* = 6.97 Hz, -CH=), 5.04 (2H, d, *J* = 4.90 Hz, =CH_2_). ^13^C NMR (CDCl_3_, 75 MHz) δ 175.0, 158.9, 139.1, 135.5, 131.8, 127.3, 124.5, 117.1, 115.6, 99.5, 47.6.

#### 3.7.4. 2-Mercapto-3-phenylquinazolin-4(3*H*)-one (**6d**)

White powder; mp = 304–305 °C (300–302 °C [48]); *R*_f_ = 0.80; ^1^H NMR (DMSO-*d*_6_, 300 MHz) δ 13.0 (1H, s, -SH), 7.97 (1H, dd, *J* = 7.91, 1.13 Hz, arom.), 7.76–7.81 (1H, t, arom.), 7.29–7.51 (7H, m, arom.). ^13^C NMR (CDCl_3_, 75 MHz) δ 176.0, 159.8, 139.6, 139.3, 135.6, 128.9, 128.1, 127.4, 124.3, 115.7.

#### 3.7.5. 3-Benzyl-2-mercaptoquinazolin-4(3*H*)-one (**6e**)

White powder; mp = 248–250 °C (231–233 °C [48]); *R*_f_ = 0.77; ^1^H NMR (DMSO-*d*_6_, 300 MHz) δ 13.1 (1H, s, -SH), 7.95–7.97 (1H, m, arom.), 7.73–7.79 (1H, m, arom.), 7.42–7.44 (1H, d, *J* = 8.29 Hz, arom.), 7.23–7.38 (7H, m, arom.), 5.67 (2H, s, -CH_2_-). ^13^C NMR (CDCl_3_, 75 MHz) δ 175.5, 159.4, 139.1, 136.6, 128.2, 127.1, 124.6, 115.7, 48.7.

#### 3.7.6. 2-Mercapto-3-(p-tolyl)quinazolin-4(3H)-one (6f)

White powder; mp = 312–313 °C (318–320 °C [50]); *R*_f_ = 0.76; ^1^H NMR (DMSO-*d*_6_, 300 MHz) δ 13.0 (1H, s, -SH), 7.95 (1H, d, *J* = 7.54 Hz, arom.), 7.75–7.78 (1H, m, arom.), 7.45 (1H, d, *J* = 7.91 Hz, arom.), 7.26–7.37 (3H, m, arom.), 7.13–7.15 (2H, d, *J* = 7.91 Hz, arom.), 2.37 (3H, s, -CH_3_). ^13^C NMR (CDCl_3_, 75 MHz) δ 176.2, 159.8, 139.5, 137.3, 135.5, 130.3, 129.4, 128.6, 127.4, 125.7, 124.3, 116.1, 115.7, 20.8.

#### 3.7.7. 3-(4-Fluorophenyl)-2-mercaptoquinazolin-4(3*H*)-one (**6g**)

White powder; mp = 336–337 °C (>300 °C [3]); *R*_f_ = 0.76; ^1^H NMR (DMSO-*d*_6_, 300 MHz) δ 13.1 (1H, s, -SH), 7.95 (1H, m, arom.), 7.76–7.81 (1H, m, arom.), 7.44–7.47 (1H, d, *J* = 7.91 Hz, arom.), 7.27–7.38 (5H, m, arom.). ^13^C NMR (CDCl_3_, 75 MHz) δ 176.1, 163.1, 159.9, 139.5, 135.6, 131.0, 127.4, 124.3, 115.7, 99.5.

#### 3.7.8. 3-(4-Chlorophenyl)-2-mercaptoquinazolin-4(3*H*)-one (**6h**)

White powder; mp = 331–332 °C (>300 °C [51]); *R*_f_ = 0.75; ^1^H NMR (DMSO-*d*_6_, 300 MHz) δ 13.1 (1H, s, -SH), 7.95 (1H, d, *J* = 7.91 Hz, arom.), 7.76–7.82 (1H, m, arom.), 7.53–7.56 (2H, m, arom.), 7.44–7.47 (1H, m, arom.), 7.33–7.35 (3H, m, arom.). ^13^C NMR (CDCl_3_, 75 MHz) δ 175.9, 159.8, 139.6, 138.2, 135.6, 132.7, 131.0, 129.0, 127.4, 124.4, 116.2, 115.7, 99.5.

#### 3.7.9. 3-(4-Bromophenyl)-2-mercaptoquinazolin-4(3*H*)-one (**6i**)

White powder; mp = 330–331 °C (300–301 °C [55]); *R*_f_ = 0.78; ^1^H NMR (DMSO-*d*_6_, 300 MHz) δ 13.1 (1H, s, -SH), 7.95 (1H, dd, *J* = 7.91, 0.75 Hz, arom.), 7.76–7.79 (1H, m, arom.), 7.57–7.70 (2H, m, arom.), 7.44–7.47 (1H, d, *J* = 8.29 Hz, arom.), 7.33–7.35 (1H, m, arom.), 7.27–7.30 (2H, d, arom.). ^13^C NMR (CDCl_3_, 75 MHz) δ 175.8, 159.8, 139.6, 138.7, 135.6, 132.1, 131.2, 127.4, 124.4, 121.3, 116.2, 115.7.

#### 3.7.10. 2-Mercapto-3-(3-methoxyphenyl)quinazolin-4(3*H*)-one (**6j**)

White powder; mp = 285 °C (248–249 °C [54]); *R*_f_ = 0.75; ^1^H NMR (DMSO-*d*_6_, 300 MHz) δ 13.0 (1H, s, -SH), 7.95 (1H, m, arom.), 7.78 (1H, m, arom.), 7.38–7.46 (3H, m, arom.), 7.0 (1H, dd, *J* = 7.91, 2.26 Hz, arom.), 6.84–6.91 (2H, m, arom.), 3.76 (3H, s, -OCH_3_). ^13^C NMR (CDCl_3_, 75 MHz) δ 175.9, 159.8, 140.3, 139.6, 135.5, 129.4, 127.4, 124.4, 121.2, 116.2, 115.6, 114.9, 113.6, 99.5, 55.2.

#### 3.7.11. 3-(3-Chlorophenyl)-2-mercaptoquinazolin-4(3*H*)-one (**6k**)

White powder; mp = 300–301 °C (231–233 °C [54]); *R*_f_ = 0.74; ^1^H NMR (DMSO-*d*_6_, 300 MHz) δ 13.1 (1H, s, -SH), 7.95 (1H, m, arom.), 7.77 (1H, m, arom.), 7.28–7.52 (6H, m, arom.). ^13^C NMR (CDCl_3_, 75 MHz) δ 175.8, 159.7, 140.6, 139.6, 135.6, 132.9, 130.6, 129.4, 129.2, 128.2, 128.1, 127.4, 124.4, 116.2, 115.7.

#### 3.7.12. 6-Iodo-2-mercapto-3-methylquinazolin-4(3*H*)-one (**7a**)

Pale yellow powder; mp = 307–308 °C (271 °C [33]); *R*_f_ = 0.80; ^1^H NMR (DMSO-*d*_6_, 300 MHz) δ 12.99 (1H, s, -SH), 8.16 (1H, s, arom.), 7.99 (1H, dd, *J* = 8.67, 1.88 Hz, arom.), 7.15–7.18 (1H, d, *J* = 8.29, arom.), 3.63 (3H, s, -CH_3_). ^13^C NMR (CDCl_3_, 75 MHz) δ 175.3, 158.3, 143.2, 138.4, 135.2, 117.9, 117.2, 87.8, 33.3.

#### 3.7.13. 3-Ethyl-6-iodo-2-mercaptoquinazolin-4(3*H*)-one (**7b**)

White powder; mp = 290–292 °C (281 °C [33]); *R*_f_ = 0.85; ^1^H NMR (DMSO-*d*_6_, 300 MHz) δ 12.96 (1H, s, -SH), 8.17 (1H, s, arom.), 8.00 (1H, d, *J* = 8.67 Hz, arom.), 7.15–7.18 (1H, d, *J* = 8.67 Hz, arom.), 4.38–4.45 (2H, q, *J* = 6.78 Hz, -CH_2_-), 1.19–1.23 (1H, t, *J* = 6.78 Hz, -CH_3_). ^13^C NMR (CDCl_3_, 75 MHz) δ 174.7, 157.7, 143.4, 138.4, 135.2, 117.9, 117.5, 87.9, 41.4, 11.8.

#### 3.7.14. 3-Allyl-6-iodo-2-mercaptoquinazolin-4(3*H*)-one (**7c**)

Pale yellow powder; mp = 234–235 °C (219 °C [33]); *R*_f_ = 0.88; ^1^H NMR (DMSO-*d*_6_, 300 MHz) δ 13.02 (1H, s, -SH), 8.17 (1H, d, *J* = 2.26 Hz, arom.), 8.01–8.05 (1H, dd, *J* = 8.67, 1.88 Hz, arom.), 7.17–7.20 (1H, d, *J* = 8.67 Hz, arom.), 5.89 (1H, m, -CH=), 5.12–5.18 (2H, m, -CH_2_-), 5.00–5.02 (2H, d, *J* = 5.27 Hz, =CH_2_). ^13^C NMR (CDCl_3_, 75 MHz) δ 174.9, 157.8, 143.6, 138.5, 135.2, 131.5, 117.9, 117.3, 87.9, 47.8.

#### 3.7.15. 6-Iodo-2-mercapto-3-phenylquinazolin-4(3*H*)-one (**7d**)

White powder; mp = 350–352 °C (>300 °C [33]); *R*_f_ = 0.86; ^1^H NMR (DMSO-*d*_6_, 300 MHz) δ 13.01 (1H, s, -SH), 8.17 (1H, d, *J* = 1.47 Hz, arom.), 8.06–8.08 (1H, dd, *J* = 8.44, 1.83 Hz, arom.), 7.46–7.49 (2H, m, arom.), 7.41 (1H, m, arom.), 7.24–7.27 (3H, m, arom.). ^13^C NMR (CDCl_3_, 75 MHz) δ 176.0, 158.6, 143.6, 139.1, 135.2, 128.9, 128.2, 118.2, 117.9, 87.7.

#### 3.7.16. 3-Benzyl-6-iodo-2-mercaptoquinazolin-4(3*H*)-one (**7e**)

White powder; mp = 352 °C; *R*_f_ = 0.89; ^1^H NMR (DMSO-*d*_6_, 300 MHz) δ 13.12 (1H, s, -SH), 8.18 (1H, d, *J* = 1.88 Hz, arom.), 8.03–8.07 (1H, dd, *J* = 8.48, 2.07 Hz, arom.), 7.20–7.33 (6H, m, arom.), 5.63 (2H, s, -CH_2_-). ^13^C NMR (CDCl_3_, 75 MHz) δ 175.5, 158.2, 143.6, 138.5, 136.3, 135.2, 128.2, 127.0, 117.9, 117.4, 88.1, 48.8.

#### 3.7.17. 6-Iodo-2-mercapto-3-(p-*t*olyl)quinazolin-4(3*H*)-one (**7f**)

White powder; mp = 350–351 °C; *R*_f_ = 0.87; ^1^H NMR (DMSO-*d*_6_, 300 MHz) δ 13.06 (1H, s, -SH), 8.17 (1H, d, *J* = 1.47 Hz, arom.), 8.06–8.08 (1H, dd, *J* = 8.80, 2.20 Hz, arom.), 7.27 (2H, d, *J* = 1.47 Hz, arom.), 7.24 (1H, d, *J* = 8.80 Hz, arom.), 7.12–7.13 (2H, d, *J* = 8.07 Hz, arom.), 2.37 (3H, s, -CH_3_). ^13^C NMR (CDCl_3_, 75 MHz) δ 176.1, 158.6, 143.6, 138.9, 137.5, 136.5, 135.2, 129.4, 128.5, 118.2, 117.9, 87.6, 20.8.

#### 3.7.18. 3-(4-Fluorophenyl)-6-iodo-2-mercaptoquinazolin-4(3*H*)-one (**7g**)

Pale yellow powder; mp = 349–350 °C; *R*_f_ = 0.77; ^1^H NMR (DMSO-*d*_6_, 300 MHz) δ 13.12 (1H, s, -SH), 8.17 (1H, d, *J* = 1.88 Hz, arom.), 8.06–8.09 (1H, dd, *J* = 8.48, 2.07 Hz, arom.), 7.31–7.33 (3H, m, arom.), 7.23–7.26 (1H, m, arom.). ^13^C NMR (CDCl_3_, 75 MHz) δ 176.1, 163.2, 159.9, 158.6, 143.8, 138.9, 135.3, 131.1, 118.2, 117.9, 115.9, 115.7, 87.7, 20.8.

#### 3.7.19. 3-(4-Chlorophenyl)-6-iodo-2-mercaptoquinazolin-4(3*H*)-one (**7h**)

Pale yellow powder; mp = 337–339 °C (320–321 °C [29]); *R*_f_ = 0.84; ^1^H NMR (DMSO-*d*_6_, 300 MHz) δ 13.14 (1H, s, -SH), 8.17 (1H, d, *J* = 1.88 Hz, arom.), 8.06–8.09 (1H, dd, *J* = 8.67, 1.88 Hz, arom.), 7.53–7.56 (2H, d, *J* = 8.67 Hz, arom.), 7.31–7.34 (2H, d, *J* = 8.67 Hz, arom.), 7.23–7.26 (1H, d, *J* = 8.29 Hz, arom.). ^13^C NMR (CDCl_3_, 75 MHz) δ 175.9, 158.5, 143.6, 138.9, 138.0, 135.2, 132.8, 130.9, 129.0, 118.2, 117.9, 87.7.

#### 3.7.20. 3-(4-Bromophenyl)-6-iodo-2-mercaptoquinazolin-4(3*H*)-one (**7i**)

White powder; mp = 355–357 °C; *R*_f_ = 0.87; ^1^H NMR (DMSO-*d*_6_, 300 MHz) δ 13.14 (1H, s, -SH), 8.17 (1H, d, *J* = 1.88 Hz, arom.), 8.07–8.10 (1H, dd, *J* = 8.48, 2.07 Hz, arom.), 7.67–7.70 (2H, m, arom.), 7.22–7.27 (3H, m, arom.). ^13^C NMR (CDCl_3_, 75 MHz) δ 175.8, 158.5, 143.6, 138.9, 138.5, 135.2, 132.1, 131.3, 121.4, 118.2, 117.9, 99.5, 87.7.

#### 3.7.21. 6-Iodo-2-mercapto-3-(3-methoxyphenyl)quinazolin-4(3*H*)-one (**7j**)

White powder; mp = 314 °C; *R*_f_ = 0.71; ^1^H NMR (DMSO-*d*_6_, 300 MHz) δ 13.08 (1H, s, -SH), 8.17 (1H, d, *J* = 1.88 Hz, arom.), 8.06–8.09 (1H, dd, *J* = 8.67, 2.26 Hz, arom.), 7.36–7.41 (1H, m, arom.), 7.22–7.25 (1H, d, *J* = 8.67 Hz, arom.), 7.00 (1H, dd, *J* = 8.10, 2.07 Hz, arom.), 6.84–6.90 (1H, t, *J* = 2.07 Hz, arom.), 6.85 (1H, d, *J* = 7.54 Hz, arom.), 3.76 (3H, s, -OCH_3_). ^13^C NMR (CDCl_3_, 75 MHz) δ 175.9, 159.8, 158.4, 143.6, 140.1, 138.9, 135.2, 129.6, 121.1, 117.9, 114.8, 113.7, 87.7, 55.3.

#### 3.7.22. 3-(3-Chlorophenyl)-6-iodo-2-mercaptoquinazolin-4(3*H*)-one (**7k**)

White powder; mp = 313–314 °C; *R*_f_ = 0.83; ^1^H NMR (DMSO-*d*_6_, 300 MHz) δ 13.15 (1H, s, -SH), 8.18 (1H, d, *J* = 1.88 Hz, arom.), 8.07–8.10 (1H, dd, *J* = 8.67, 1.88 Hz, arom.), 7.46–7.52 (3H, m, arom.), 7.23–7.31 (2H, m, arom.). ^13^C NMR (CDCl_3_, 75 MHz) δ 175.8, 158.5, 143.7, 140.4, 139.1, 135.2, 132.9, 130.5, 129.12, 128.4, 128.0, 118.2, 117.9, 87.8.

#### 3.7.23. 6-Bromo-2-mercapto-3-methylquinazolin-4(3*H*)-one (**8a**)

White powder; mp = 280–281 °C (270 °C [49]); *R*_f_ = 0.81; ^1^H NMR (DMSO-*d*_6_, 300 MHz) δ 13.02 (1H, s, -SH), 7.99 (1H, d, *J* = 1.88 Hz, arom.), 7.89 (1H, dd, *J* = 8.67, 2.26 Hz, arom.), 7.31–7.33 (1H, d, *J* = 8.67 Hz, arom.), 3.63 (3H, s, -CH_3_). ^13^C NMR (CDCl_3_, 75 MHz) δ 175.3, 158.5, 138.1, 137.9, 129.1, 118.1, 116.9, 115.9, 33.3.

#### 3.7.24. 6-Bromo-3-ethyl-2-mercaptoquinazolin-4(3*H*)-one (**8b**)

White powder; mp = 243–244 °C; *R*_f_ = 0.88; ^1^H NMR (DMSO-*d*_6_, 300 MHz) δ 12.99 (1H, s, -SH), 7.99 (1H, d, *J* = 1.88 Hz, arom.), 7.86–7.89 (1H, dd, *J* = 8.67, 2.26 Hz, arom.), 7.29–7.33 (1H, d, *J* = 8.67 Hz, arom.), 4.38–4.45 (2H, q, *J* = 6.78 Hz, -CH_2_-), 1.19–1.24 (3H, t, *J* = 6.97, -CH_3_). ^13^C NMR (CDCl_3_, 75 MHz) δ 175.3, 158.5, 138.1, 137.9, 129.1, 118.1, 116.9, 115.9, 33.3.

#### 3.7.25. 3-Allyl-6-bromo-2-mercaptoquinazolin-4(3*H*)-one (**8c**)

White powder; mp = 242–243 °C; *R*_f_ = 0.85; ^1^H NMR (DMSO-*d*_6_, 300 MHz) δ 13.06 (1H, s, -SH), 8.02 (1H, d, *J* = 2.26 Hz, arom.), 7.89–7.93 (1H, dd, *J* = 8.85, 2.07 Hz, arom.), 7.32–7.35 (1H, d, *J* = 8.67 Hz, arom.), 5.90 (1H, qd, *J* = 10.99, 5.09 Hz, -CH=), 5.16–5.19 (2H, m, *J* = 6.97 Hz, -CH_2_-), 5.01 (2H, d, *J* = 5.27, =CH_2_). ^13^C NMR (CDCl_3_, 75 MHz) δ 175.3, 158.5, 138.1, 137.9, 129.1, 118.1, 116.9, 115.9, 33.3.

#### 3.7.26. 6-Bromo-2-mercapto-3-phenylquinazolin-4(3*H*)-one (**8d**)

White powder; mp = 351–353 °C (322 °C [49]); *R*_f_ = 0.76; ^1^H NMR (DMSO-*d*_6_, 300 MHz) δ 13.14 (1H, s, -SH), 8.01 (1H, d, *J* = 2.26 Hz, arom.), 7.93–7.97 (1H, dd, *J* = 9.04, 2.26 Hz, arom.), 7.38–7.51 (4H, m, arom.), 7.26–7.29 (2H, d, *J* = 7.16 Hz, arom.). ^13^C NMR (CDCl_3_, 75 MHz) δ 176.1, 158.7, 139.1, 138.2, 129.3, 128.9, 128.8, 128.2, 118.1, 115.9.

#### 3.7.27. 3-Benzyl-6-bromo-2-mercaptoquinazolin-4(3*H*)-one (**8e**)

White powder; mp = 244 °C; *R*_f_ = 0.88; ^1^H NMR (DMSO-*d*_6_, 300 MHz) δ 13.16 (1H, s, -SH), 8.01 (1H, d, *J* = 1.88 Hz, arom.), 7.90–7.94 (1H, dd, *J* = 8.67, 2.26 Hz, arom.), 7.22–7.38 (6H, m, arom.), 5.65 (2H, d, *J* = 7.16 Hz, -CH_2_-). ^13^C NMR (CDCl_3_, 75 MHz) δ 175.5, 158.4, 138.2, 136.3, 129.39, 128.2, 127.1, 126.9, 118.1, 117.2, 116.2, 48.8.

#### 3.7.28. 6-Bromo-2-mercapto-3-(*p*-tolyl)quinazolin-4(3*H*)-one (**8f**)

White powder; mp = 341–342 °C (> 300 °C [56]); *R*_f_ = 0.82; ^1^H NMR (DMSO-*d*_6_, 300 MHz) δ 13.10 (1H, s, -SH), 8.01 (1H, d, *J* = 2.20 Hz, arom.), 7.93–7.95 (1H, dd, *J* = 8.80, 2.20 Hz, arom.), 7.37–7.39 (1H, d, *J* = 8.80 Hz, arom.), 7.26–7.28 (1H, d, *J* = 8.07 Hz, arom.), 7.12–7.14 (2H, m, arom.), 2.37 (3H, s, -CH_3_). ^13^C NMR (CDCl_3_, 75 MHz) δ 176.2, 158.7, 138.7, 138.1, 137.5, 136.5, 129.4, 128.5, 118.1, 117.9, 115.8, 20.8.

#### 3.7.29. 6-Bromo-3-(4-fluorophenyl)-2-mercaptoquinazolin-4(3*H*)-one (**8g**)

White powder; mp = 354–355 °C; *R*_f_ = 0.81; ^1^H NMR (DMSO-*d*_6_, 300 MHz) δ 13.15 (1H, s, -SH), 8.01 (1H, d, *J* = 2.26 Hz, arom.), 7.94–7.97 (1H, dd, *J* = 8.85, 2.45 Hz, arom.), 7.31–7.41 (5H, m, arom.). ^13^C NMR (CDCl_3_, 75 MHz) δ 176.1, 163.2, 159.9, 158.8, 138.7, 138.3, 135.3, 131.3, 130.8, 129.3, 118.1, 115.9, 115.6.

#### 3.7.30. 6-Bromo-3-(4-chlorophenyl)-2-mercaptoquinazolin-4(3*H*)-one (**8h**)

White powder; mp = 344–345 °C; *R*_f_ = 0.81; ^1^H NMR (DMSO-*d*_6_, 300 MHz) δ 13.175 (1H, s, -SH), 8.01 (1H, d, *J* = 2.20 Hz, arom.), 7.94–7.96 (1H, dd, *J* = 8.80, 2.40 Hz, arom.), 7.54–7.56 (2H, m, arom.), 7.38–7.40 (1H, m, arom.), 7.32–7.34 (2H, m, arom.). ^13^C NMR (CDCl_3_, 75 MHz) δ 175.8, 158.7, 138.7, 138.2, 137.9, 132.8, 130.9, 129.3, 129.0, 118.1, 118.0, 115.9.

#### 3.7.31. 6-Bromo-3-(4-bromophenyl)-2-mercaptoquinazolin-4(3*H*)-one (**8i**)

White powder; mp = 349–350 °C; *R*_f_ = 0.77; ^1^H NMR (DMSO-*d*_6_, 300 MHz) δ 13.18 (1H, s, -SH), 8.02 (1H, d, *J* = 2.26 Hz, arom.), 7.94–7.98 (1H, dd, *J* = 8.67, 2.26 Hz, arom.), 7.67–7.70 (2H, m, arom.), 7.41 (1H, d, *J* = 9.04 Hz, arom.), 7.26–7.29 (2H, d, *J* = 8.29 Hz, arom.). ^13^C NMR (CDCl_3_, 75 MHz) δ 175.8, 158.7, 138.7, 138.2, 137.9, 132.8, 130.9, 129.3, 129.0, 118.1, 118.0, 115.9.

#### 3.7.32. 6-Bromo-2-mercapto-3-(3-methoxyphenyl)quinazolin-4(3*H*)-one (**8j**)

White powder; mp = 312–313 °C; *R*_f_ = 0.73; ^1^H NMR (DMSO-*d*_6_, 300 MHz) δ 13.11 (1H, s, -SH), 8.00 (1H, d, *J* = 2.20 Hz, arom.), 7.93–7.95 (1H, dd, *J* = 8.80, 2.20 Hz, arom.), 7.37–7.40 (2H, m, arom.), 6.99 (1H, dd, *J* = 8.07, 2.20 Hz, arom.), 6.90 (1H, t, *J* = 2.20, 2.20 Hz, arom.), 6.84–6.86 (1H, m, arom.), 3.76 (3H, s, -OCH_3_). ^13^C NMR (CDCl_3_, 75 MHz) δ 175.9, 159.8, 158.6, 140.1, 138.7, 138.1, 129.6, 129.2, 121.1, 118.1, 118.0, 115.8, 114.8, 113.7, 55.2.

#### 3.7.33. 6-Bromo-3-(3-chlorophenyl)-2-mercaptoquinazolin-4(3*H*)-one (**8k**)

White powder; mp = 305–307 °C; *R*_f_ = 0.81; ^1^H NMR (DMSO-*d*_6_, 300 MHz) δ 13.19 (1H, s, -SH), 8.01 (1H, dd, *J* = 8.67, 2.26 Hz, arom.), 7.95–7.98 (1H, m, arom.), 7.47–7.52 (3H, m, arom.), 7.38–7.41 (1H, d, *J* = 8.67 Hz, arom.), 7.28–7.30 (1H, dt, *J* = 6.69, 1.93, 1.93 Hz, arom.). ^13^C NMR (CDCl_3_, 75 MHz) δ 175.8, 158.7, 140.3, 138.7, 138.4, 138.2, 132.9, 130.6, 129.2, 128.3, 127.8, 118.1, 115.9.

#### 3.7.34. 7-Chloro-2-mercapto-3-methylquinazolin-4(3*H*)-one (**9a**)

White powder; mp = 327–328 °C; *R*_f_ = 0.87; ^1^H NMR (DMSO-*d*_6_, 300 MHz) δ 12.99 (1H, s, -SH), 7.96 (1H, d, *J* = 8.07 Hz, arom.), 7.40 (1H, d, *J* = 1.47 Hz, arom.), 7.37 (1H, dd, *J* = 8.44, 1.83 Hz, arom.), 3.64 (3H, s, -CH_3_). ^13^C NMR (CDCl_3_, 75 MHz) δ 175.8, 158.9, 139.9, 139.7, 129.5, 124.6, 114.9, 114.2, 33.3.

#### 3.7.35. 7-Chloro-3-ethyl-2-mercaptoquinazolin-4(3*H*)-one (**9b**)

White powder; mp = 265 °C; *R*_f_ = 0.91; ^1^H NMR (DMSO-*d*_6_, 300 MHz) δ 12.91 (1H, s, -SH), 7.90–7.93 (1H, d, *J* = 8.67 Hz, arom.), 7.31–7.35 (2H, m, arom.), 4.38–4.44 (2H, q, *J* = 6.78 Hz, -CH2-), 1.19–1.23 (3H, t, *J* = 6.78 Hz, -CH_3_). ^13^C NMR (CDCl_3_, 75 MHz) δ 175.2, 158.3, 139.8, 139.7, 129.3, 124.6, 124.5, 114.9, 114.4, 41.3, 11.8.

#### 3.7.36. 3-Allyl-7-chloro-2-mercaptoquinazolin-4(3*H*)-one (**9c**)

White powder; mp = 248–249 °C; *R*_f_ = 0.89; ^1^H NMR (DMSO-*d*_6_, 300 MHz) δ 12.99 (1H, s, -SH), 7.92–7.94 (1H, d, *J* = 8.67 Hz, arom.), 7.33–7.38 (2H, m, arom.), 5.88–5.90 (1H, m, -CH=), 5.13–5.19 (2H, m, -CH_2_-), 4.99–5.02 (2H, d, *J* = 4.90 Hz, =CH_2_). ^13^C NMR (CDCl_3_, 75 MHz) δ 175.5, 158.3, 139.8, 129.6, 129.3, 117.0, 115.0, 114.3, 47.8.

#### 3.7.37. 7-Chloro-2-mercapto-3-phenylquinazolin-4(3*H*)-one (**9d**)

White powder; mp = 313 °C (288–289 °C [51]); *R*_f_ = 0.84; ^1^H NMR (DMSO-*d*_6_, 300 MHz) δ 13.08 (1H, s, -SH), 7.93–7.96 (1H, d, *J* = 8.67 Hz, arom.), 7.36–7.51 (5H, m, arom.), 7.27–7.29 (2H, m,arom.). ^13^C NMR (CDCl_3_, 75 MHz) δ 176.5, 158.3, 140.4, 139.9, 139.0, 129.6, 128.9, 128.2, 124.4, 115.2, 114.9.

#### 3.7.38. 3-Benzyl-7-chloro-2-mercaptoquinazolin-4(3*H*)-one (**9e**)

White powder; mp = 270–272 °C (255–257 °C [51]); *R*_f_ = 0.90; ^1^H NMR (DMSO-*d*_6_, 300 MHz) δ 13.09 (1H, s, -SH), 7.93–7.96 (1H, d, *J* = 8.29 Hz, arom.), 7.23–7.42 (7H, m, arom.), 5.63 (2H, s, -CH_2_-). ^13^C NMR (CDCl_3_, 75 MHz) δ 175.9, 158.3, 136.3, 129.5, 128.3, 128.2, 127.2, 127.1, 124.6, 115.0, 114.3, 48.7.

#### 3.7.39. 7-Chloro-2-mercapto-3-(*p*-tolyl)quinazolin-4(3*H*)-one (**9f**)

White powder; mp = 307–309 °C (278–280 °C [51]); *R*_f_ = 0.87; ^1^H NMR (DMSO-*d*_6_, 300 MHz) δ 13.05 (1H, s, -SH), 7.92–7.95 (1H, d, *J* = 8.67 Hz, arom.), 7.45 (1H, d, *J* = 1.88 Hz, arom.), 7.35–7.38 (1H, dd, *J* = 8.29, 1.88 Hz, arom.), 7.26–7.29 (2H, d, *J* = 8.29 Hz, arom.), 7.13–7.15 (2H, d, *J* = 8.29 Hz, arom.), 2.37 (3H, s, -CH_3_). ^13^C NMR (CDCl_3_, 75 MHz) δ 176.6, 158.3, 140.4, 139.8, 137.5, 136.5, 129.8, 129.6, 128.5, 124.3, 115.1, 114.9, 20.8.

#### 3.7.40. 7-Chloro-3-(4-fluorophenyl)-2-mercaptoquinazolin-4(3*H*)-one (**9g**)

White powder; mp = 314–315 °C; *R*_f_ = 0.86; ^1^H NMR (DMSO-*d*_6_, 300 MHz) δ 13.09 (1H, s, -SH), 7.93–7.95 (1H, d, *J* = 8.67 Hz, arom.), 7.55 (2H, d, *J* = 8.67 Hz, arom.), 7.46 (1H, d, *J* = 1.51 Hz, arom.), 7.33–7.37 (3H, m, arom.). ^13^C NMR (CDCl_3_, 75 MHz) δ 176.6, 163.2, 159.9, 159.2, 140.3, 139.9, 135.2, 131.5, 130.8, 129.6, 129.4, 124.4, 116.1, 115.9, 114.9, 114.7.

#### 3.7.41. 7-Chloro-3-(4-chlorophenyl)-2-mercaptoquinazolin-4(3*H*)-one (**9h**)

White powder; mp = 302–303 °C (296 °C [53]); *R*_f_ = 0.88; ^1^H NMR (DMSO-*d*_6_, 300 MHz) δ 13.12 (1H, s, -SH), 7.93–7.96 (1H, d, *J* = 8.67 Hz, arom.), 7.67–7.69 (2H, d, *J* = 8.67 Hz, arom.), 7.45 (1H, d, *J* = 1.51 Hz, arom.), 7.33–7.39 (3H, m, arom.). ^13^C NMR (CDCl_3_, 75 MHz) δ 176.4, 159.1, 140.4, 137.9, 132.9, 130.9, 129.0, 124.4, 115.0.

#### 3.7.42. 3-(4-Bromophenyl)-7-chloro-2-mercaptoquinazolin-4(3*H*)-one (**9i**)

White powder; mp = 320–322 °C; *R*_f_ = 0.88; ^1^H NMR (DMSO-*d*_6_, 300 MHz) δ 13.12 (1H, s, -SH), 7.93–7.96 (1H, d, *J* = 8.67 Hz, arom.), 7.67–7.70 (2H, d, *J* = 8.67 Hz, arom.), 7.46 (1H, d, *J* = 1.51 Hz, arom.), 7.36–7.39 (1H, dd, *J* = 8.67, 1.88 Hz, arom.), 7.26–7.29 (2H, d, *J* = 8.67 Hz, arom.). ^13^C NMR (CDCl_3_, 75 MHz) δ 176.3, 159.1, 140.4, 139.9, 138.4, 131.9, 131.3, 129.6, 124.4, 121.4, 115.2, 114.9.

#### 3.7.43. 7-Chloro-2-mercapto-3-(3-methoxyphenyl)quinazolin-4(3*H*)-one (**9j**)

White powder; mp = 256–257 °C; *R*_f_ = 0.87; ^1^H NMR (DMSO-*d*_6_, 300 MHz) δ 13.06 (1H, s, -SH), 7.93–7.96 (1H, d, *J* = 8.67 Hz, arom.), 7.46 (1H, d, *J* = 1.88 Hz, arom.), 7.35–7.38 (2H, m, arom.), 6.99 (1H, dd, *J* = 8.29, 1.88 Hz, arom.), 6.90–6.92 (1H, t, *J* = 2.07, 2.07 Hz, arom.), 6.84–6.86 (2H, m, arom.), 3.75 (3H, s, -CH_3_). ^13^C NMR (CDCl_3_, 75 MHz) δ 176.4, 159.8, 159.0, 140.4, 140.0, 139.9, 129.6, 124.3, 121.1, 115.2, 115.0, 114.9, 113.6, 55.0.

#### 3.7.44. 7-Chloro-3-(3-chlorophenyl)-2-mercaptoquinazolin-4(3*H*)-one (**9k**)

White powder; mp = 248–249 °C; *R*_f_ = 0.88; ^1^H NMR (DMSO-*d*_6_, 300 MHz) δ 13.13 (1H, s, -SH), 7.93–7.96 (1H, d, *J* = 8.67 Hz, arom.), 7.46–7.52 (5H, m, arom.), 7.37–7.40 (1H, d, arom.), 7.29–7.31 (1H, dd, *J* = 8.29, 1.88 Hz, arom.). ^13^C NMR (CDCl_3_, 75 MHz) δ 176.3, 159.1, 140.4, 139.9, 132.9, 130.5, 129.6, 129.1, 128.3, 128.0, 127.8, 124.4, 115.2, 115.1, 99.5.

#### 3.7.45. 6,8-Dichloro-2-mercapto-3-methylquinazolin-4(3*H*)-one (**10a**)

Pale yellow powder; mp = 246 °C; *R*_f_ = 0.91; ^1^H NMR (DMSO-*d*_6_, 300 MHz) δ 11.86 (1H, s, -SH), 8.07 (1H, d, *J* = 2.93 Hz, arom.), 7.90 (1H, d, *J* = 2.93 Hz, arom.), 3.65 (3H, s, -CH_3_). ^13^C NMR (CDCl_3_, 75 MHz) δ 175.8, 157.9, 135.0, 134.6, 128.1, 125.6, 119.9, 118.2, 33.8.

#### 3.7.46. 6,8-Dichloro-3-ethyl-2-mercaptoquinazolin-4(3*H*)-one (**10b**)

White needles; mp = 184 °C; *R*_f_ = 0.89; ^1^H NMR (DMSO-*d*_6_, 300 MHz) δ 11.75 (1H, s, -SH), 8.05 (1H, d, *J* = 2.26 Hz, arom.), 7.88 (1H, d, *J* = 2.26 Hz, arom.), 4.43–4.45 (2H, q, *J* = 6.78 Hz, -CH2-), 1.21–1.25 (3H, t, *J* = 6.97 Hz, -CH_3_). ^13^C NMR (CDCl_3_, 75 MHz) δ 175.2, 157.3, 134.9, 134.7, 128.2, 125.6, 125.4, 119.8, 118.4, 41.8, 11.6.

#### 3.7.47. 3-Allyl-6,8-dichloro-2-mercaptoquinazolin-4(3*H*)-one (**10c**)

White powder; mp = 179 °C; *R*_f_ = 0.88; ^1^H NMR (DMSO-*d*_6_, 300 MHz) δ 11.84 (1H, s, -SH), 8.06 (1H, d, *J* = 2.20 Hz, arom.), 7.89 (1H, d, *J* = 2.93 Hz, arom.), 5.86–5.92 (1H, ddt, *J* = 16.96, 10.36, 5.41 Hz, -CH=), 5.17–5.20 (2H, m, -CH_2_-), 5.03 (2H, m, =CH_2_). ^13^C NMR (CDCl_3_, 75 MHz) δ 175.4, 157.3, 135.1, 134.8, 131.2, 128.2, 125.6, 119.9, 118.2, 117.3, 48.1.

#### 3.7.48. 6,8-Dichloro-2-mercapto-3-phenylquinazolin-4(3*H*)-one (**10d**)

White powder; mp = 283–285 °C; *R*_f_ = 0.89; ^1^H NMR (DMSO-*d*_6_, 300 MHz) δ 11.98 (1H, s, -SH), 8.11 (1H, d, *J* = 2.26 Hz, arom.), 7.89 (1H, d, *J* = 2.26 Hz, arom.), 7.42–7.52 (3H, m, arom.), 7.27–7.29 (2H, d, *J* = 7.16 Hz, arom.). ^13^C NMR (CDCl_3_, 75 MHz) δ 176.6, 158.1, 139.1, 135.6, 134.8, 129.0, 128.7, 125.7, 119.9.

#### 3.7.49. 3-Benzyl-6,8-dichloro-2-mercaptoquinazolin-4(3*H*)-one (**10e**)

White powder; mp = 206–208 °C; *R*_f_ = 0.93; ^1^H NMR (DMSO-*d*_6_, 300 MHz) δ 11.94 (1H, s, -SH), 8.07 (1H, d, *J* = 2.26 Hz, arom.), 7.89 (1H, m, arom.), 7.23–7.34 (5H, m, arom.), 5.67 (2H, s, *J* = 7.16 Hz, -CH_2_-). ^13^C NMR (CDCl_3_, 75 MHz) δ 175.9, 157.8, 135.9, 134.9, 128.3, 127.1, 125.7, 120.0, 118.3, 49.2.

#### 3.7.50. 6,8-Dichloro-2-mercapto-3-(*p*-tolyl)quinazolin-4(3*H*)-one (**10f**)

White powder; mp = 244 °C; *R*_f_ = 0.81; ^1^H NMR (DMSO-*d*_6_, 300 MHz) δ 11.93 (1H, s, -SH), 8.10 (1H, d, *J* = 2.26 Hz, arom.), 7.87 (1H, d, *J* = 2.64 Hz, arom.), 7.27–7.30 (2H, d, *J* = 8.29 Hz, arom.), 7.13–7.16 (2H, d, *J* = 8.29 Hz, -CH_2_-), 2.37 (3H, s, -CH_3_). ^13^C NMR (CDCl_3_, 75 MHz) δ 176.7, 158.1, 137.6, 136.5, 135.5, 134.8, 129.6, 128.4, 128.1, 125.7, 119.9, 119.3, 20.8.

#### 3.7.51. 6,8-Dichloro-3-(4-fluorophenyl)-2-mercaptoquinazolin-4(3*H*)-one (**10g**)

White powder; mp = 268–269 °C; *R*_f_ = 0.88; ^1^H NMR (DMSO-*d*_6_, 300 MHz) δ 12.04 (1H, s, -SH), 8.11 (1H, d, *J* = 2.26 Hz, arom.), 7.88 (1H, d, *J* = 2.26 Hz, arom.), 7.33–7.35 (4H, m, arom.). ^13^C NMR (CDCl_3_, 75 MHz) δ 176.7, 163.2, 160.0, 158.2, 135.5, 134.9, 130.9, 128.1, 125.7, 119.9, 119.3, 116.1, 115.8.

#### 3.7.52. 6,8-Dichloro-3-(4-chlorophenyl)-2-mercaptoquinazolin-4(3*H*)-one (**10h**)

White powder; mp = 259–260 °C; *R*_f_ = 0.89; ^1^H NMR (DMSO-*d*_6_, 300 MHz) δ 12.07 (1H, s, -SH), 8.11 (1H, d, *J* = 2.93 Hz, arom.), 7.88 (1H, d, *J* = 2.20 Hz, arom.), 7.56 (2H, m, arom.), 7.33–7.34 (2H, m, arom.). ^13^C NMR (CDCl3, 75 MHz) δ 176.5, 158.0, 138.0, 135.6, 134.9, 132.9, 130.8, 129.1, 128.9, 125.7, 119.9, 119.3.

#### 3.7.53. 3-(4-Bromophenyl)-6,8-dichloro-2-mercaptoquinazolin-4(3H)-one (**10i**)

White powder; mp = 280–282 °C; *R*_f_ = 0.80; ^1^H NMR (DMSO-*d*_6_, 300 MHz) δ 12.07 (1H, s, -SH), 8.11 (1H, d, *J* = 2.20 Hz, arom.), 7.88 (1H, d, *J* = 2.20 Hz, arom.), 7.68–7.70 (2H, m, arom.), 7.26–7.28 (2H, m, arom.). ^13^C NMR (CDCl_3_, 75 MHz) δ 176.4, 158.0, 138.5, 135.5, 134.9, 132.1, 131.9, 131.2, 131.1, 128.1, 125.7, 121.5, 119.9, 119.3.

#### 3.7.54. 6,8-Dichloro-2-mercapto-3-(3-methoxyphenyl)quinazolin-4(3*H*)-one (**10j**)

White powder; mp = 219–220 °C; *R*_f_ = 0.85; ^1^H NMR (DMSO-*d*_6_, 300 MHz) δ 11.97 (1H, s, -SH), 8.11 (1H, d, *J* = 2.26 Hz, arom.), 7.88 (1H, d, *J* = 2.26 Hz, arom.), 7.39 (1H, m, arom.), 6.91 (1H, m, arom.), 6.87 (2H, m, arom.), 3.76 (3H, s, -OCH_3_). ^13^C NMR (CDCl_3_, 75 MHz) δ 176.4, 159.8, 157.9, 140.1, 135.5, 134.9, 129.7, 125.7, 121.0, 120.9, 119.3, 114.3, 113.8, 55.2.

## 4. Conclusions

DESs were found to be good solvents for catalyst-free synthesis of 2-mercaptoquinazolin-4(3*H*)-ones. Twenty different DESs were screened for their ability to serve as a solvent in the reaction of anthranilic acid and isothicyanates and the most effective solvent was proven to be choline chloride:urea (1:2). DESs were combined with microwave-promoted synthesis and ultrasound promoted synthesis, but the best method was found to be a simple stirring at 80 °C. Fifty-five different 2-mercaptoquinazolin-4(3*H*)-ones were synthesized using this simple method. The importance of using these methods towards conventional ones is manifested in a shorter reaction time, simplicity of operation, no use of catalysts and no need for product purification.

## Figures and Tables

**Table 1:** 2-Mercapto-3-phenylquinazolin-4(3*H*)-one yields obtained in different choline chloride-based DESs at 80 °C.

Entry	DES	Molar Ratio	Yield ** (%)
1	ChCl:Urea	1:2	63
2	ChCl:*N*-methylurea	1:3	59
3	ChCl:1,3-Dimethylurea	1:2	24
4	ChCl:Thiourea	1:2	20
5	ChCl:Acetamide	1:2	33
6	ChCl:Butane-1,4-diol	1:2	47
7	ChCl:Ethane-1,2-diol	1:2	24
8	ChCl:Glycerol	1:2	21
9	ChCl:Xylitol	1:1	14
10	ChCl:Sorbitol	1:1	10
11	ChCl:Glucose	2:1	*
12	ChCl:Fructose	2:1	*
13	ChCl:Citric acid	1:1	*
14	ChCl:Tartaric acid	1:1	*
15	ChCl:Oxalic acid	1:1	*
16	ChCl:Levulinic acid	1:2	*
17	ChCl:Lactic acid	1:2	*
18	ChCl:Malic acid	1:1	*
19	ChCl:Malonic acid	1:1	*
20	ChCl:Maleic acid	1:1	*

* The product was not obtained. ** The yields were calculated based on the anthranilic acid for the product after precipitation.

**Table 2:** A comparison of the substituted 2-mercaptoquinazolin-4(3*H*)-ones yields^1^ obtained with selected green methods with literature yields.

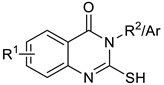							
Compounds	R ^1^	Ar/R ^2^	*Y_s_*_tirring_ (%) ^2^	*Y*_MW_ (%) ^3^	*Y*_US_ ^4^ (%)	*Y*_Lit._ (%)	*M*_p_ (°C)
**6a**	H	Me	42	39	17	46 [48]; 68 [33]; 60 [49]	264–265
**6b**	H	Et	40	18	36	72 [33]; 95 [50]	255
**6c**	H	Allyl	24	15	21	35 [33]	206–208
**6d**	H	Ph	63	22	34	35 [48]; 90 [33]; 92 [50]; 73 (82) [51]; 61 [49]; 75 (60) [52]	304–305
**6e**	H	Bn	62	41	64	50 [48]; 83 [53]	248–250
**6f**	H	4-MePh	27	47	38	94 [50]; 79 (78) [51]; 88 [54], 75 (62) [52]	312–313
**6g**	H	4-FPh	48	32	25	88 [3]; 88 [54]	336–337
**6h**	H	4-ClPh	55	49	41	75 [33]; 89 [50]; 94 [51]	331–332
**6i**	H	4-BrPh	66	24	58	84 [55]; 70 [52]	330–331
**6j**	H	3-OMePh	64	21	57	91 [54]; 65 (50) [52]	285
**6k**	H	3-ClPh	41	12	25	86 [54]	300–301
**7a**	6-I	Me	31	20	40	73 [33]	307–308
**7b**	6-I	Et	25	14	18	10 [33]	290–292
**7c**	6-I	Allyl	22	13	21	20 [33]	234–235
**7d**	6-I	Ph	32	16	22	91 [33]; 70 [52]	350–352
**7e**	6-I	Bn	45	10	28		352
**7f**	6-I	4-MePh	49	29	47		350–351
**7g**	6-I	4-FPh	43	22	18		349–350
**7h**	6-I	4-ClPh	39	31	38	75 [29]	337–339
**7i**	6-I	4-BrPh	49	30	51		355–357
**7j**	6-I	3-OMePh	53	25	58		314–315
**7k**	6-I	3-ClPh	50	25	44		313–315
**8a**	6-Br	Me	21	14	23	64 [49]	280–281
**8b**	6-Br	Et	25	13	17		243–244
**8c**	6-Br	Allyl	40	14	40		242–243
**8d**	6-Br	Ph	36	13	19	63 [49]; 75 [52]	351–353
**8e**	6-Br	Bn	58	27	60		244
**8f**	6-Br	4-MePh	76	31	41	60 [56]	341–342
**8g**	6-Br	4-FPh	57	19	28		354–355
**8h**	6-Br	4-ClPh	62	27	48		344–346
**8i**	6-Br	4-BrPh	65	33	56		349–350
**8j**	6-Br	3-OMePh	49	28	30		312–313
**8k**	6-Br	3-ClPh	57	18	33		305–307
**9a**	7-Cl	Me	58	19	18		327–328
**9b**	7-Cl	Et	22	15	21		265
**9c**	7-Cl	Allyl	47	13	26		248–249
**9d**	7-Cl	Ph	67	19	36	71 (90) [51]	313–314
**9e**	7-Cl	Bn	26	17	34		270–272
**9f**	7-Cl	4-MePh	42	13	24	73 [51]	307–309
**9g**	7-Cl	4-FPh	34	18	18		314–315
**9h**	7-Cl	4-ClPh	50	14	27	76 [53]	302–303
**9i**	7-Cl	4-BrPh	50	16	38		320–322
**9j**	7-Cl	3-OMePh	40	25	44		256–257
**9k**	7-Cl	3-ClPh	39	19	54		248–249
**10a**	6,8-(Cl)_2_	Me	36	15	41		246–247
**10b**	6,8-(Cl)_2_	Et	19	119	15		184
**10c**	6,8-(Cl)_2_	Allyl	21	23	30		179
**10d**	6,8-(Cl)_2_	Ph	20	18	19	65 [52]	283–285
**10e**	6,8-(Cl)_2_	Bn	51	14	27		206–208
**10f**	6,8-(Cl)_2_	4-MePh	25	27	40	70 [52]	244
**10g**	6,8-(Cl)_2_	4-FPh	33	22	24		268–269
**10h**	6,8-(Cl)_2_	4-ClPh	34	30	30		259–260
**10i**	6,8-(Cl)_2_	4-BrPh	60	37	48		280–282
**10j**	6,8-(Cl)_2_	3-OMePh	33	14	24		219–220
**10k**	6,8-(Cl)_2_	3-ClPh	38	24	43		214–216

^1^ The yields were calculated based on the anthranilic acid for the product after precipitation; ^2^ ChCl:urea (1:2), 80 °C, 1 h; ^3^ ChCl:urea (1:2), 1800 W, 80 °C, 1 h; ^4^ ChCl:urea (1:2), 50 W, 80 °C, 1 h.

**Table 3:** Recyclability of DES for the synthesis of **8f**.

Solvent	Yield of 8f (%)
ChCl:Urea	76
1st recycle	72
2nd recycle	77
3rd recycle	80
4th recycle	77

## Data Availability

Data is contained within the article and Supplementary Materials.

## Supplementary Materials

The following supporting information can be downloaded. ^1^H and ^13^C NMR spectra of the synthesized compounds.

## Sample Availability

Samples of all compounds are available from the authors.
