# Supplementary material for: Application of Deep Eutectic Solvents in the Synthesis of Substituted 2-Mercaptoquinazolin-4(3H)-Ones: A Comparison of Selected Green Chemistry Methods
Source: Molecules. 2022 Jan 16;27(2):558. doi: 10.3390/molecules27020558 (PMC8780518; doi:10.3390/molecules27020558)

Supplementary material

Application of Deep Eutectic Solvents in the Synthesis of Substituted 2-Mercaptoquinazolin-4(3*H*)-ones: A Comparison of Selected Green Chemistry Methods

Mario Komar [1], Tatjana Gazivoda Kraljević [2], Igor Jerković [3,*] and Maja Molnar [1,*]

1H NMR and 13C NMR specta of the synthesized compounds

2-mercapto-3-methylquinazolin-4(3*H*)-one (6a)

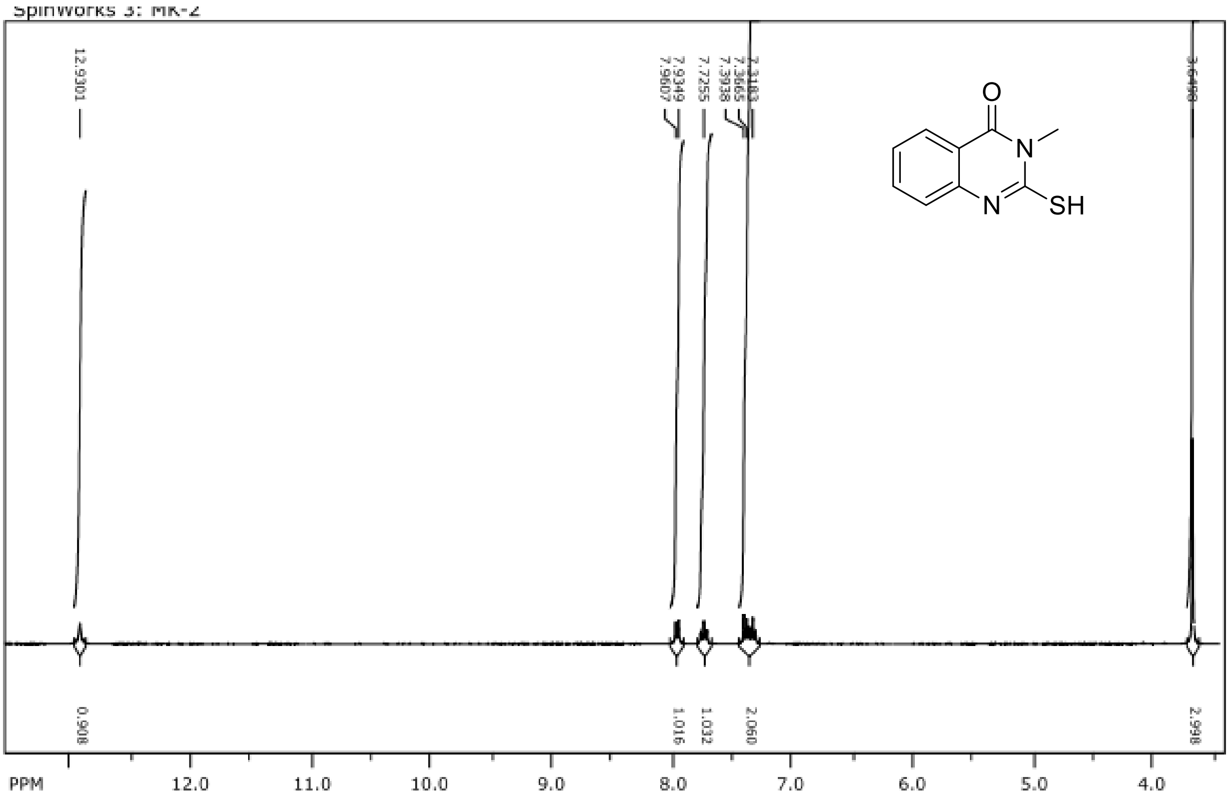

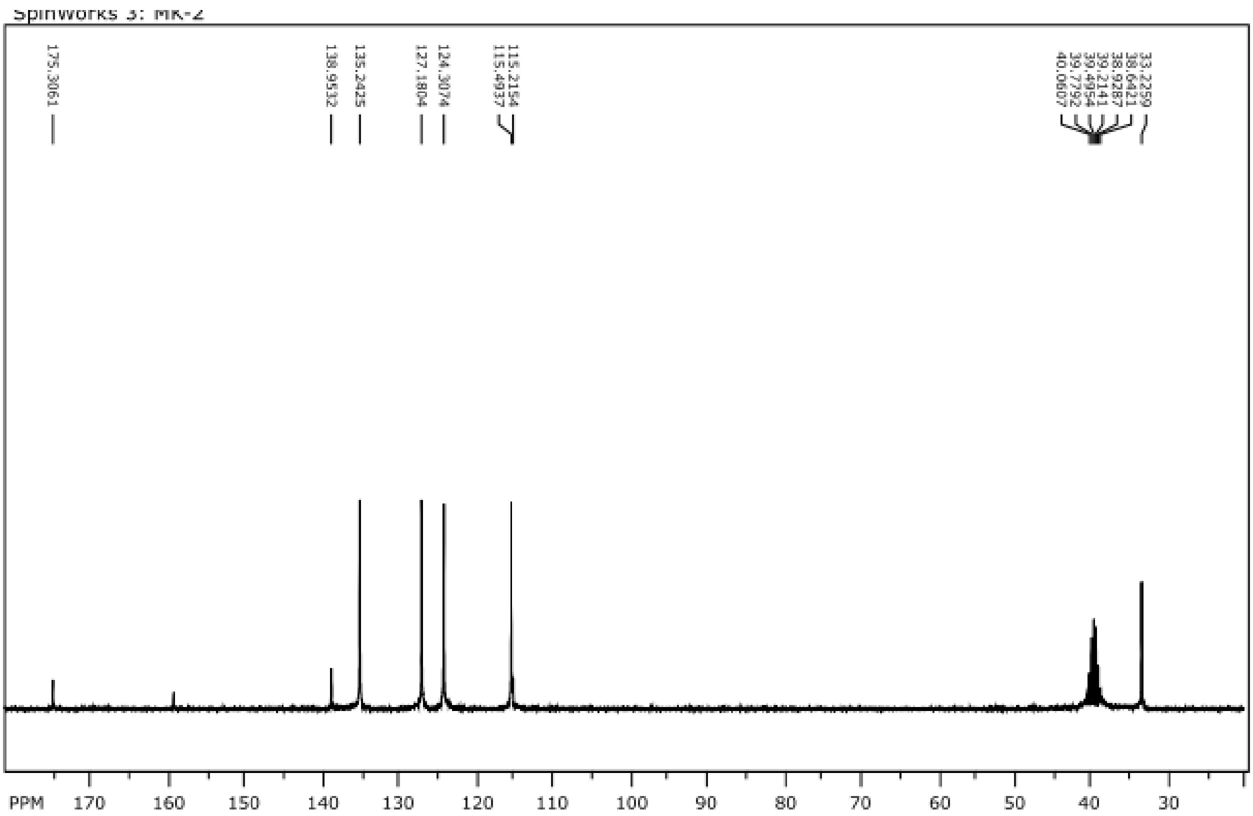

3-ethyl-2-mercaptoquinazolin-4(3*H*)-one (6b)

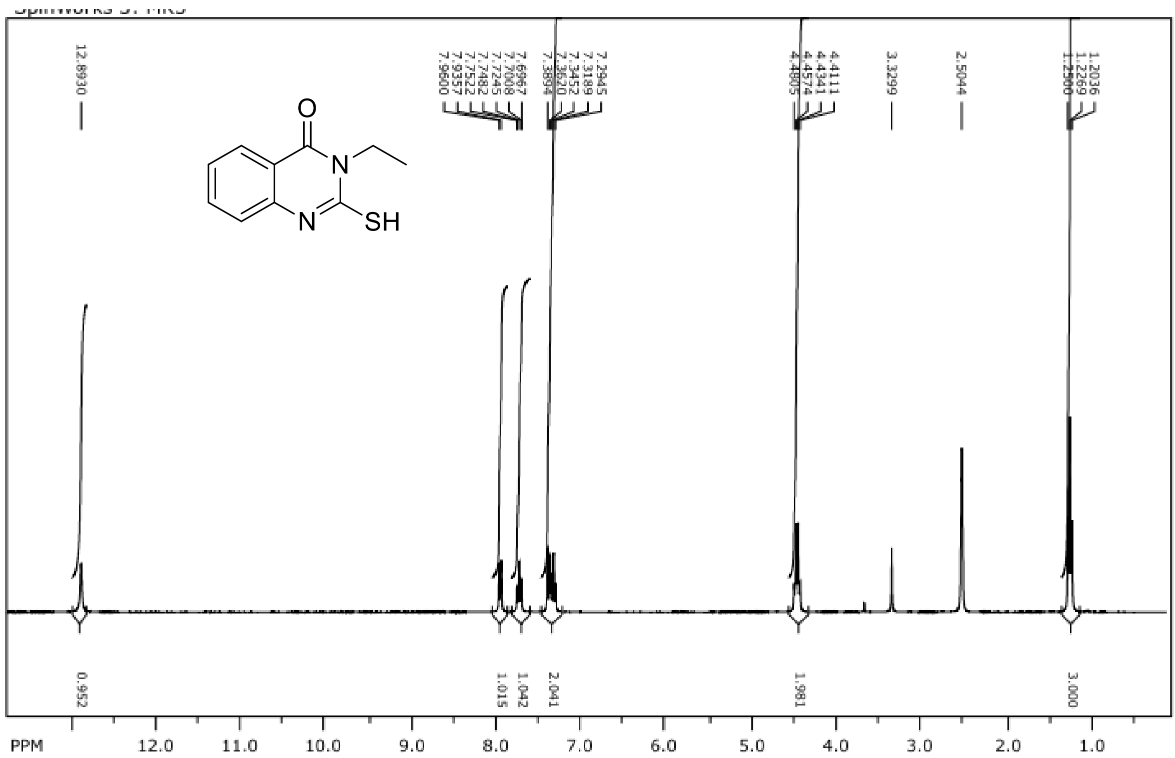

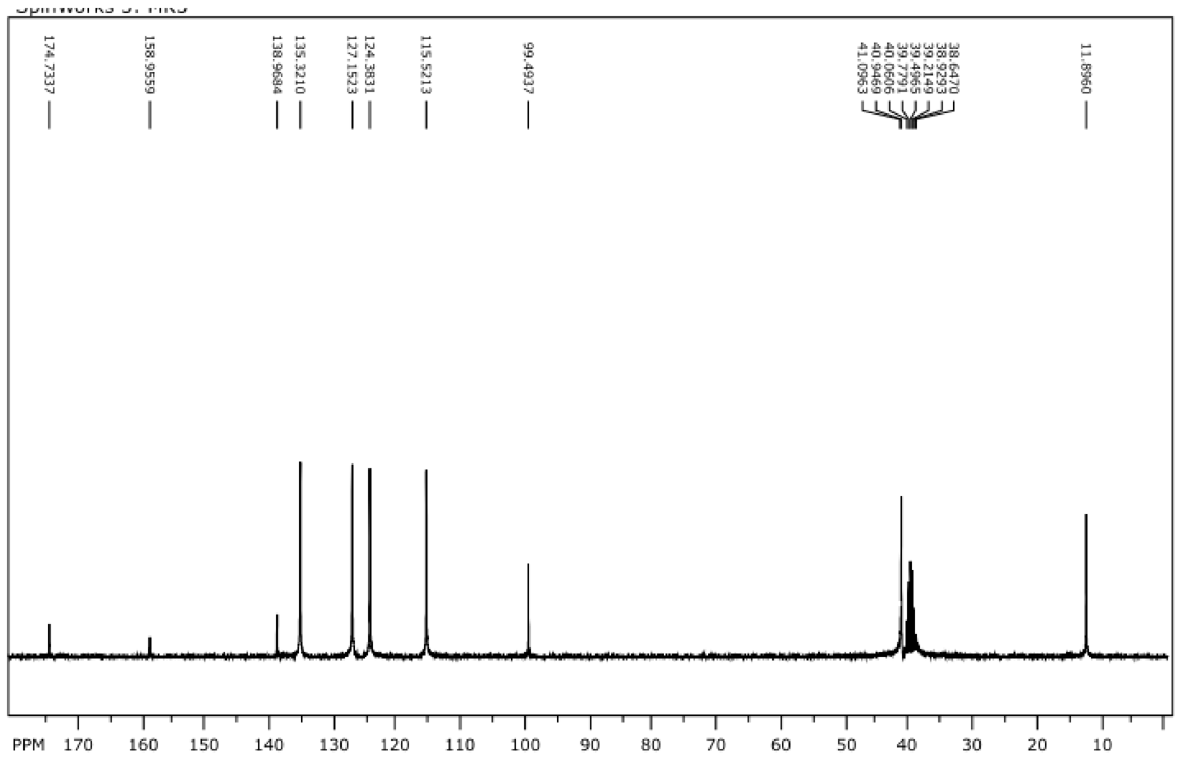

3-allyl-2-mercaptoquinazolin-4(3H)-one (6c)

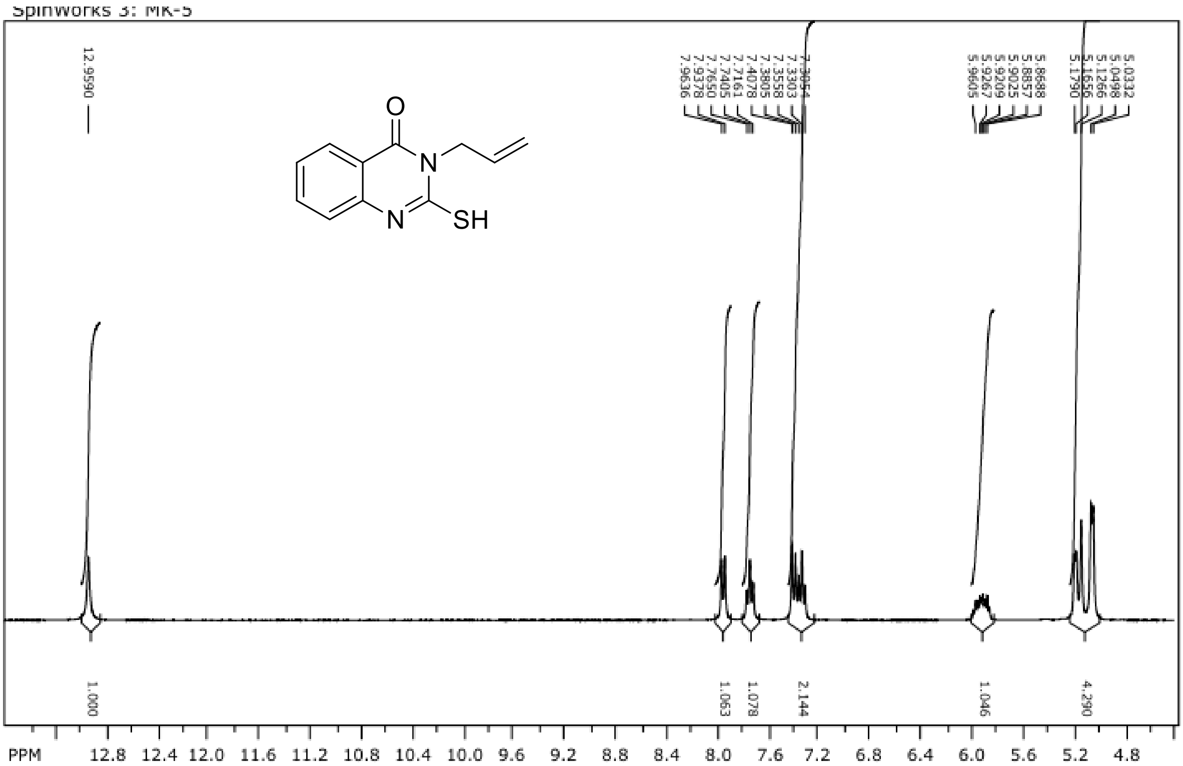

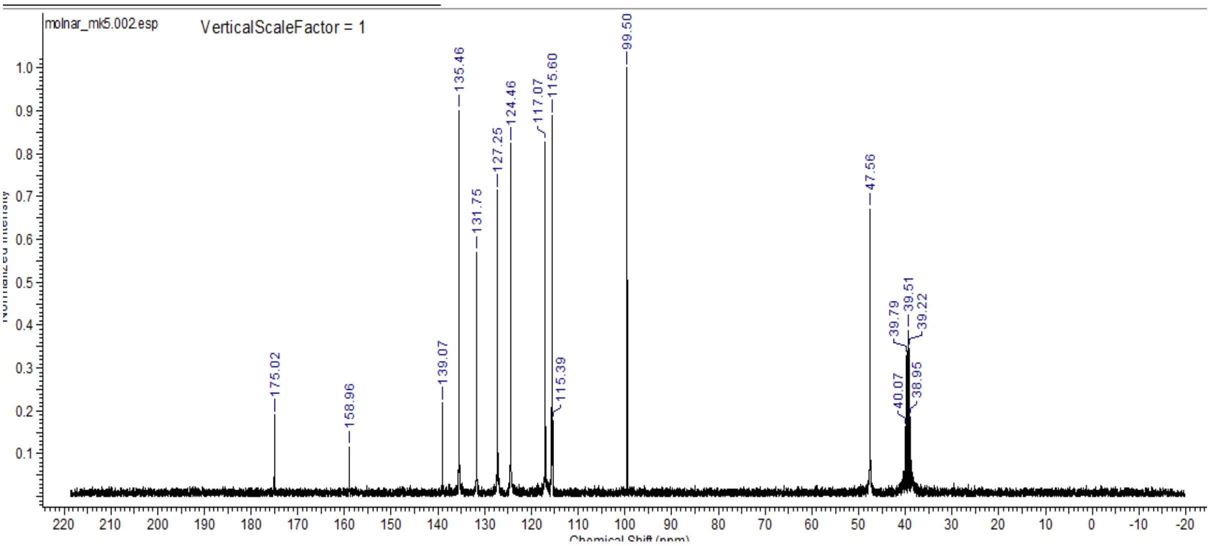

2-mercapto-3-phenylquinazolin-4(3H)-one (6d)

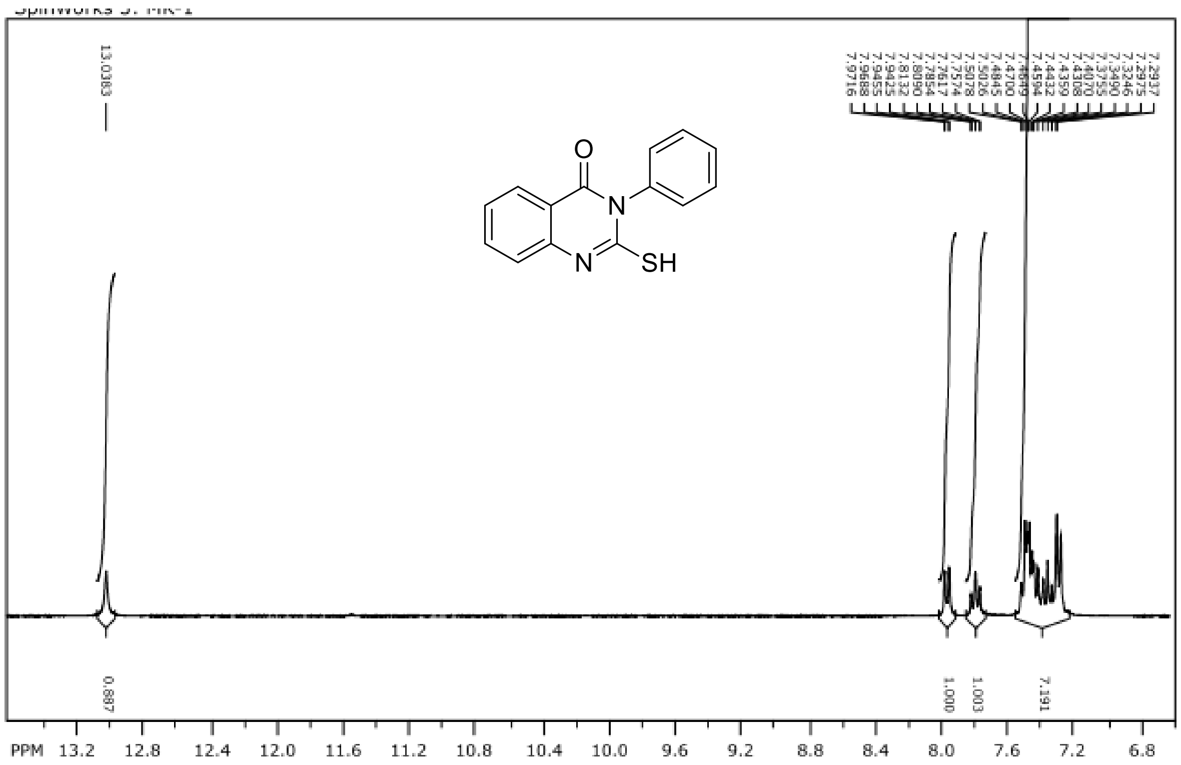

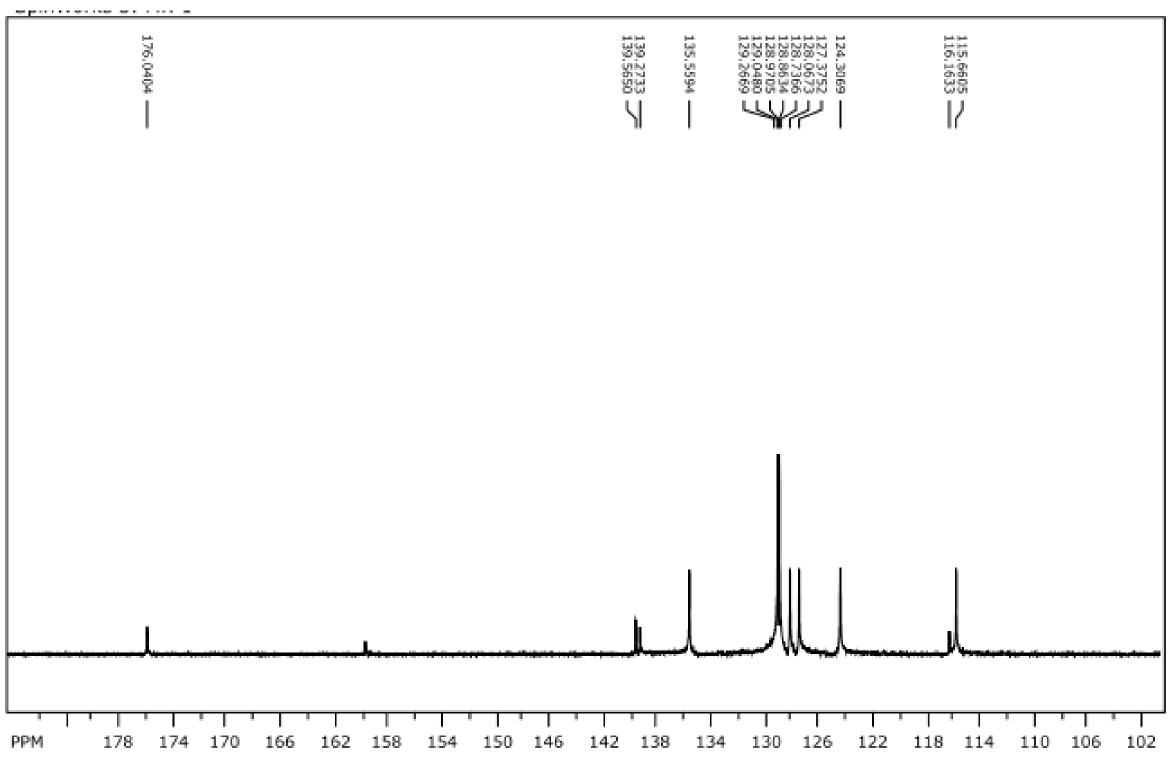

3-benzyl-2-mercaptoquinazolin-4(3H)-one (6e)

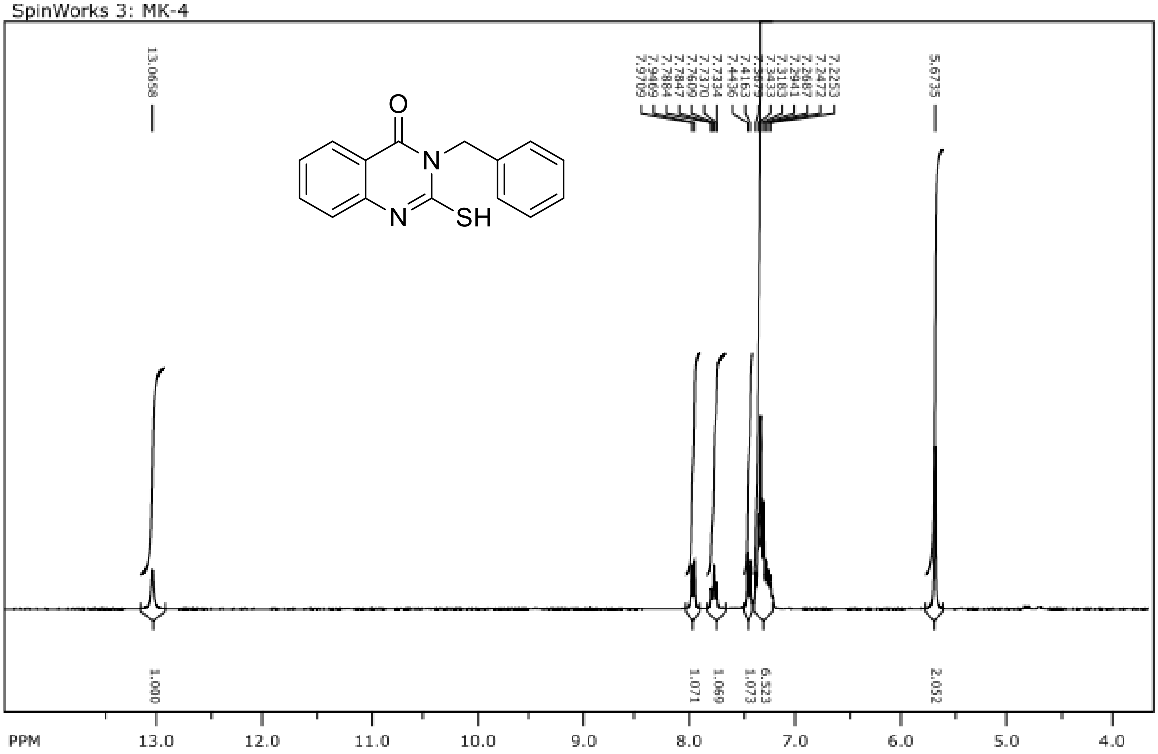

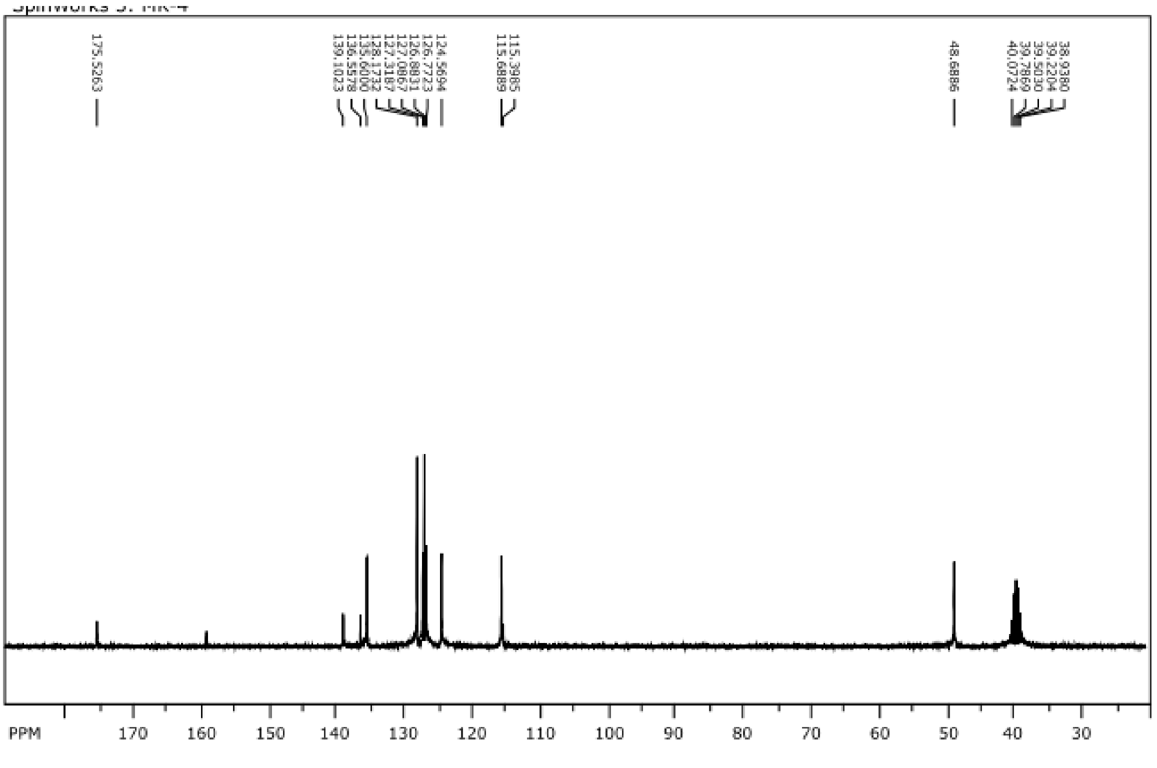

2-mercapto-3-(p-tolyl)quinazolin-4(3H)-one (6f)

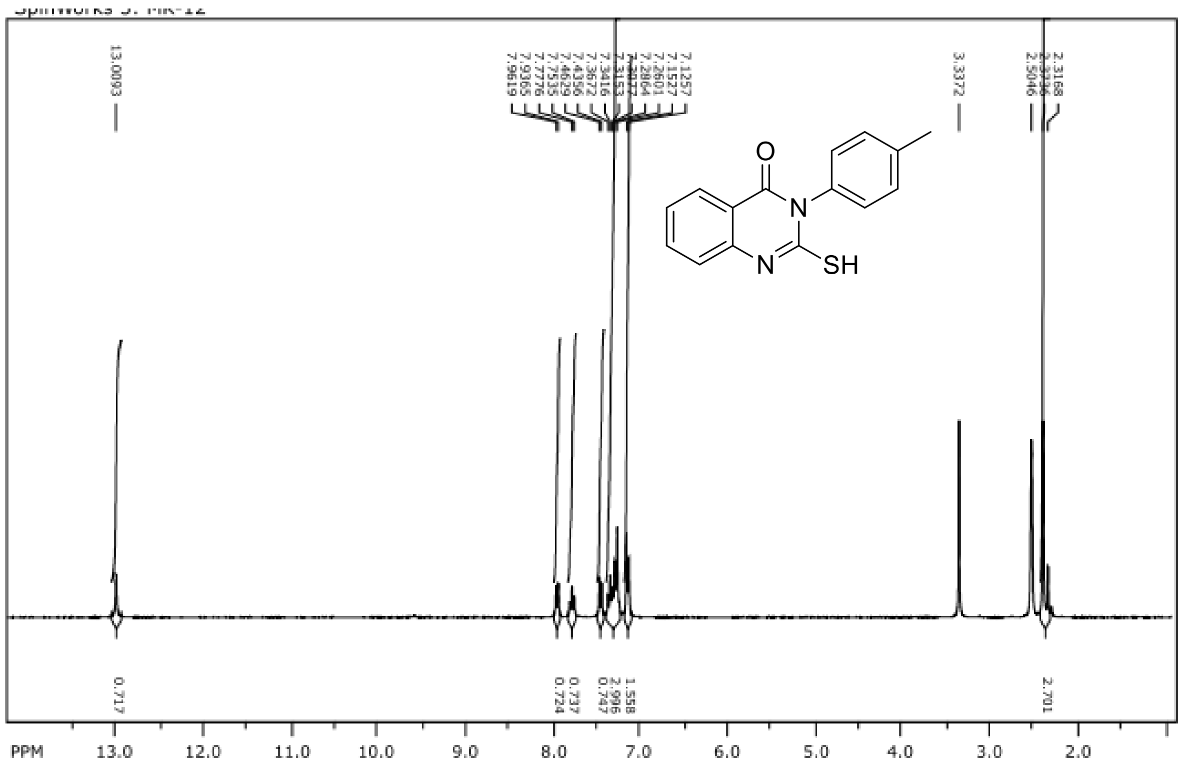

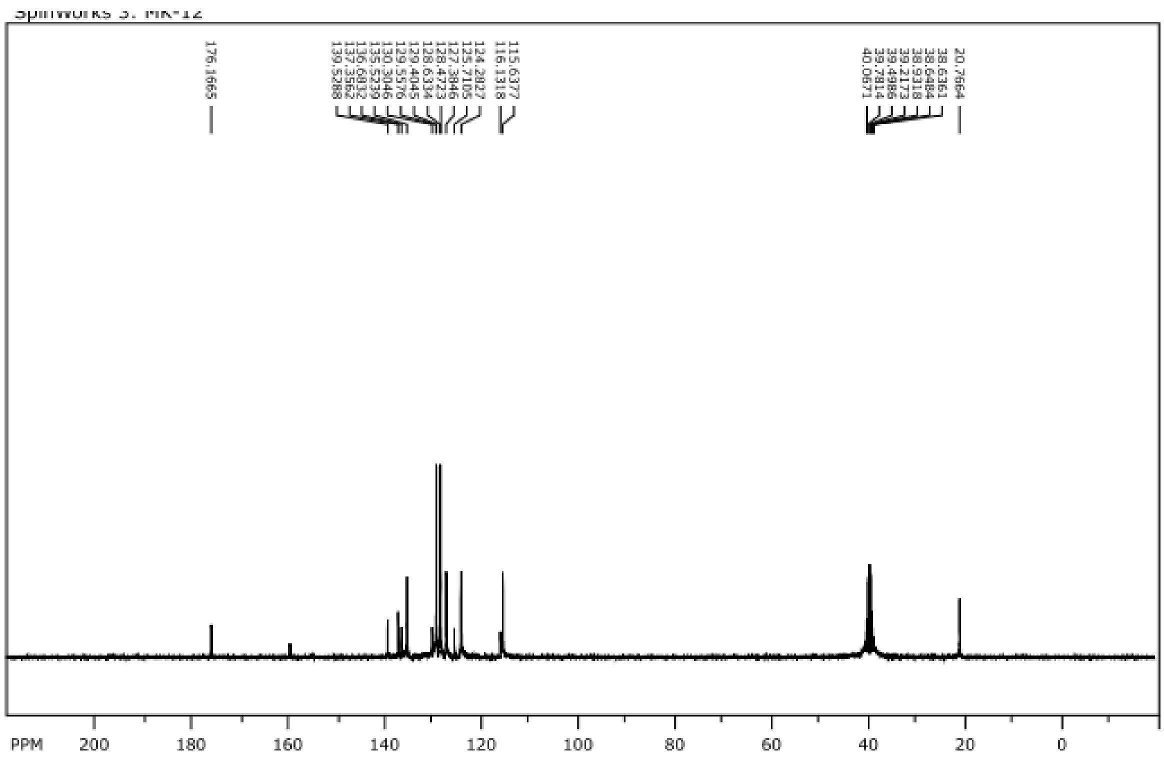

3-(4-fluorophenyl)-2-mercaptoquinazolin-4(3H)-one (6g)

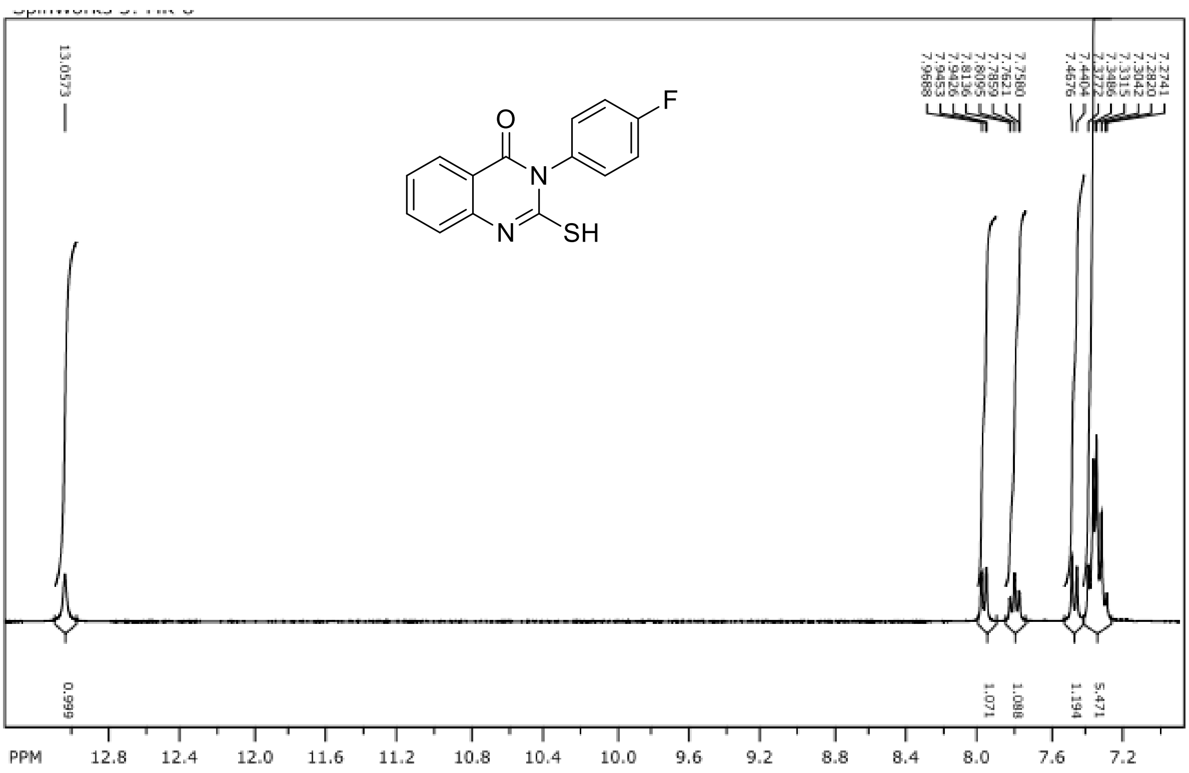

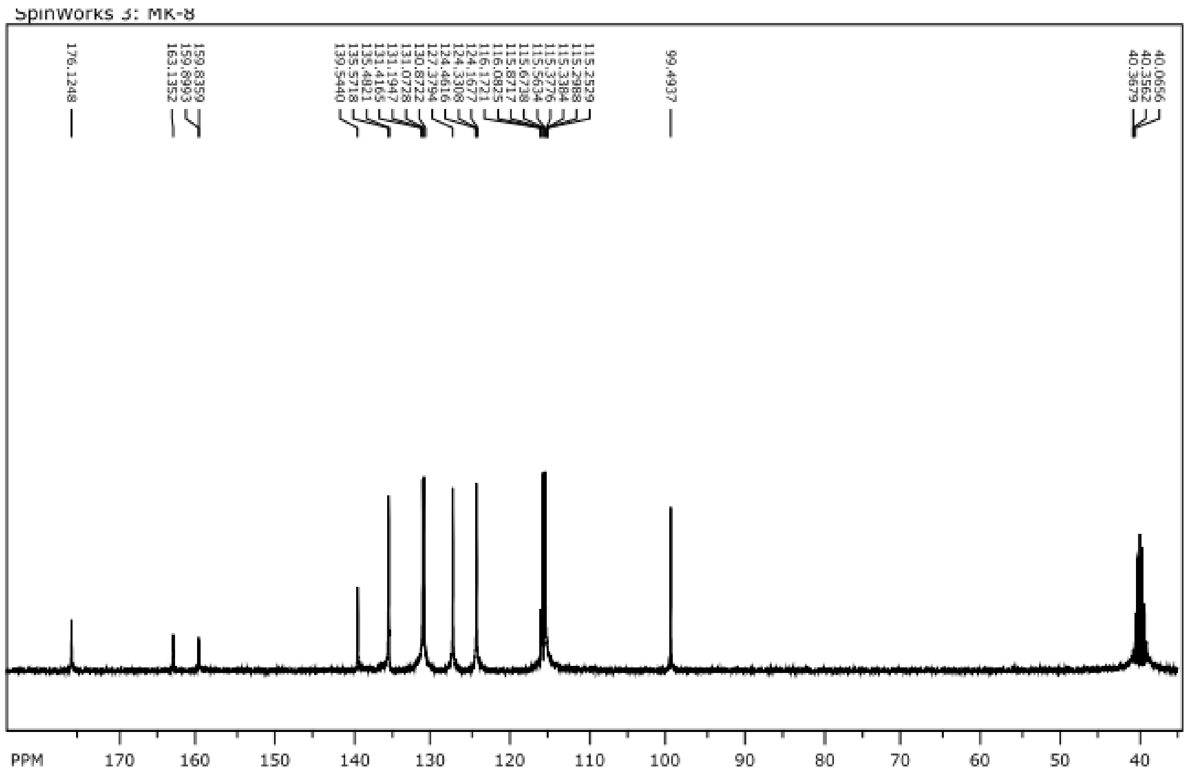

3-(4-chlorophenyl)-2-mercaptoquinazolin-4(3H)-one (6h)

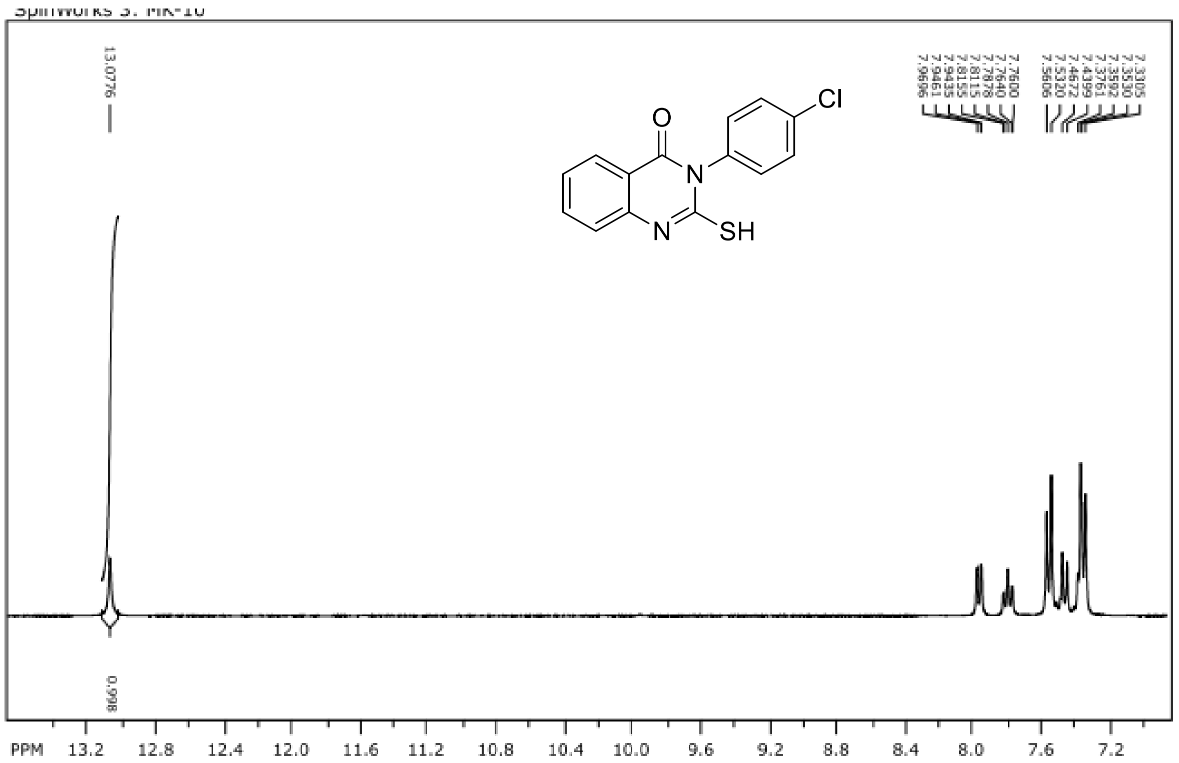

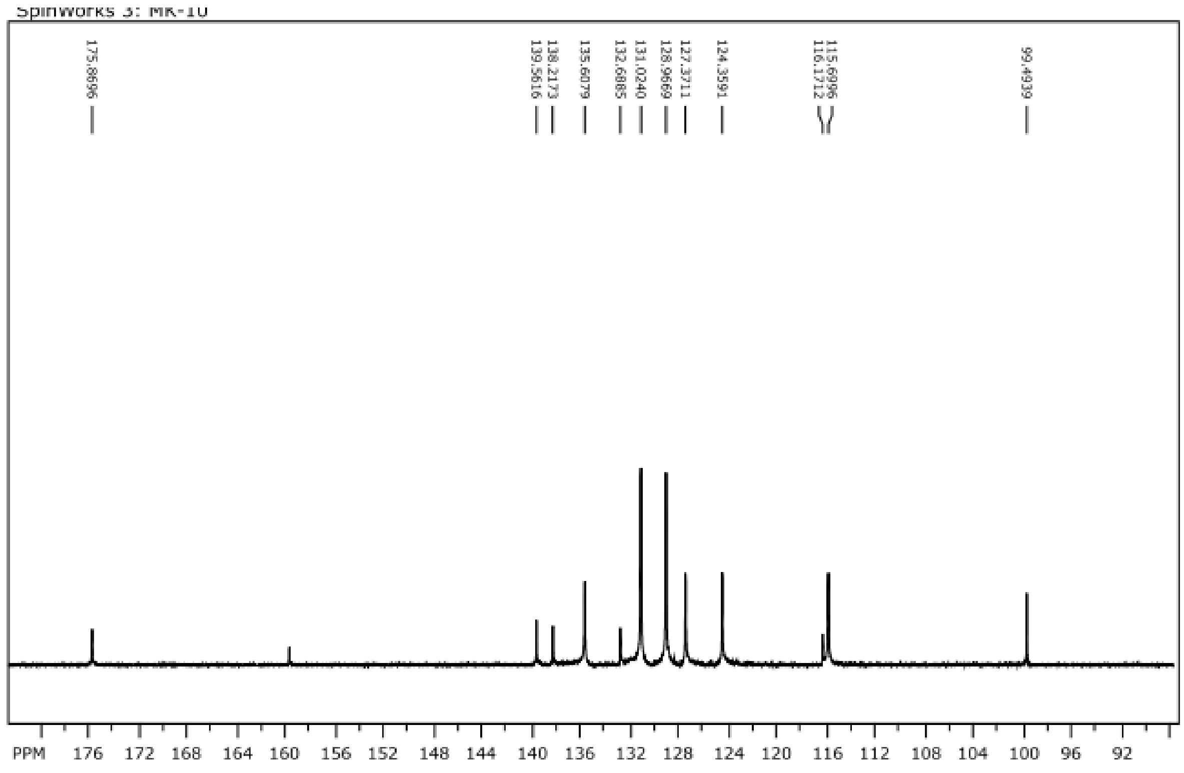

3-(4-bromophenyl)-2-mercaptoquinazolin-4(3H)-one (6i)

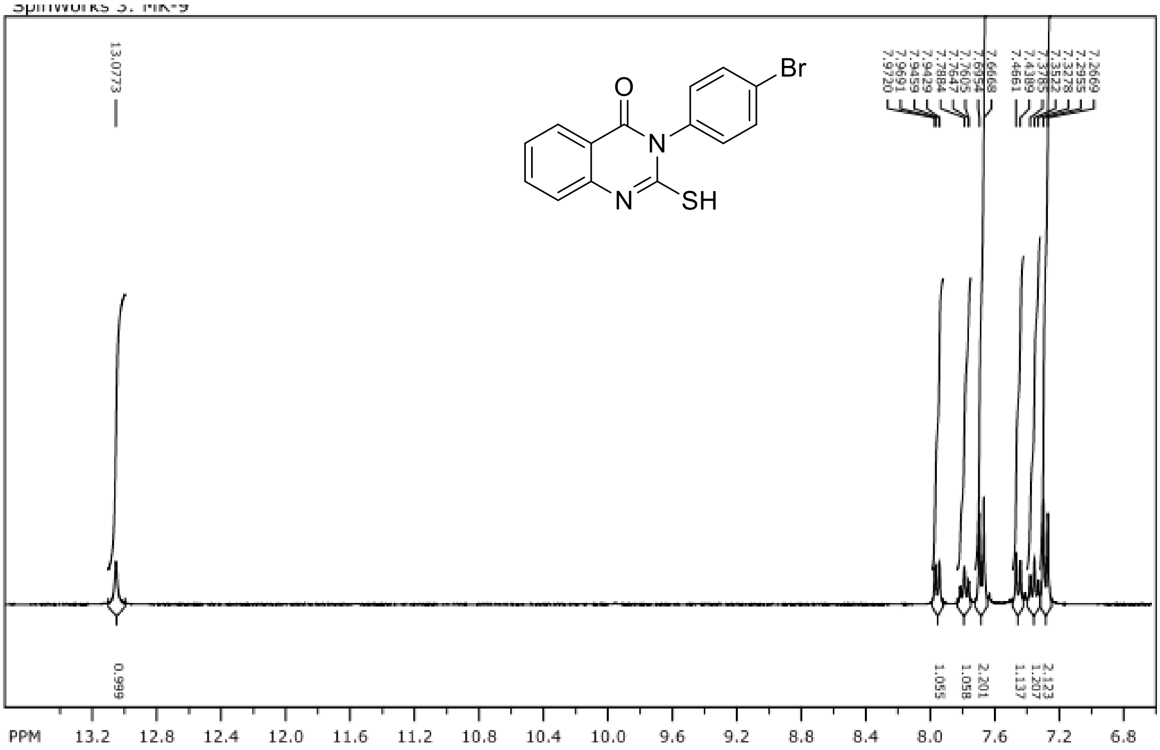

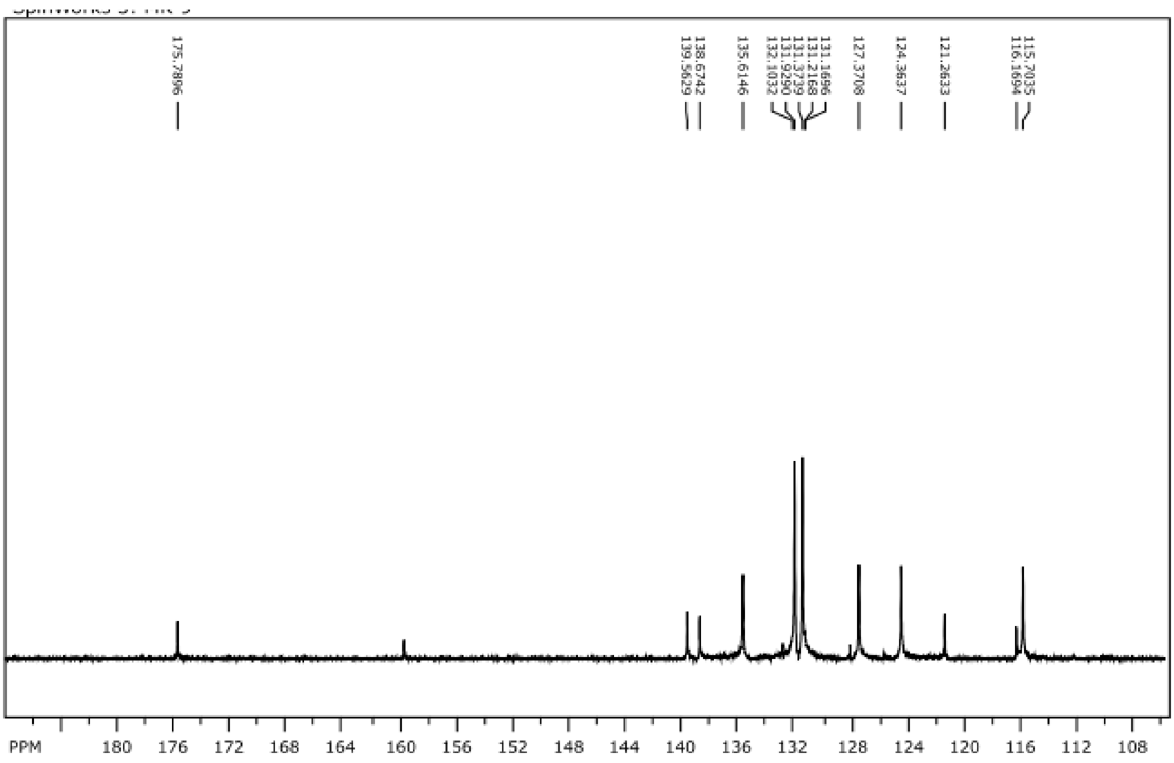

2-mercapto-3-(3-methoxyphenyl)quinazolin-4(3H)-one (6j)

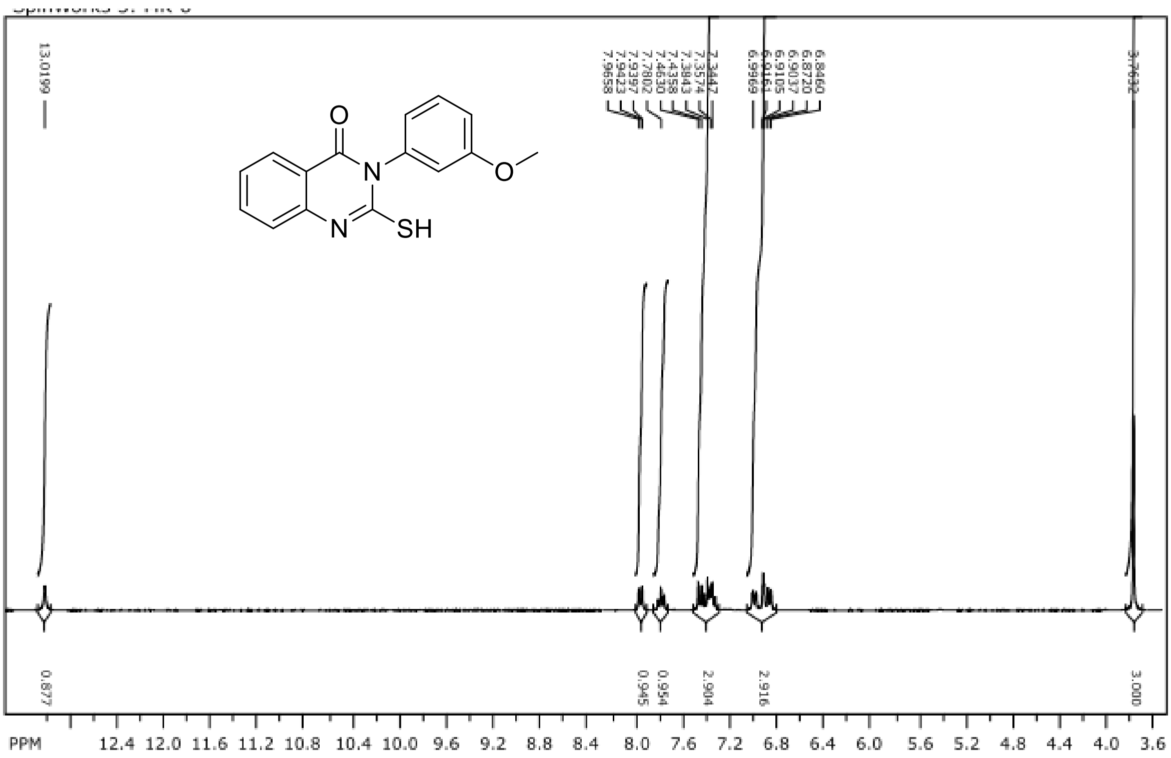

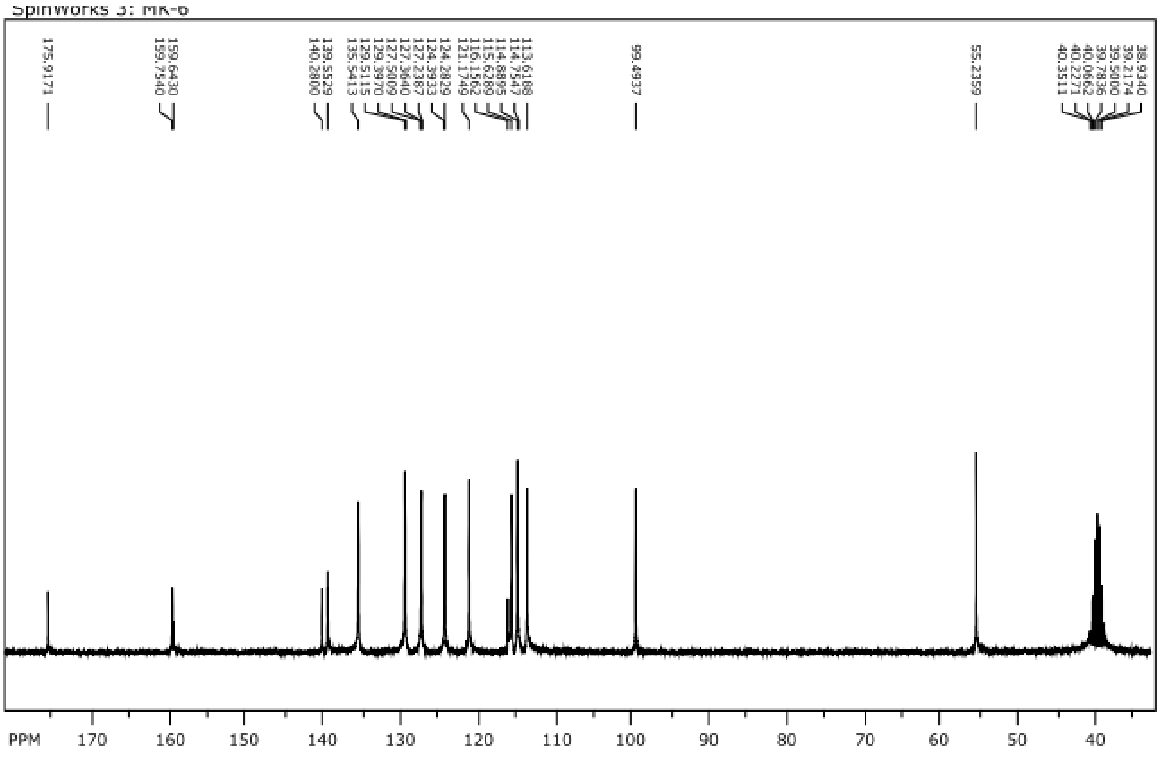

3-(3-chlorophenyl)-2-mercaptoquinazolin-4(3H)-one (6k)

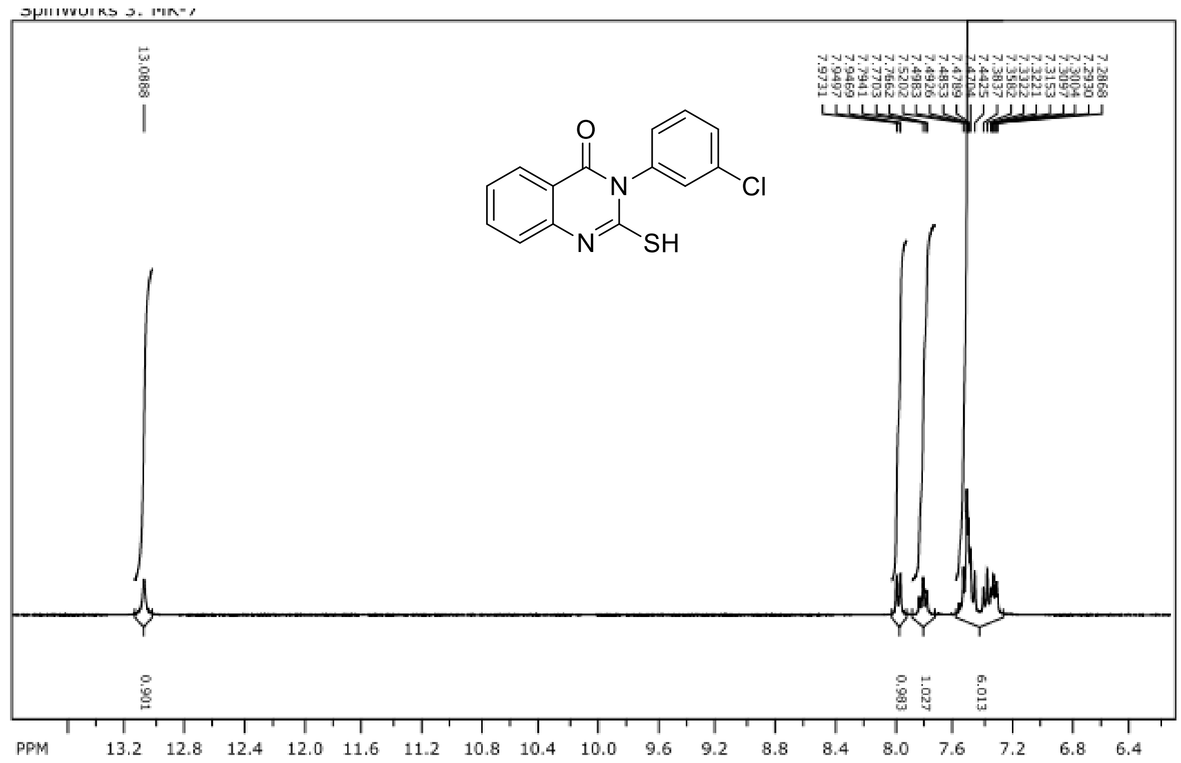

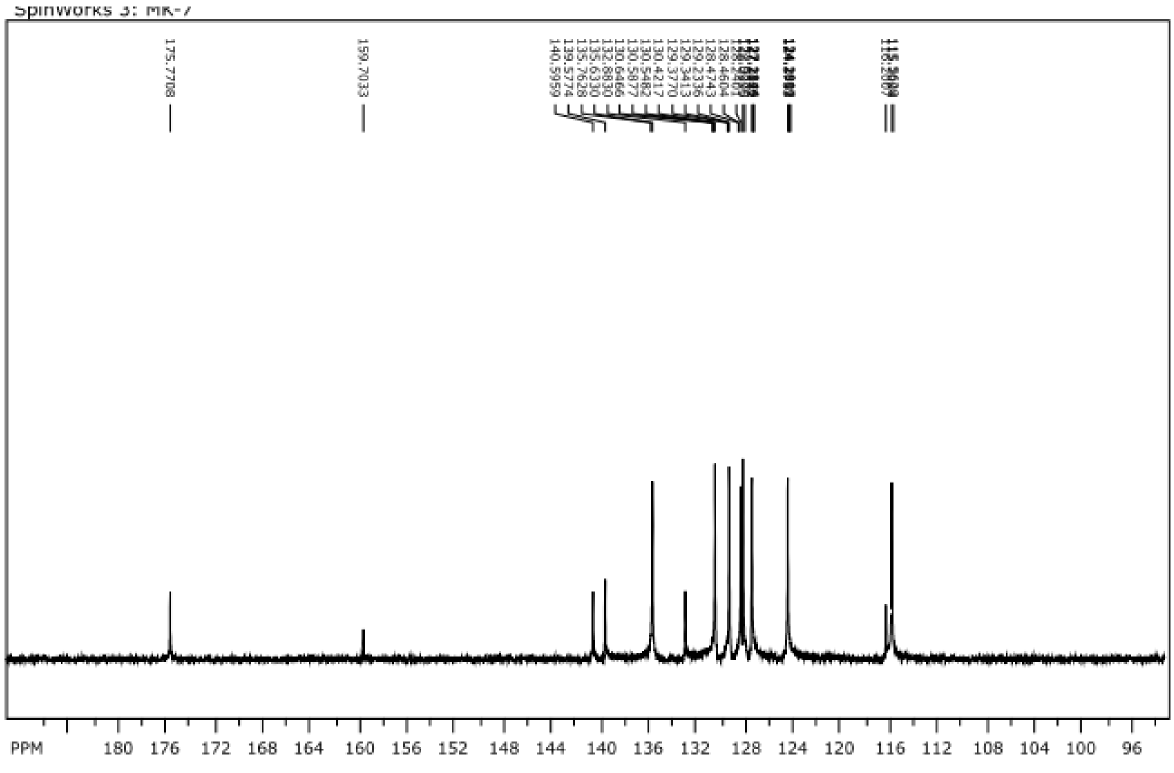

6-iodo-2-mercapto-3-methylquinazolin-4(3H)-one (7a)

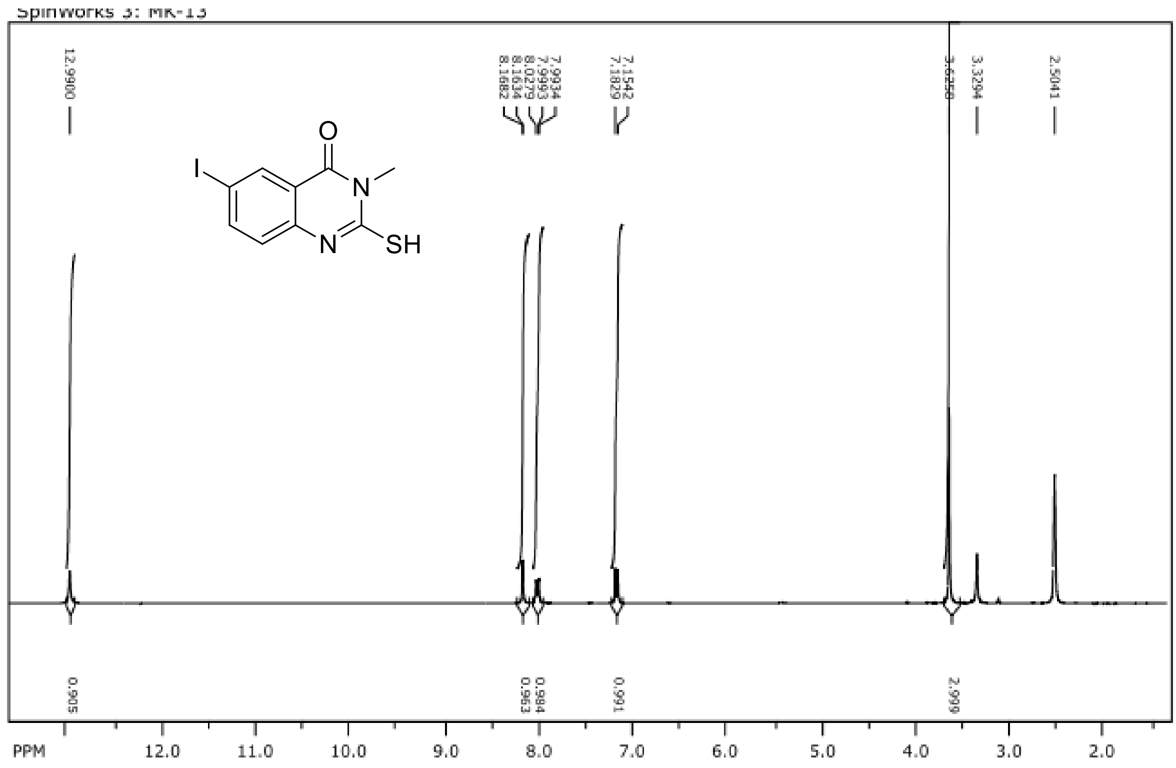

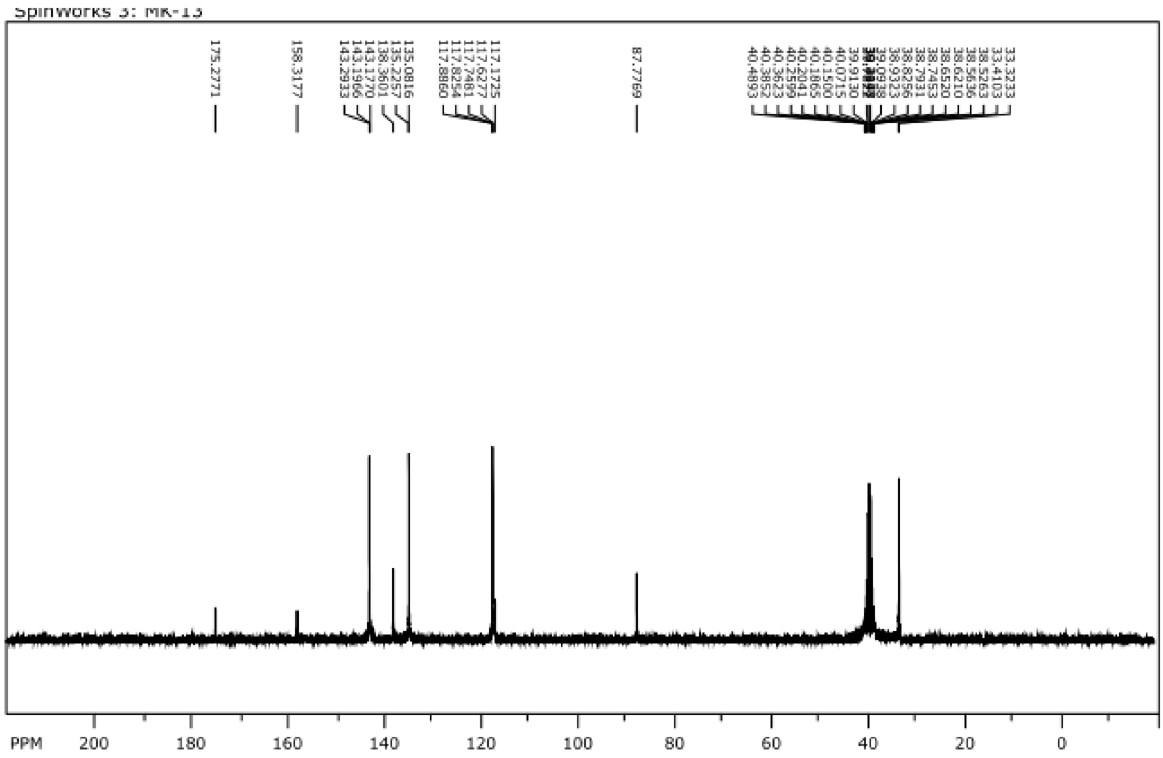

3-ethyl-6-iodo-2-mercaptoquinazolin-4(3H)-one (7b)

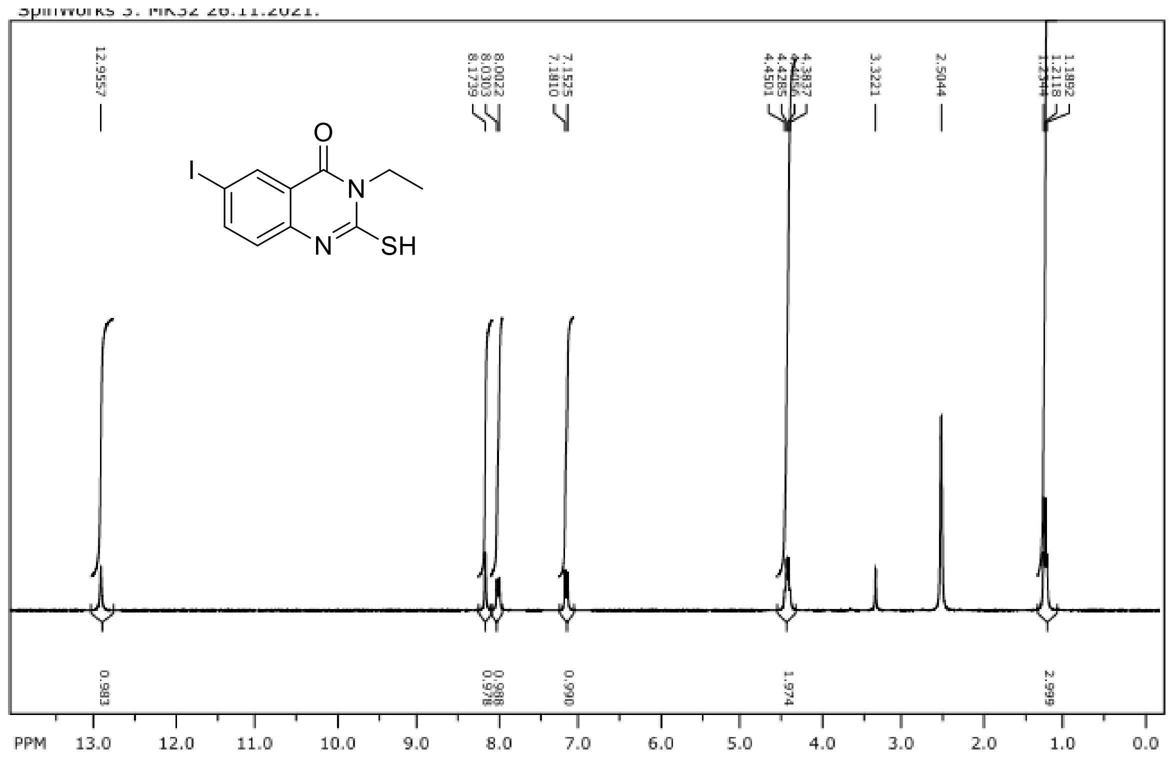

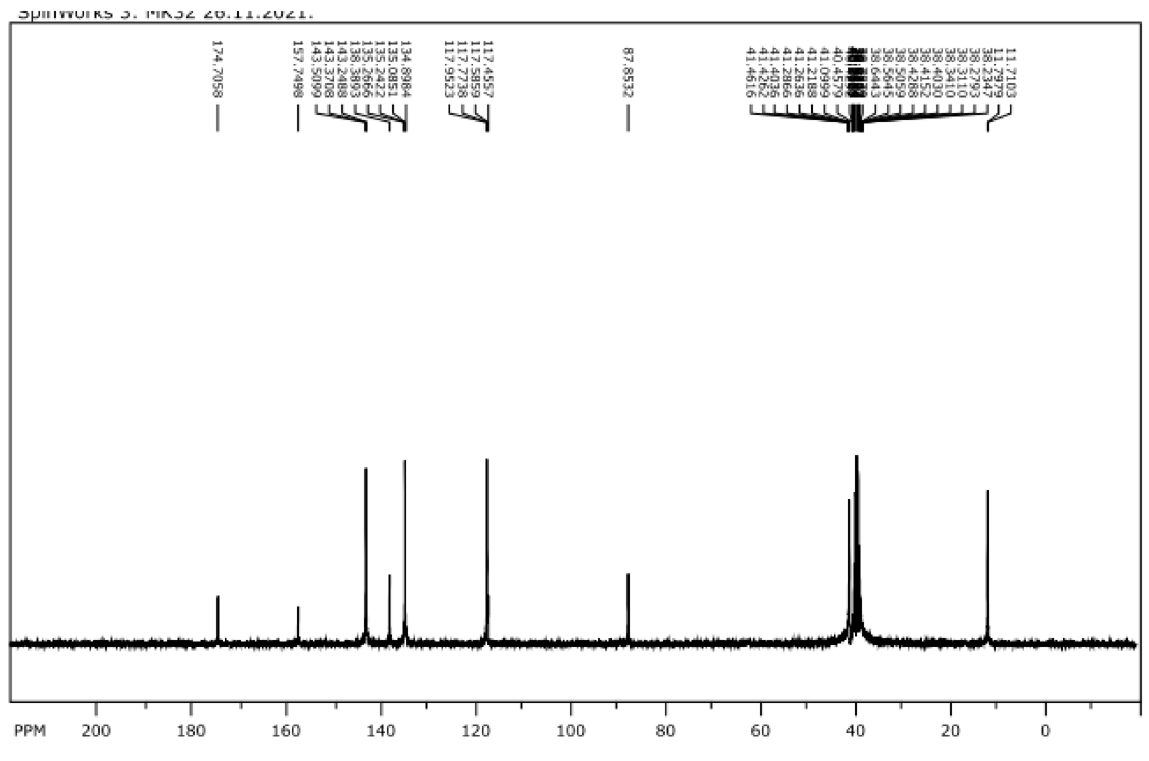

3-allyl-6-iodo-2-mercaptoquinazolin-4(3H)-one (7c)

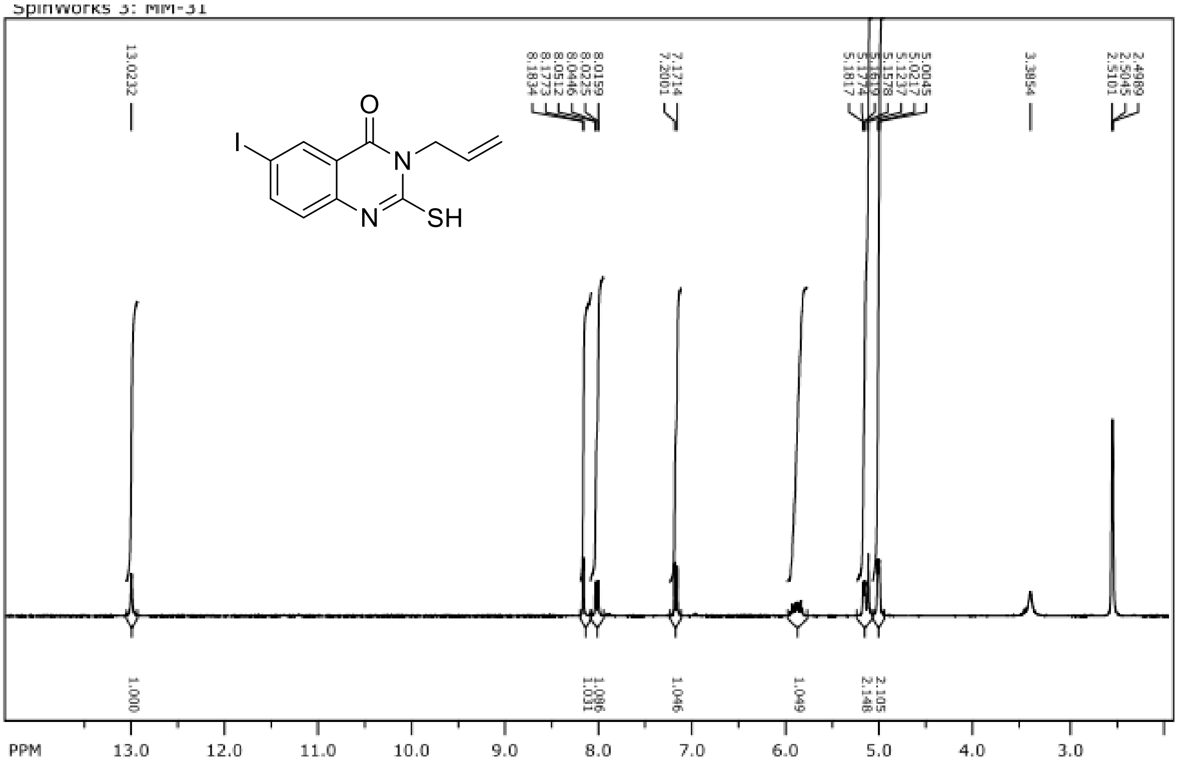

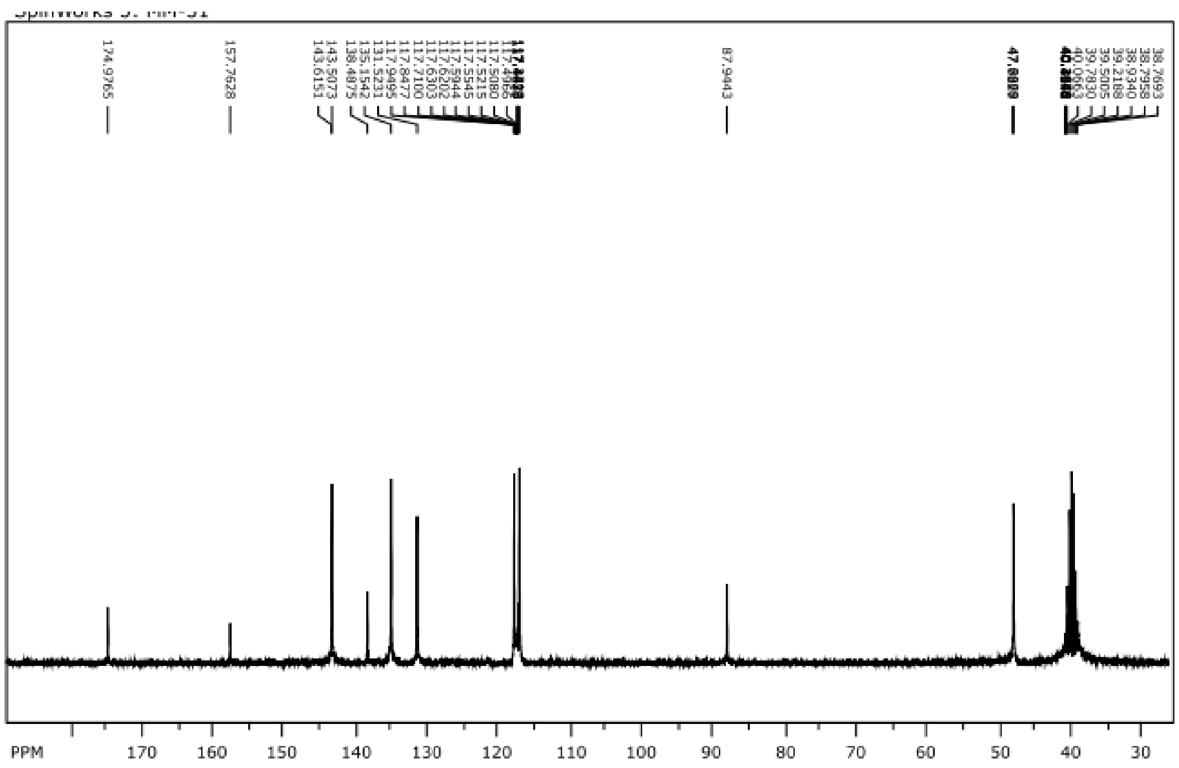

6-iodo-2-mercapto-3-phenylquinazolin-4(3H)-one (7d)

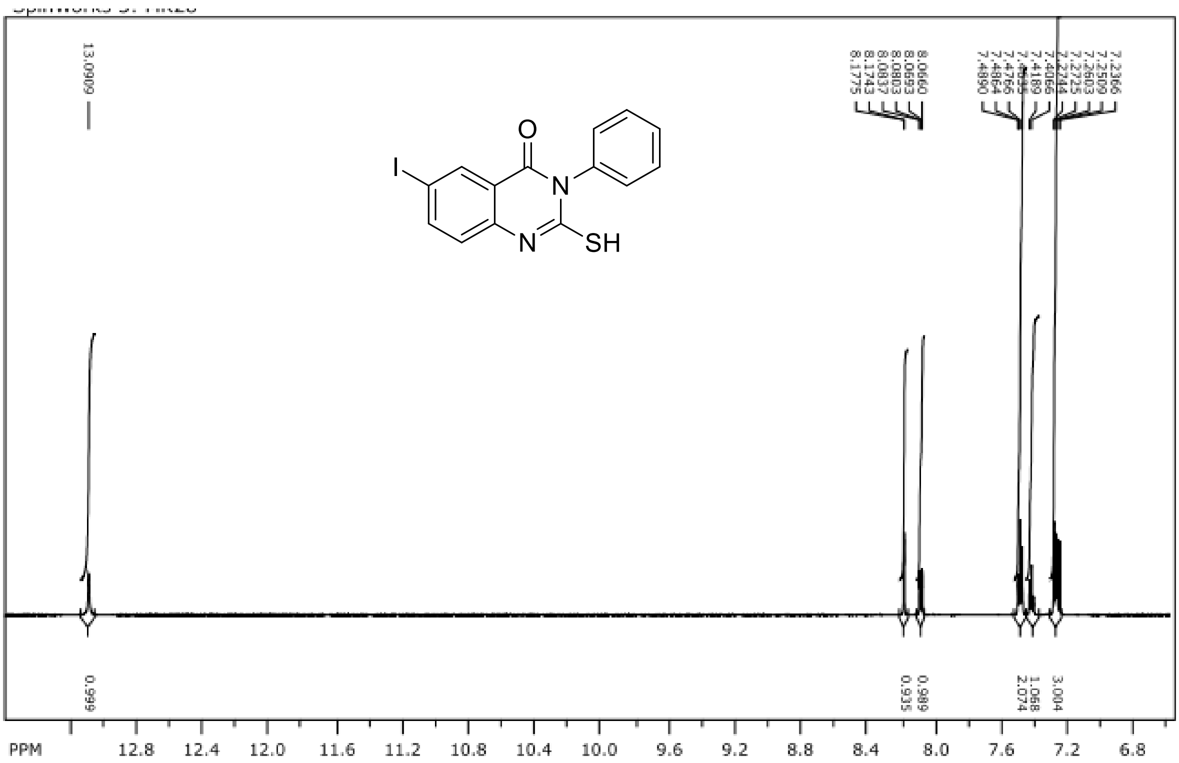

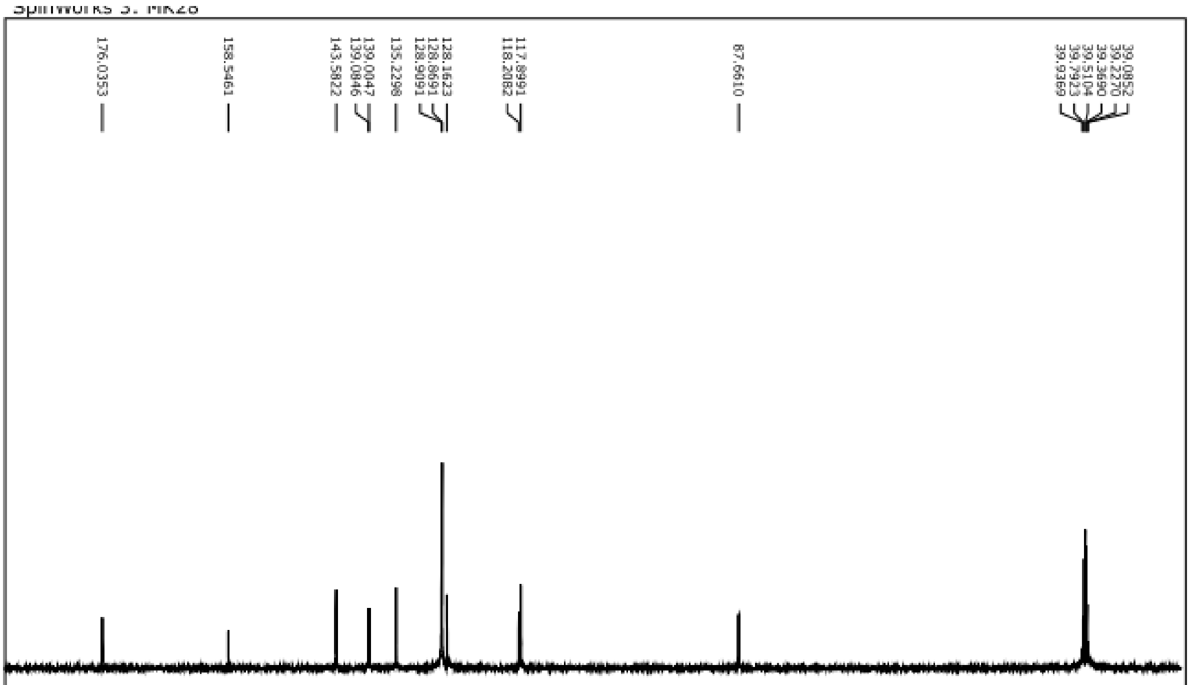

3-benzyl-6-iodo-2-mercaptoquinazolin-4(3H)-one (7e)

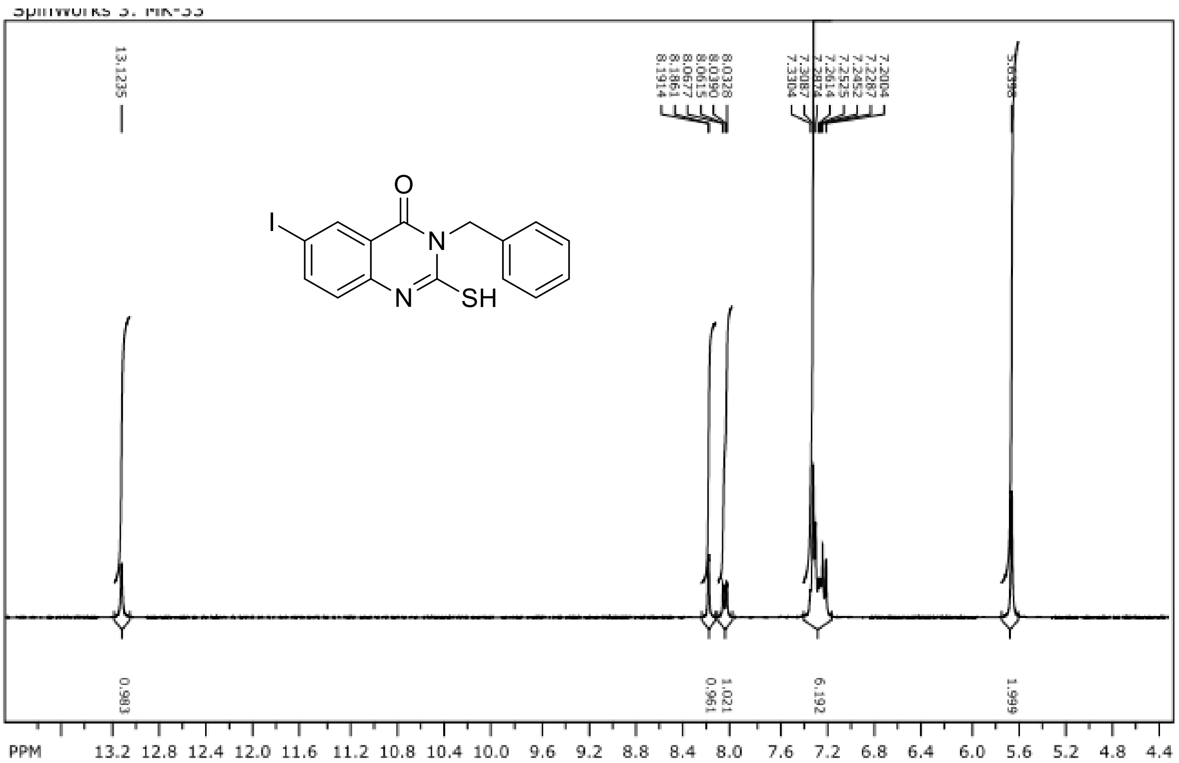

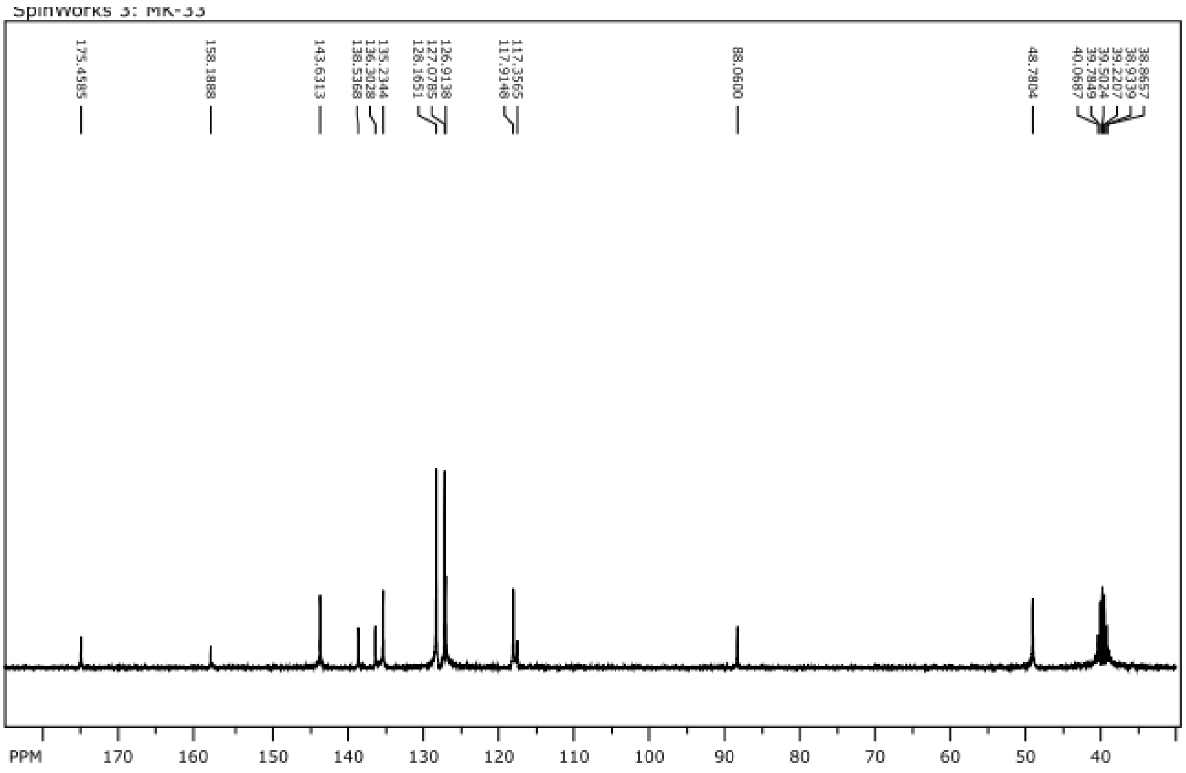

6-iodo-2-mercapto-3-(p-tolyl)quinazolin-4(3H)-one (7f)

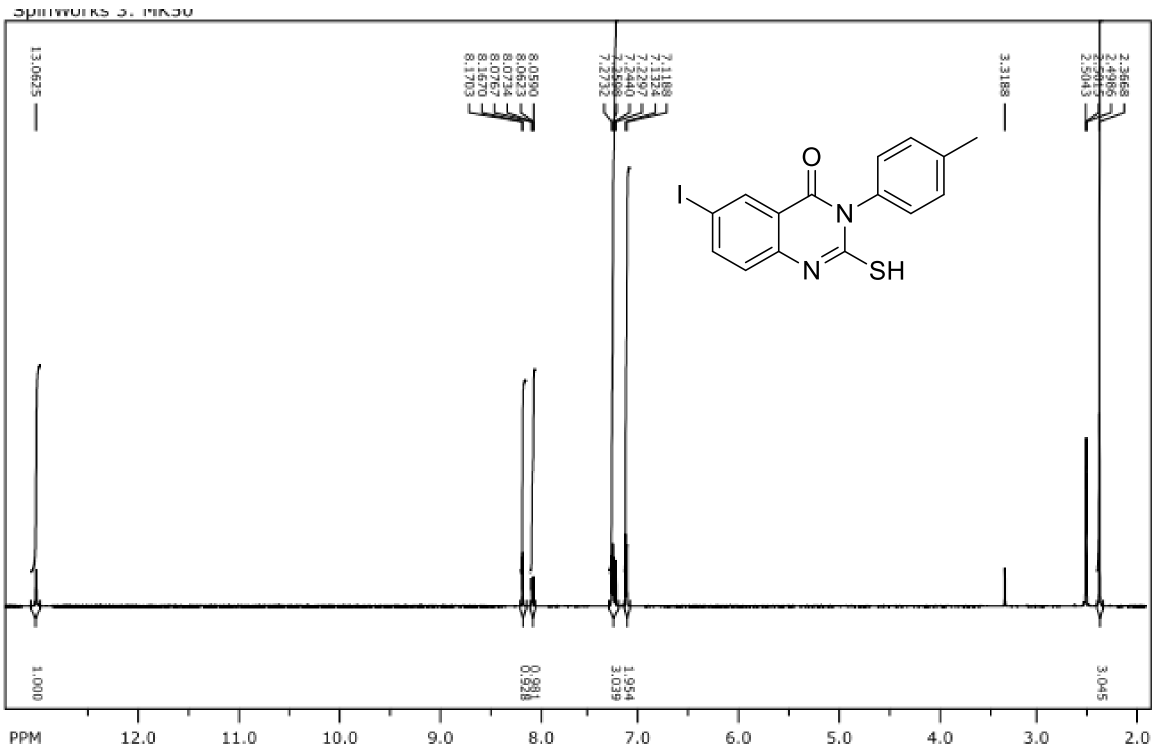

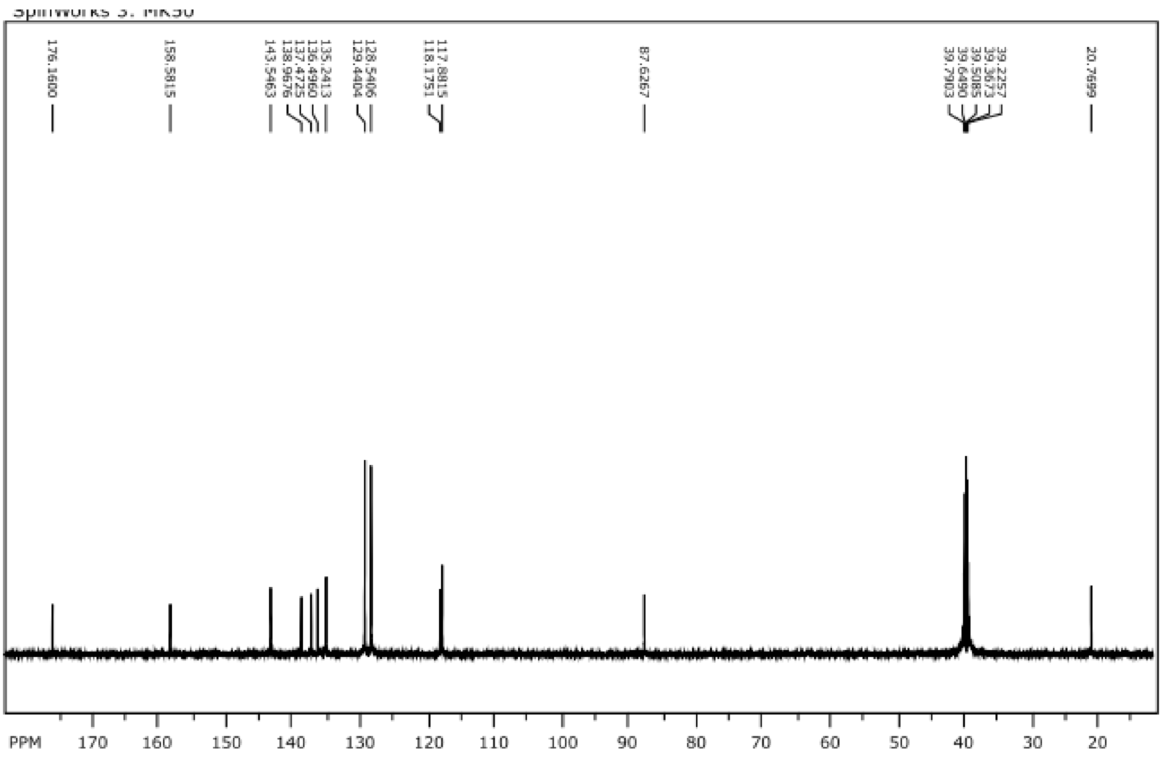

3-(4-fluorophenyl)-6-iodo-2-mercaptoquinazolin-4(3H)-one (7g)

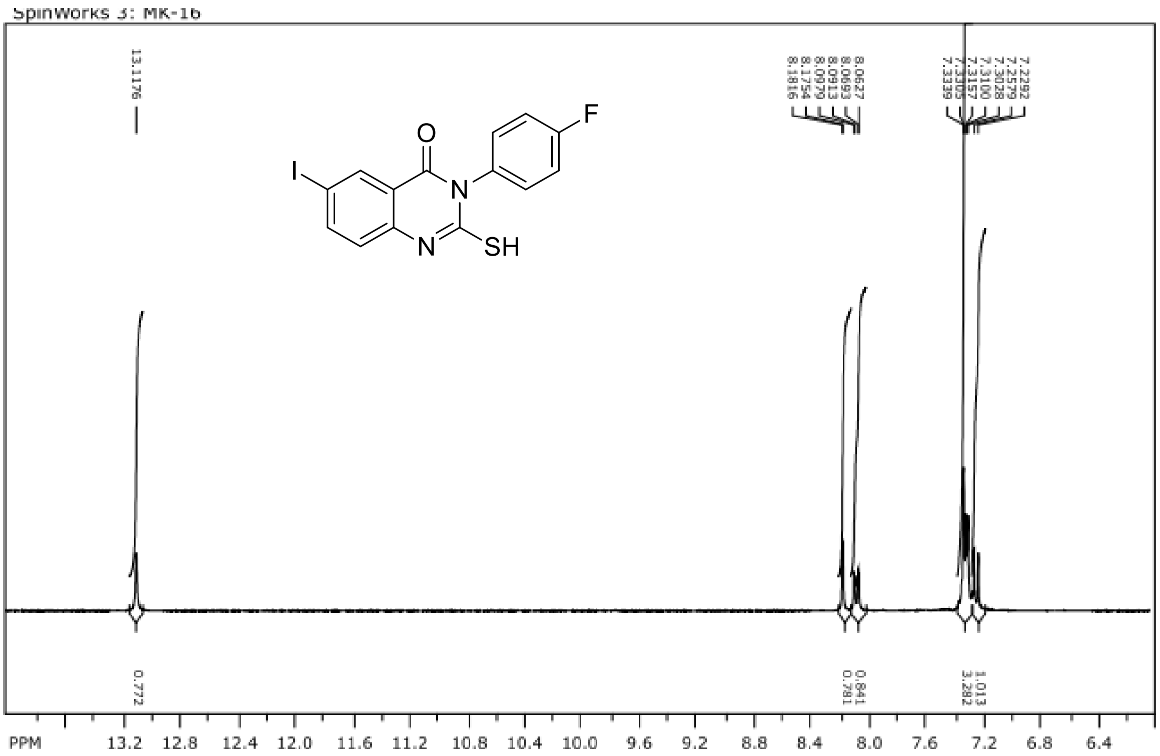

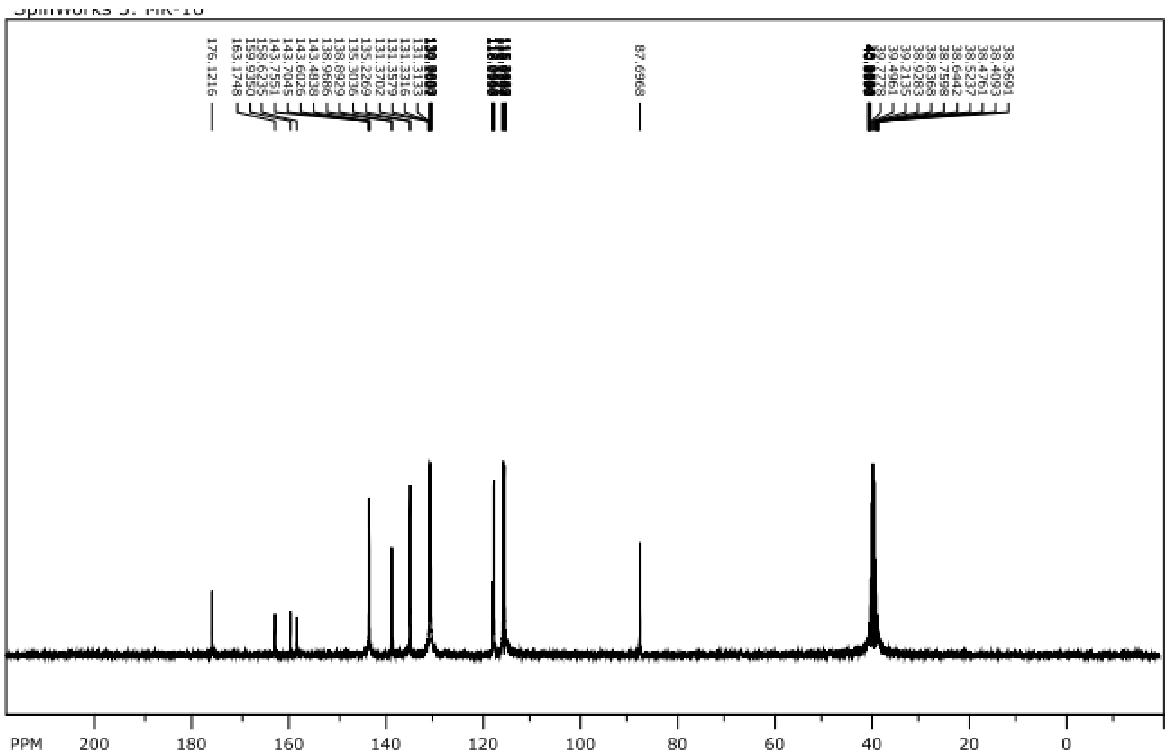

3-(4-chlorophenyl)-6-iodo-2-mercaptoquinazolin-4(3H)-one (7h)

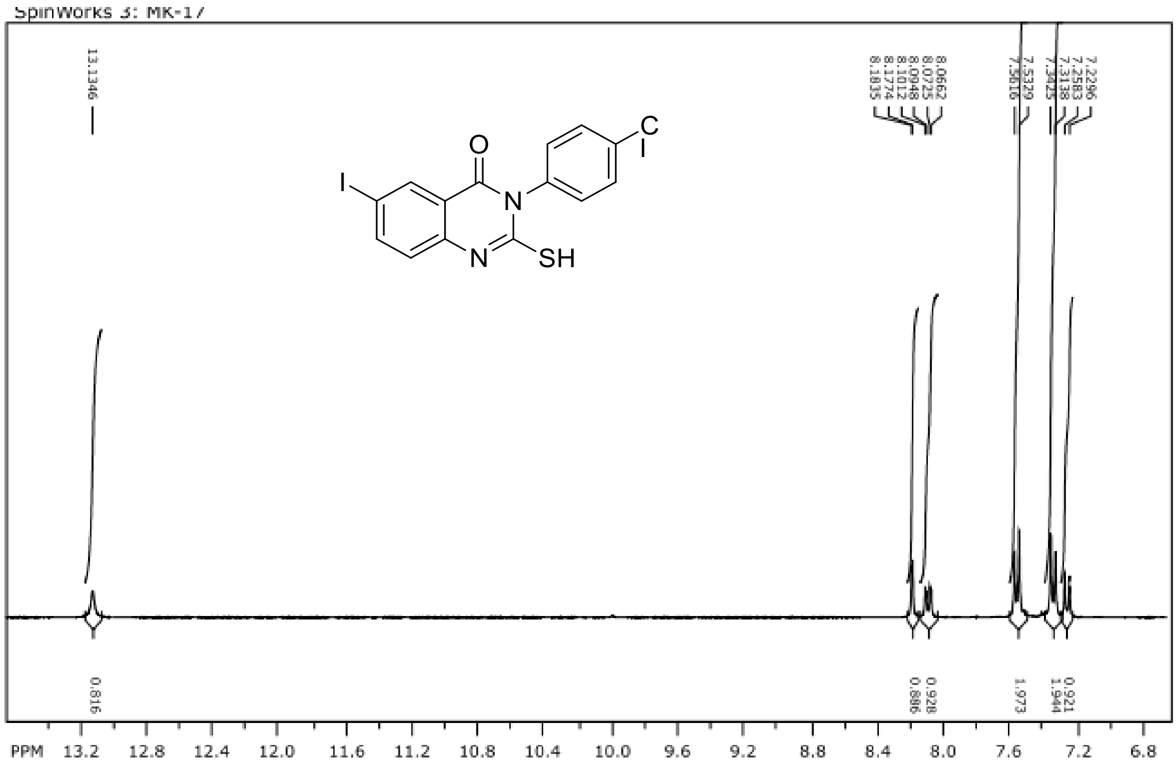

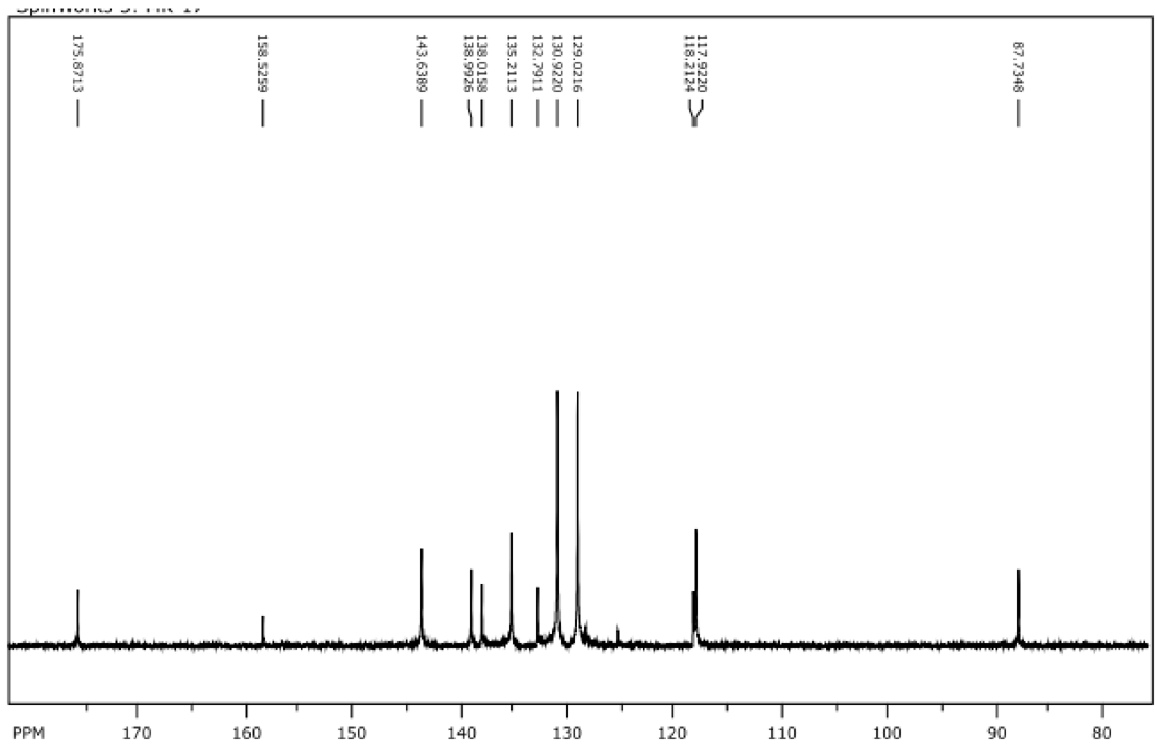

3-(4-bromophenyl)-6-iodo-2-mercaptoquinazolin-4(3H)-one (7i)

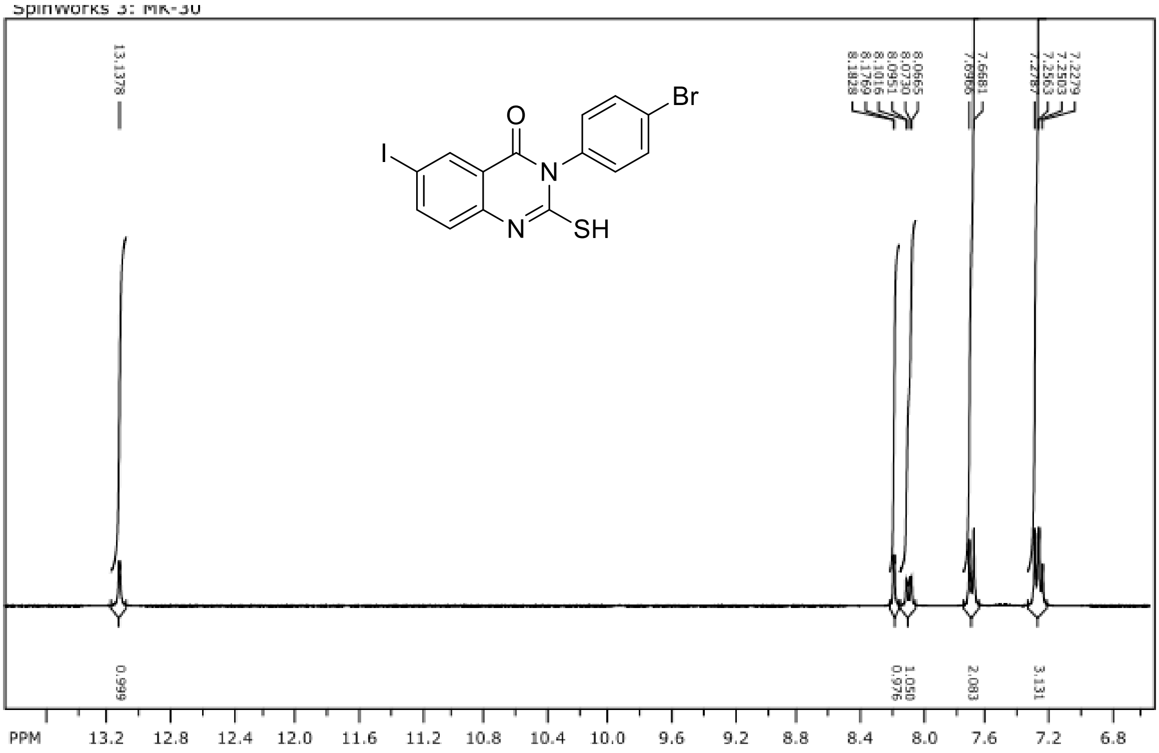

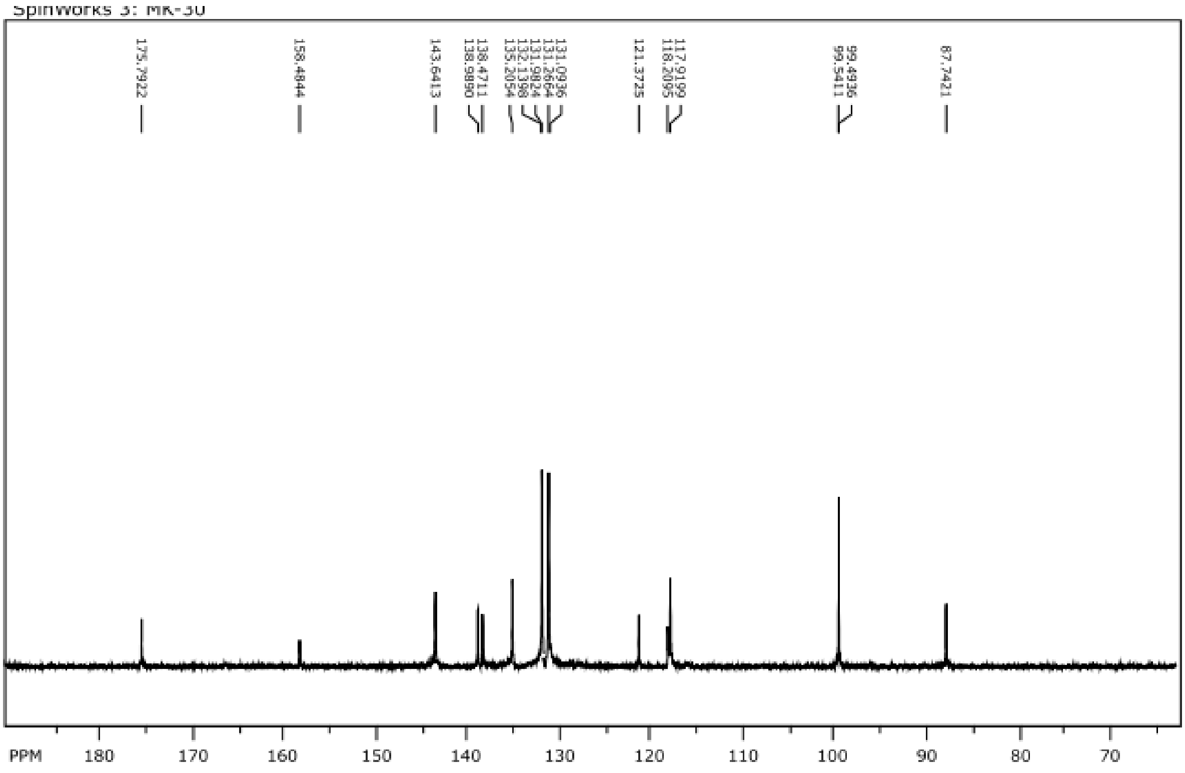

6-iodo-2-mercapto-3-(3-methoxyphenyl)quinazolin-4(3H)-one (7j)

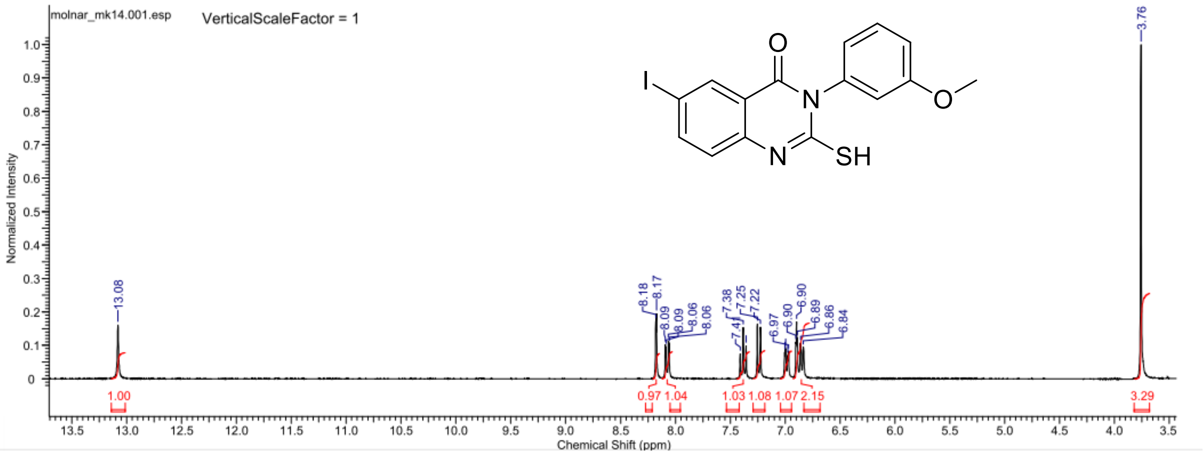

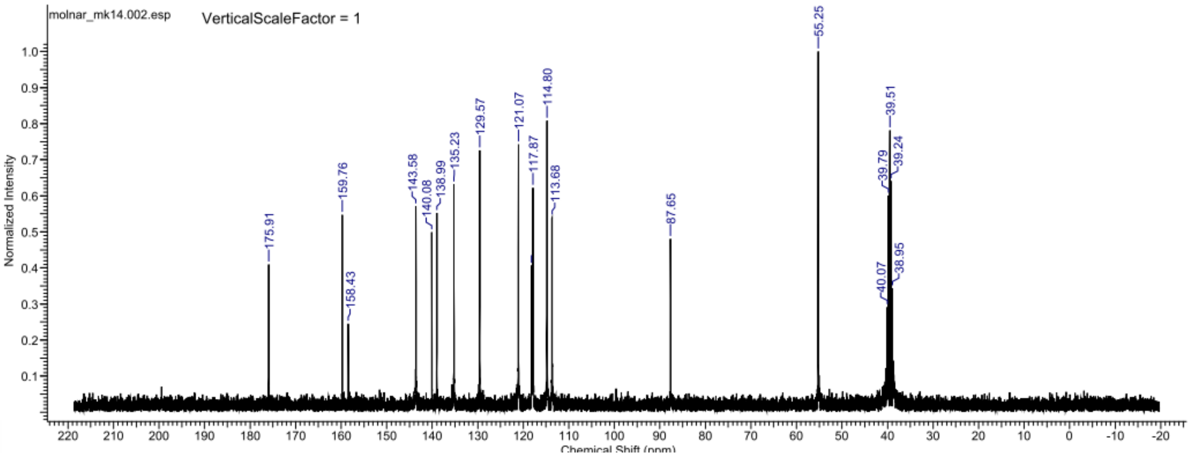

3-(3-chlorophenyl)-6-iodo-2-mercaptoquinazolin-4(3H)-one (7k)

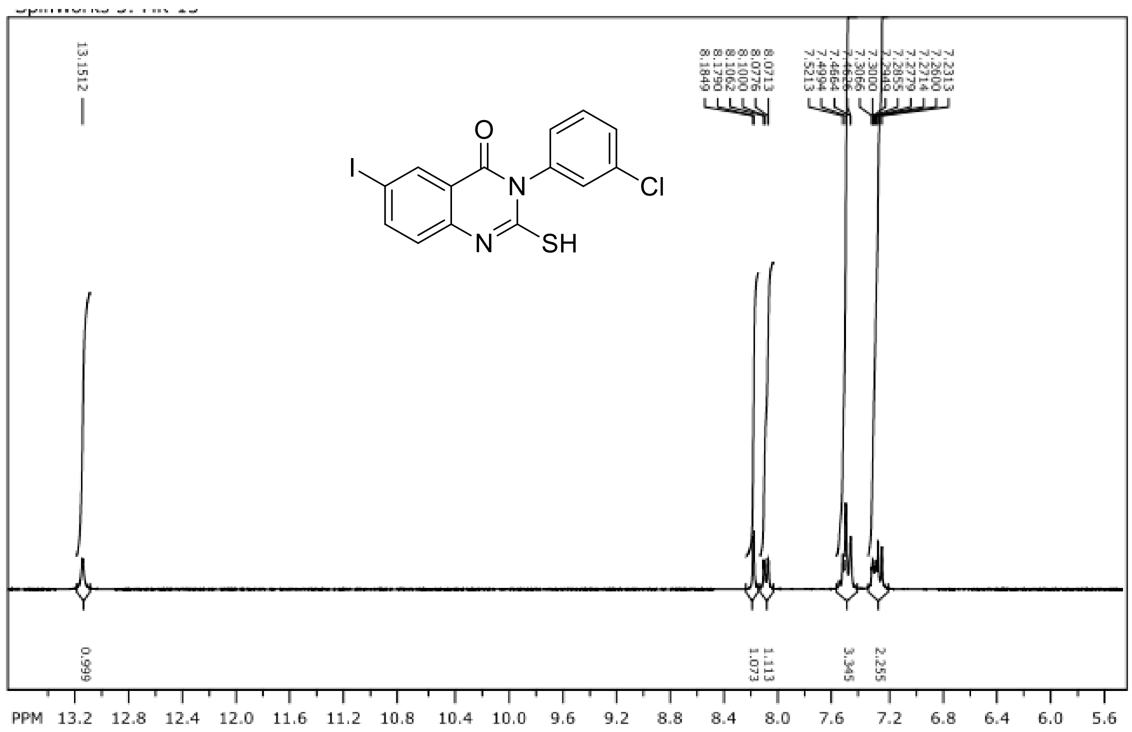

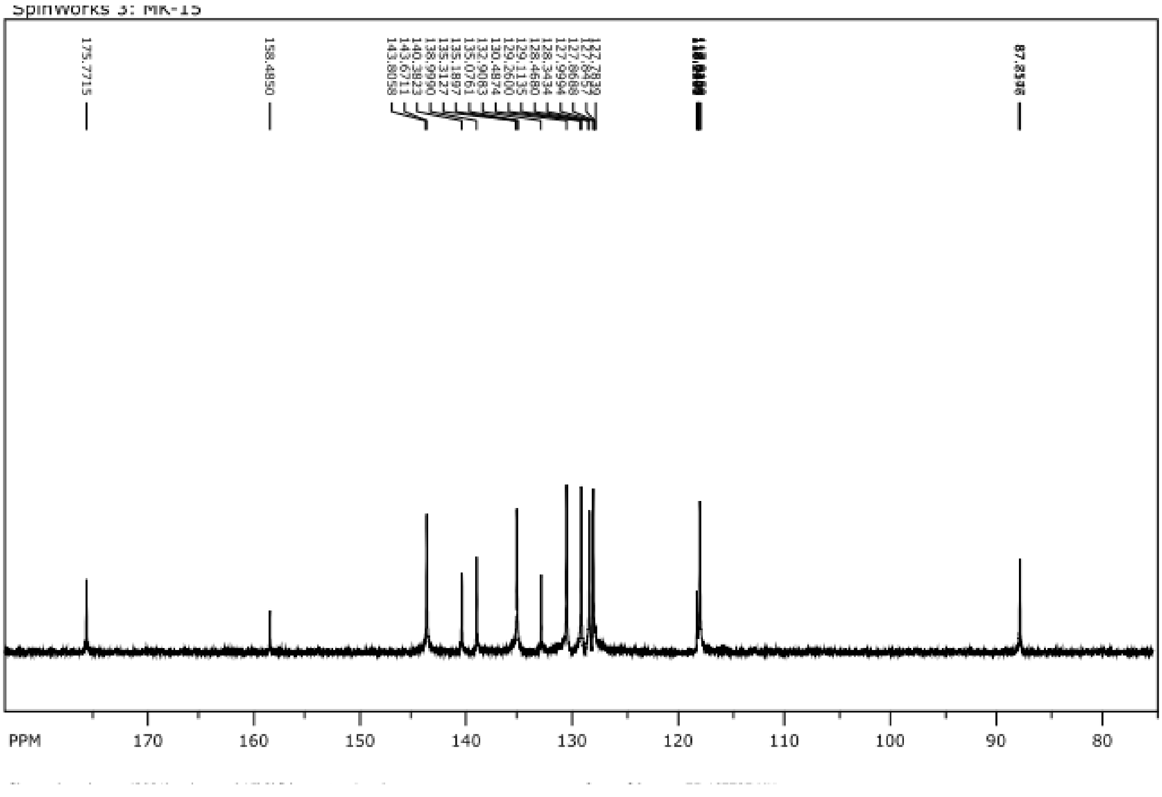

6-bromo-2-mercapto-3-methylquinazolin-4(3H)-one (8a)

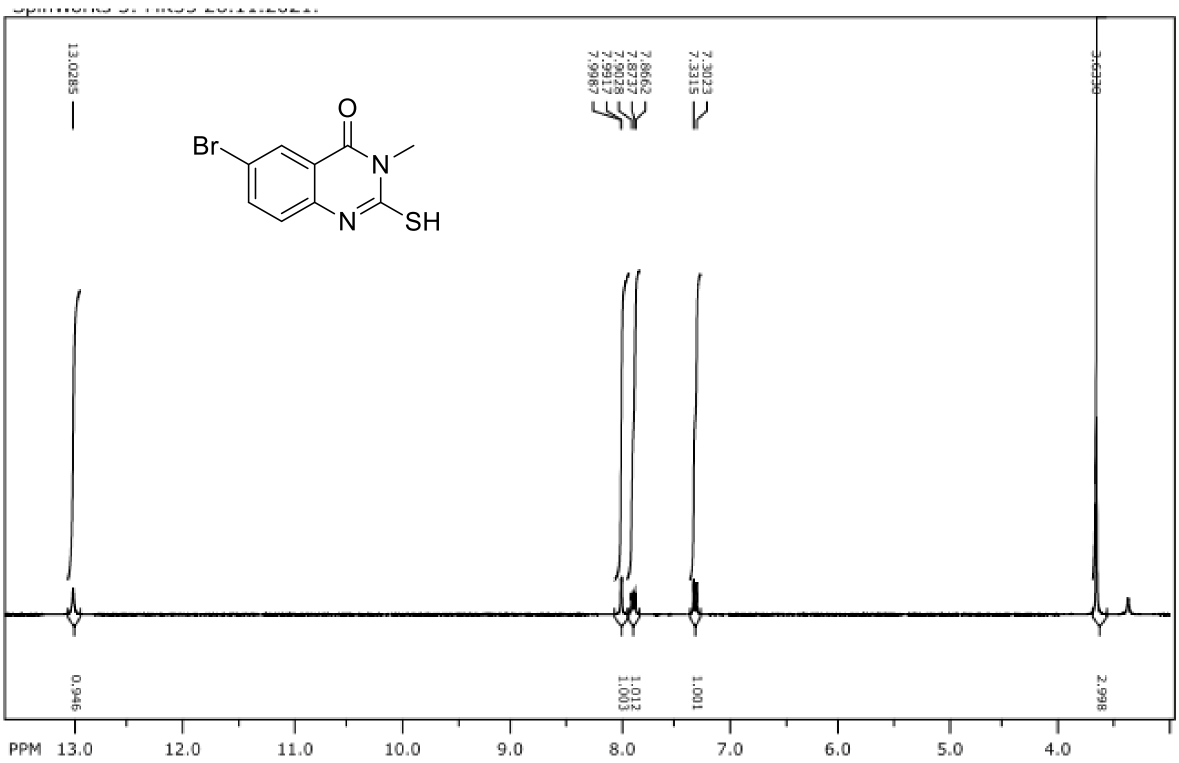

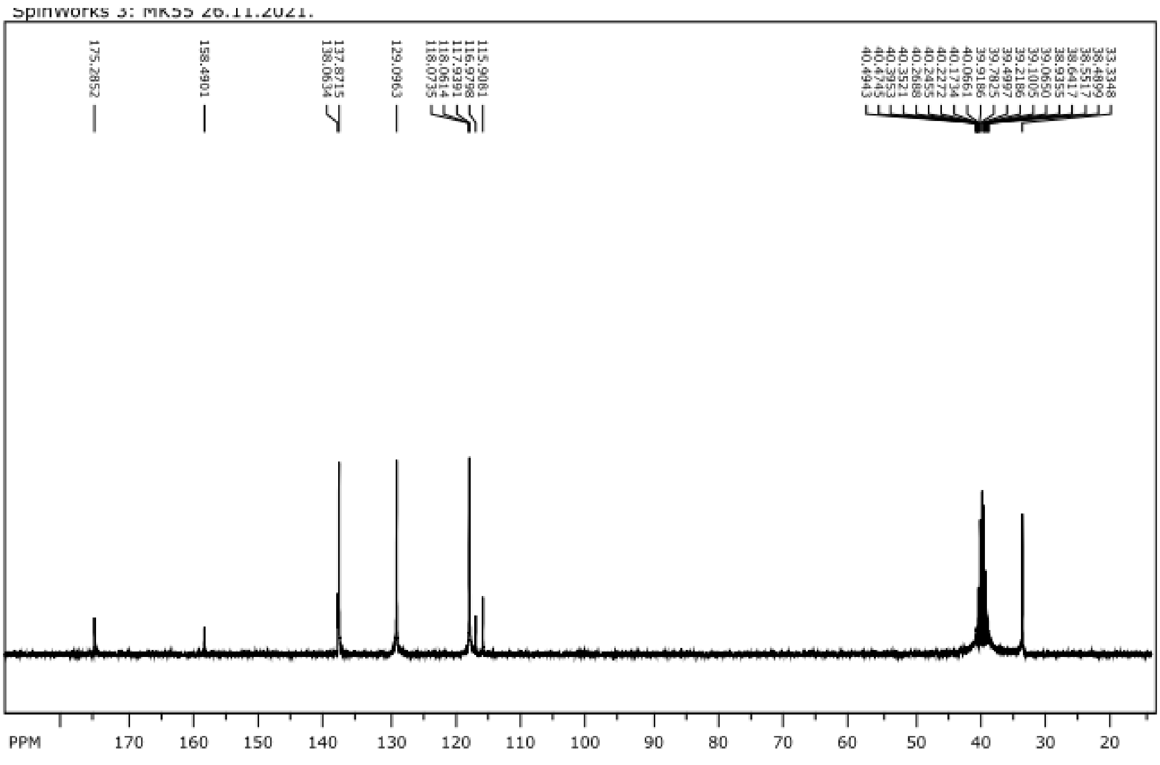

6-bromo-3-ethyl-2-mercaptoquinazolin-4(3H)-one (8b)

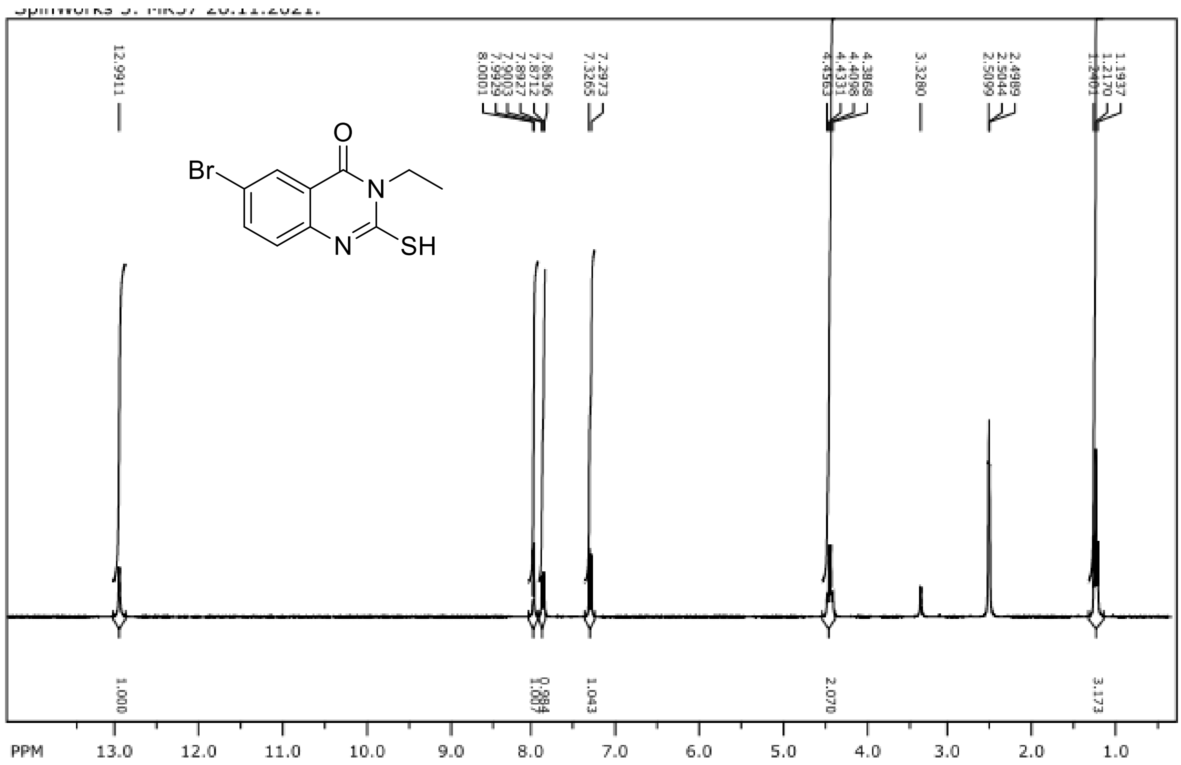

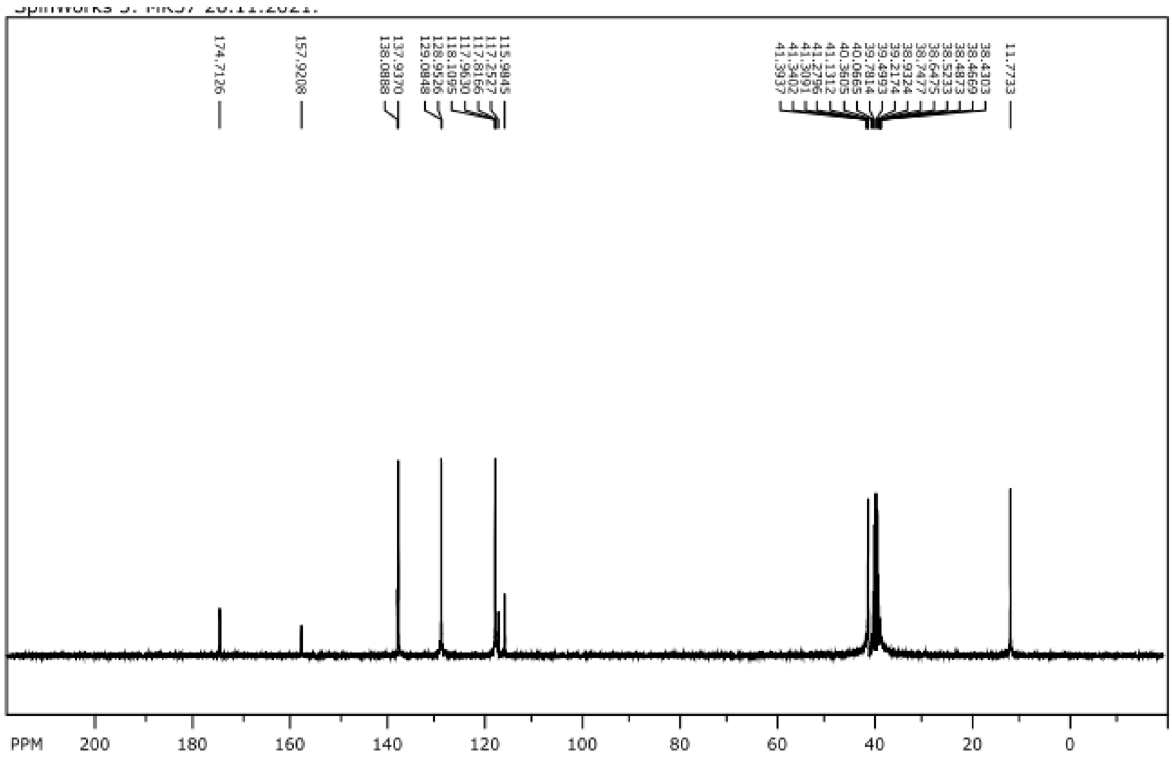

3-allyl-6-bromo-2-mercaptoquinazolin-4(3H)-one (8c)

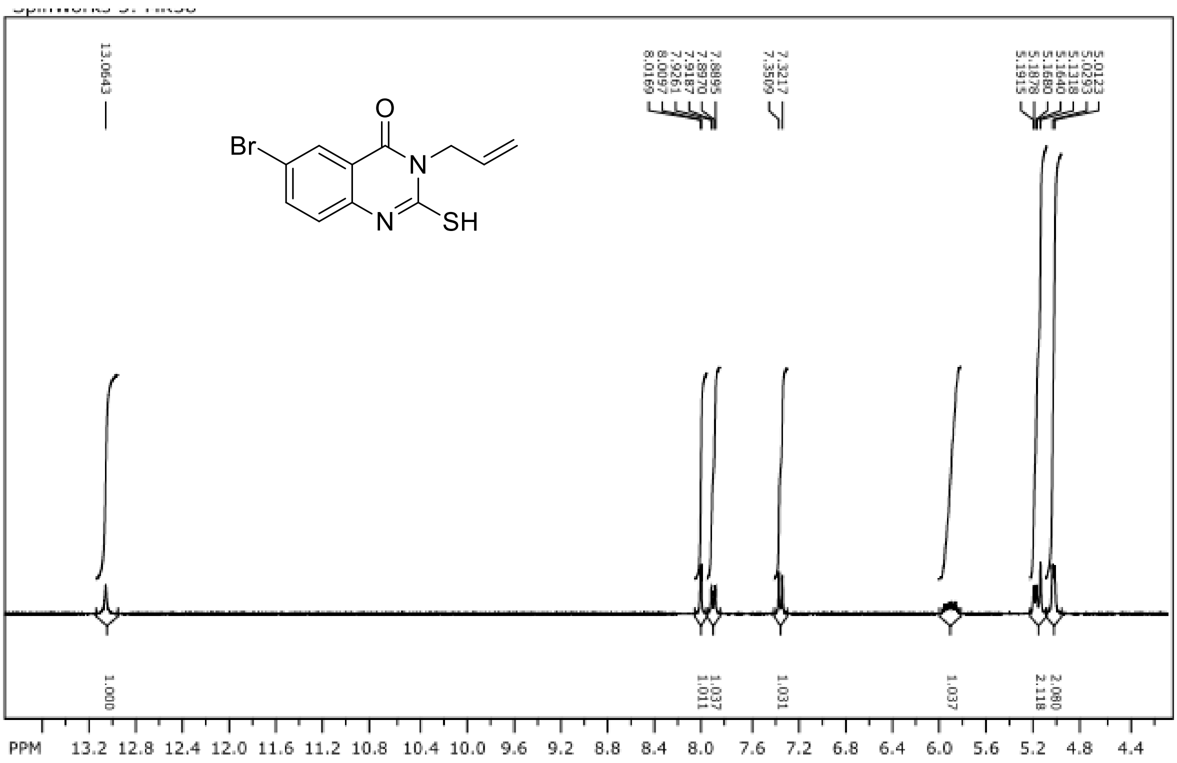

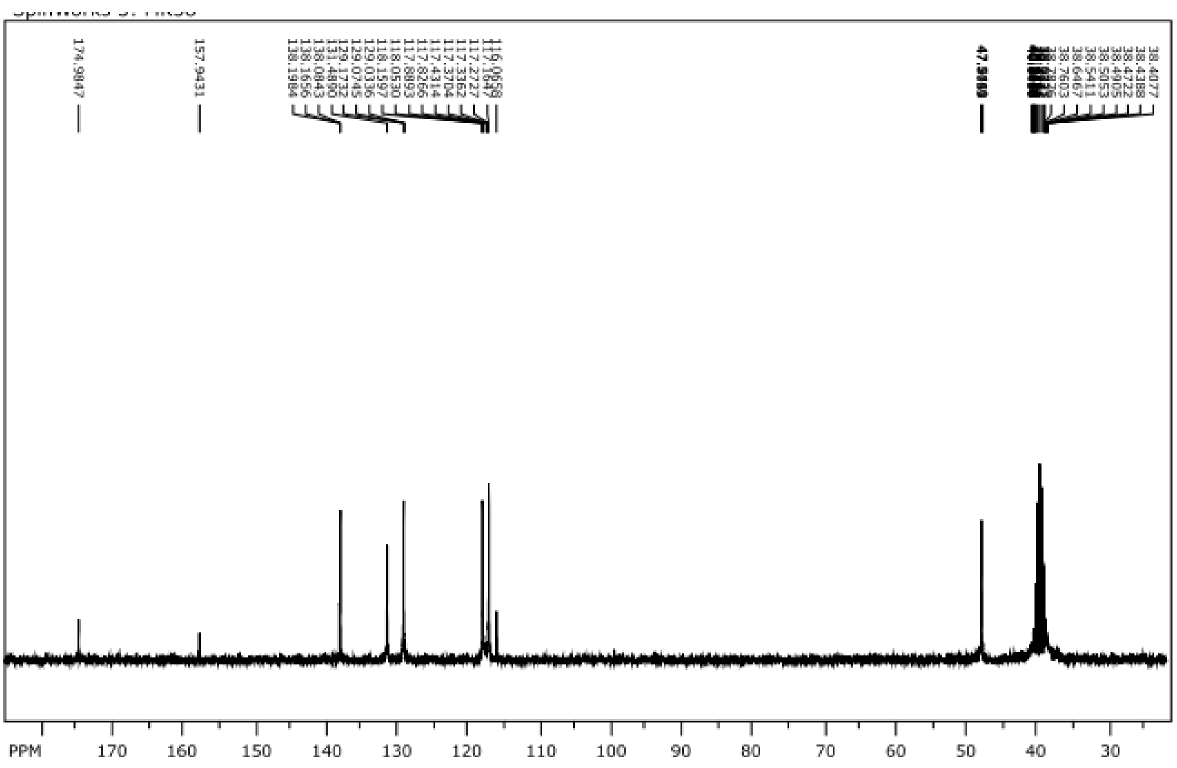

6-bromo-2-mercapto-3-phenylquinazolin-4(3H)-one (8d)

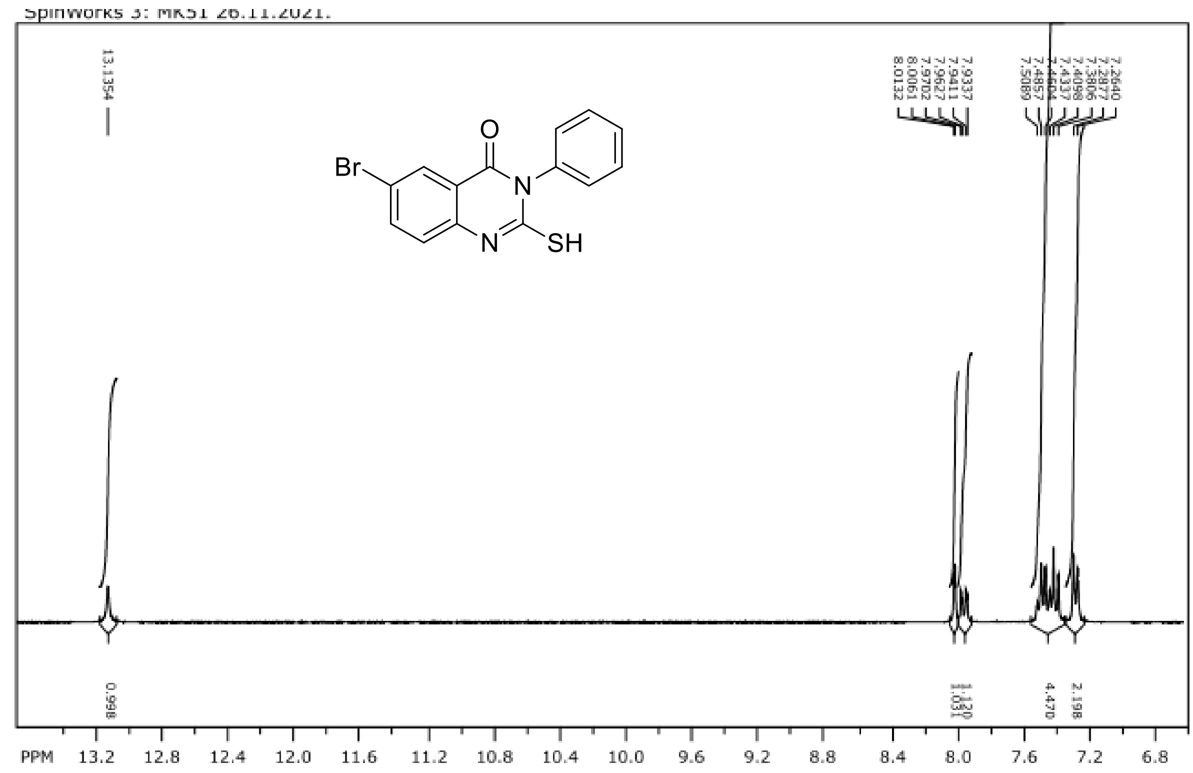

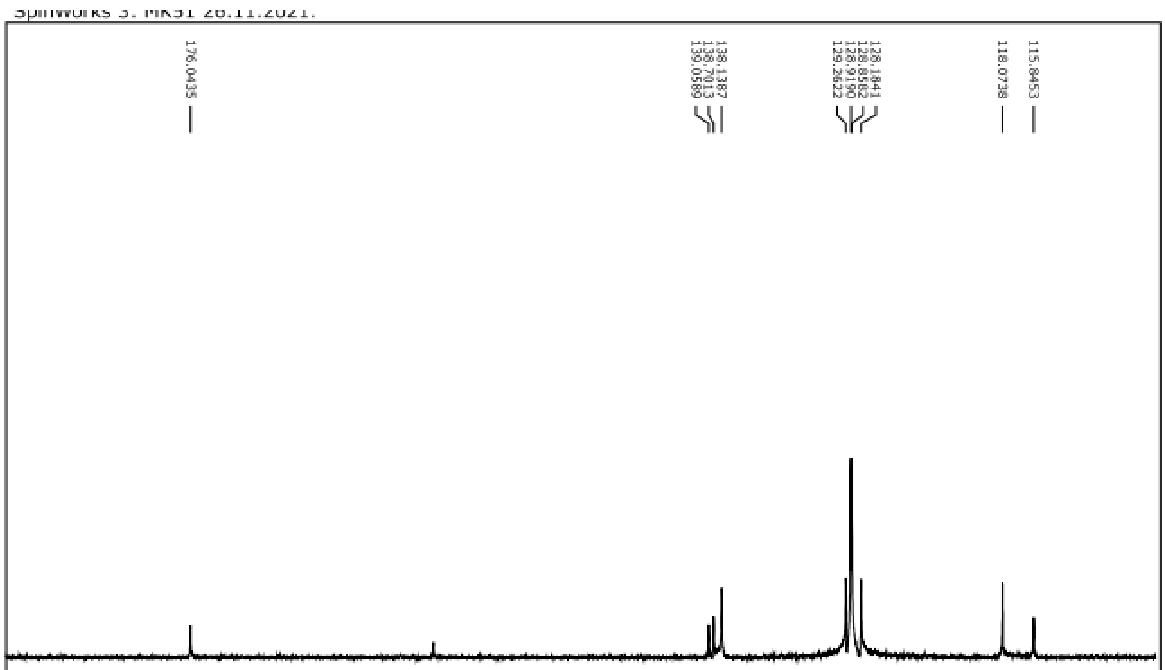

3-benzyl-6-bromo-2-mercaptoquinazolin-4(3H)-one (8e)

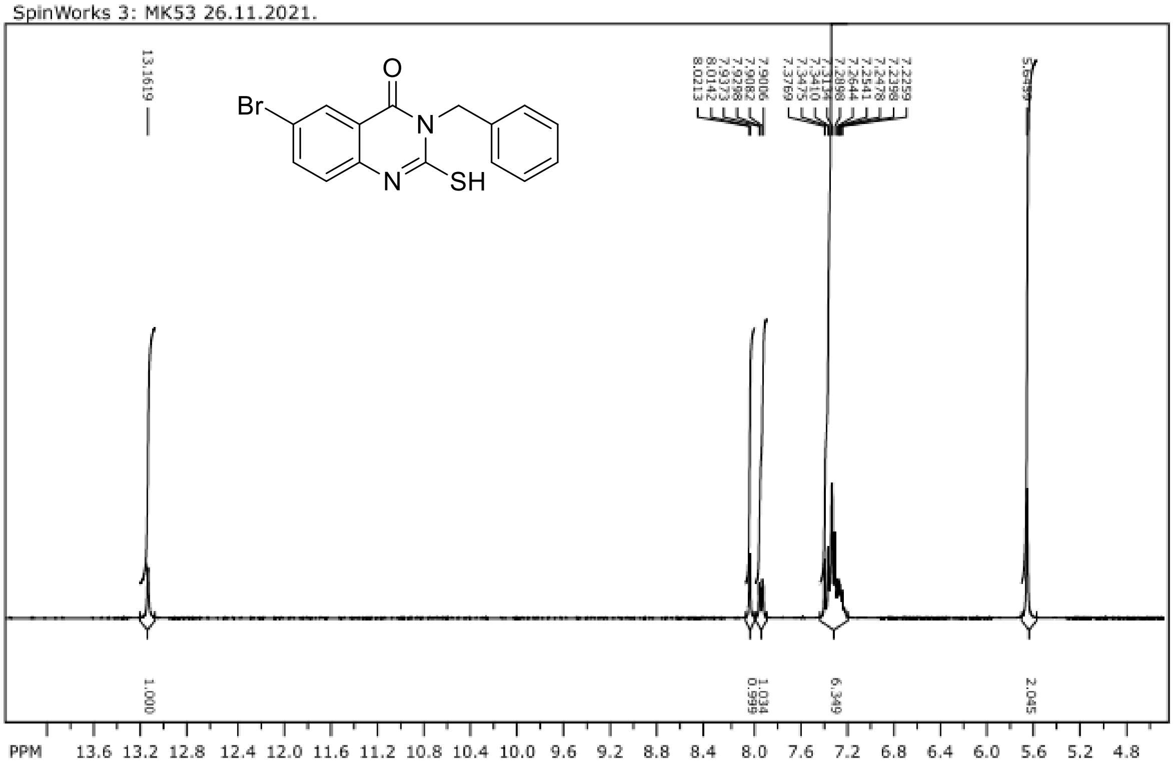

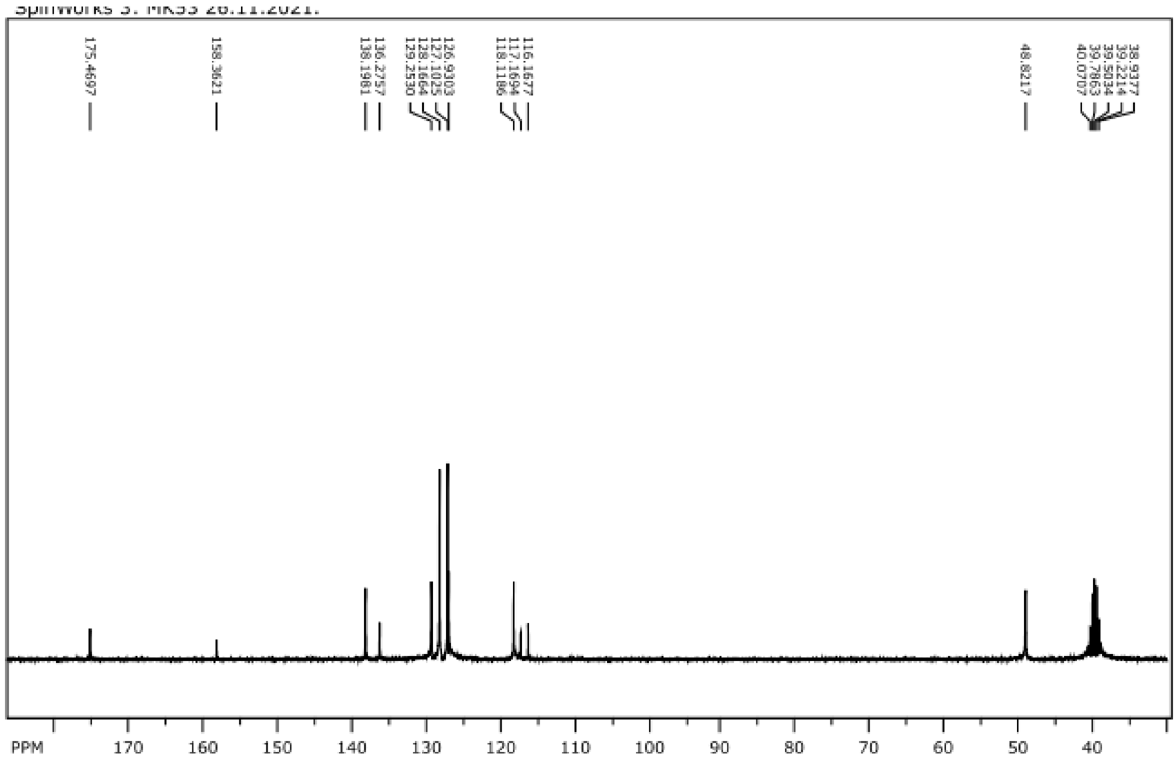

6-bromo-2-mercapto-3-(p-tolyl)quinazolin-4(3H)-one (8f)

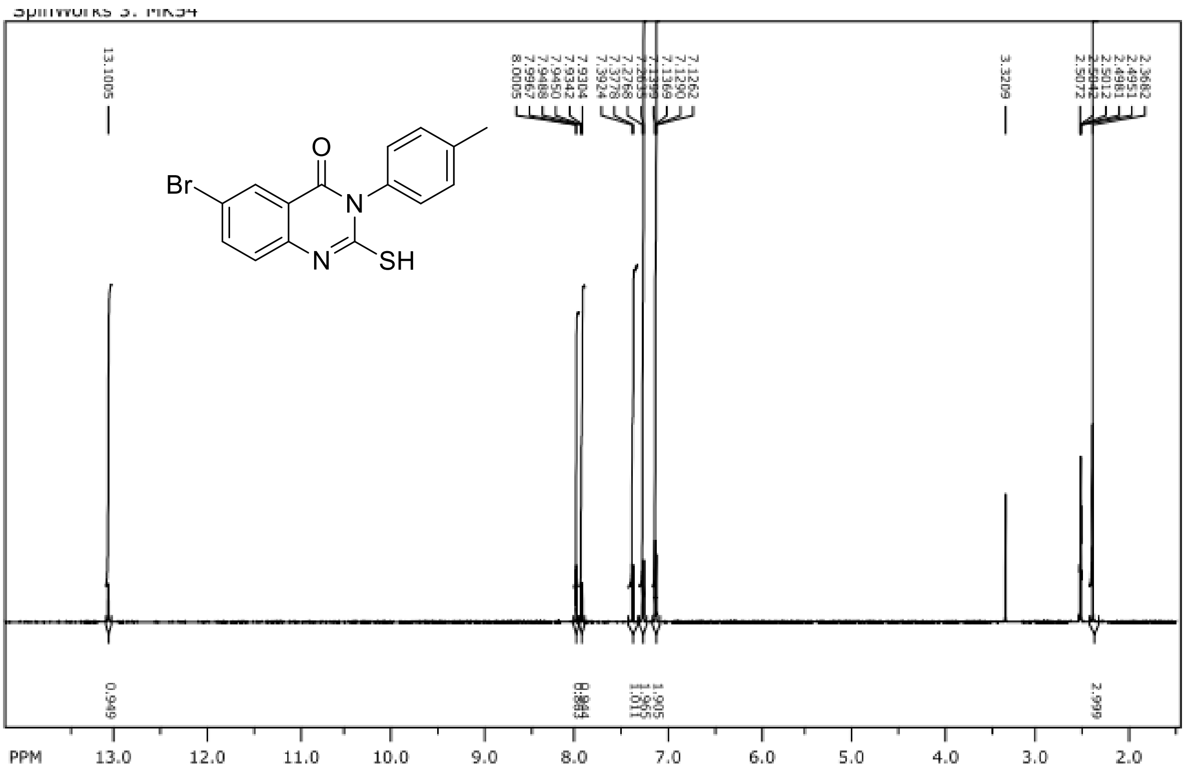

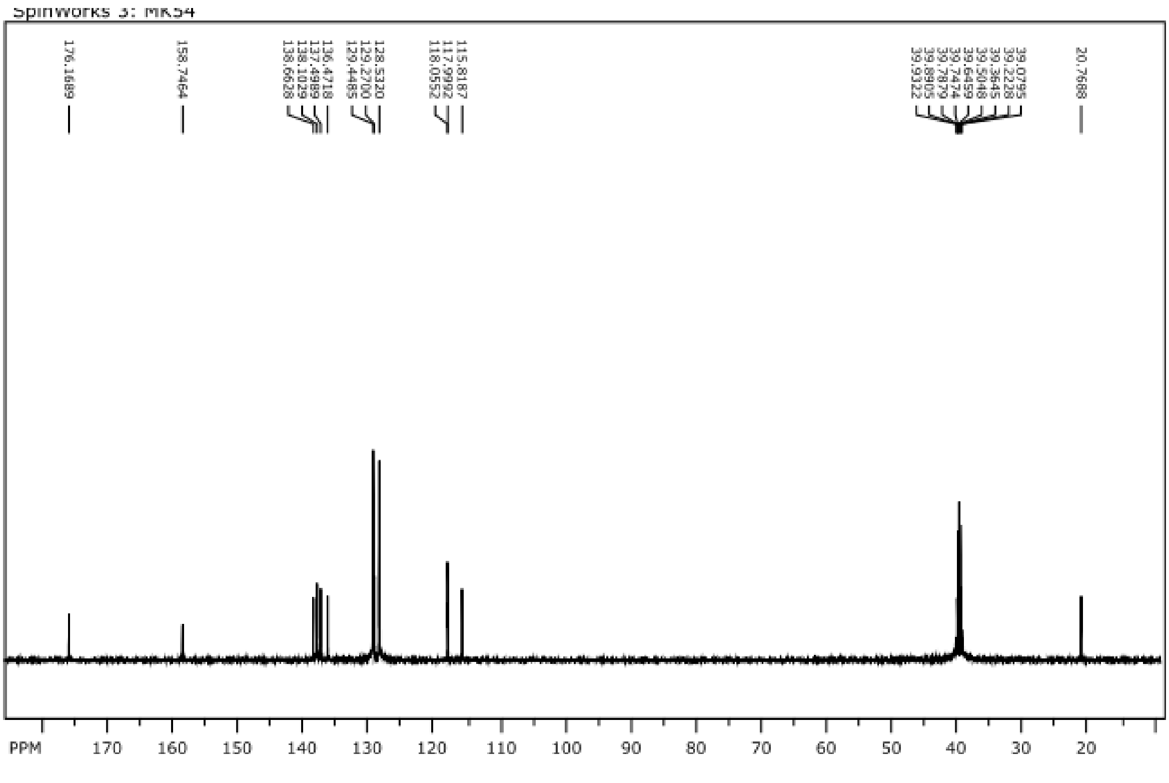

6-bromo-3-(4-fluorophenyl)-2-mercaptoquinazolin-4(3H)-one (8g)

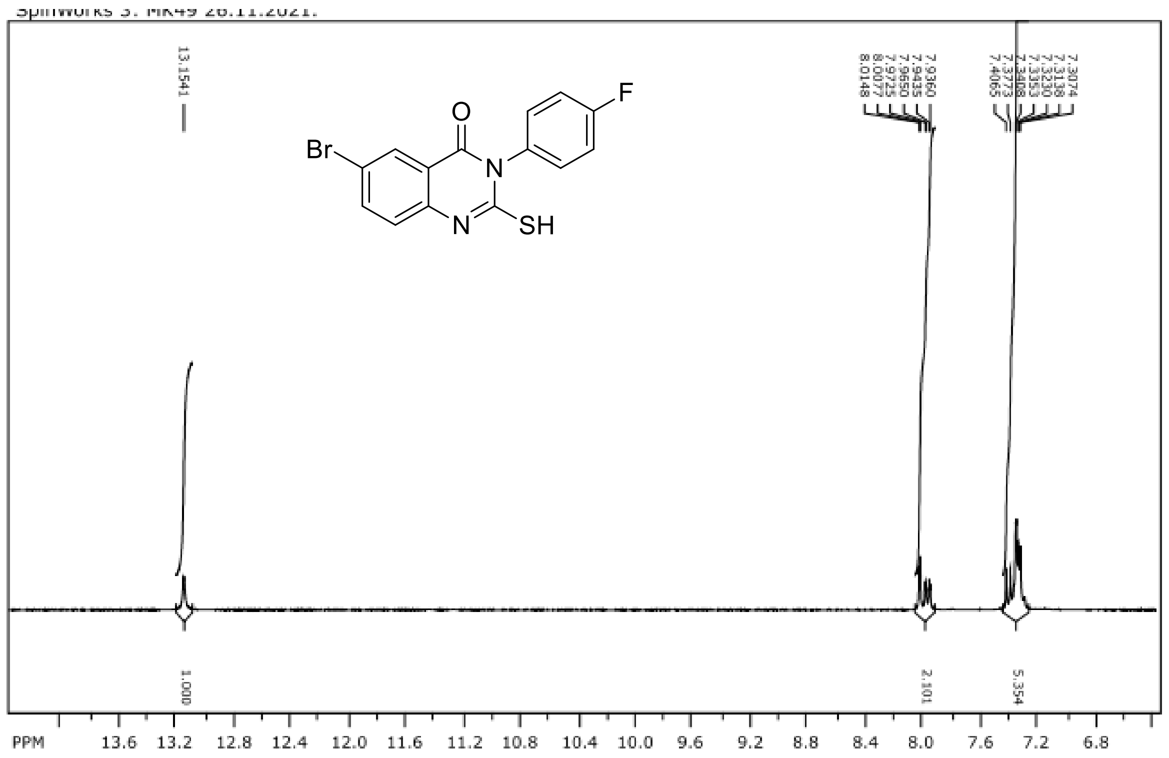

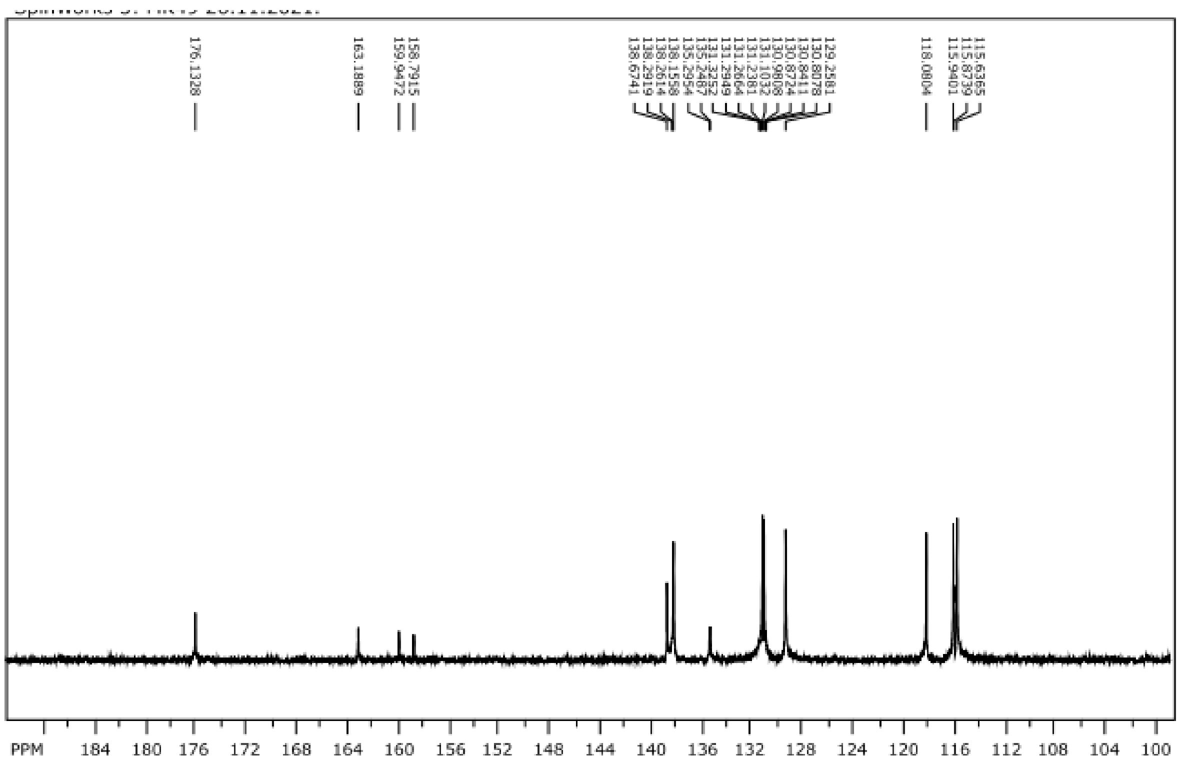

6-bromo-3-(4-chlorophenyl)-2-mercaptoquinazolin-4(3H)-one (8h)

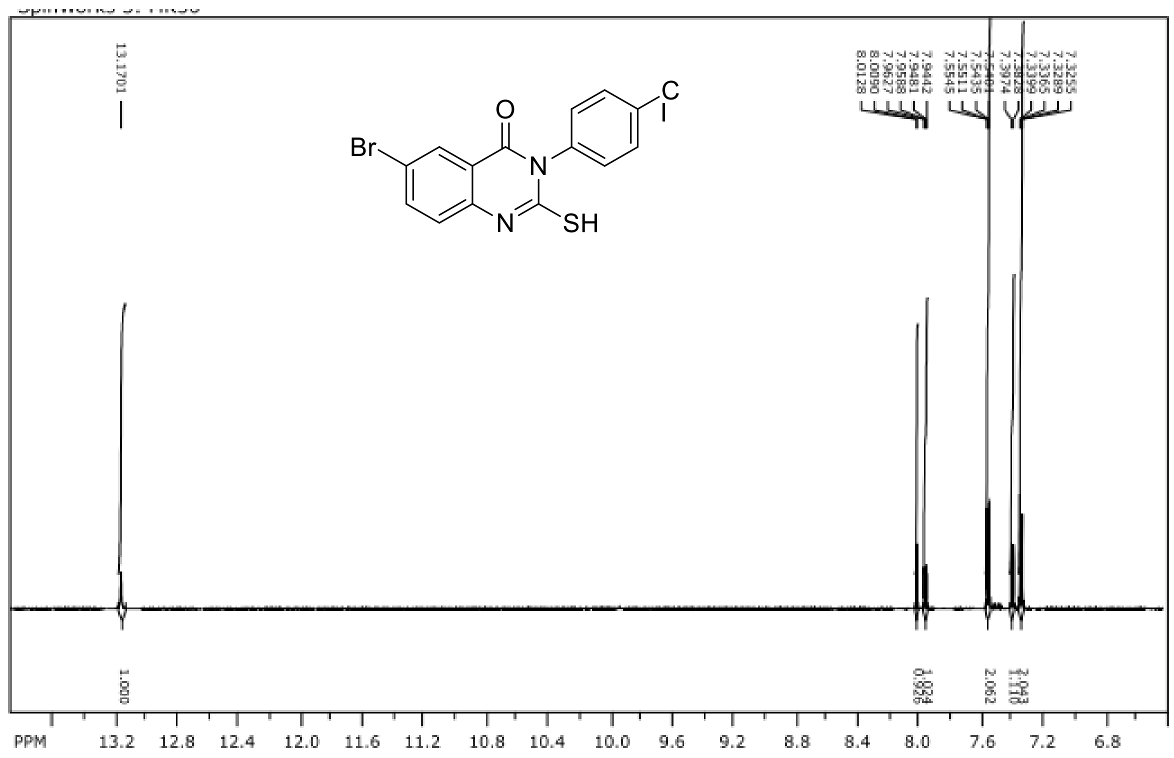

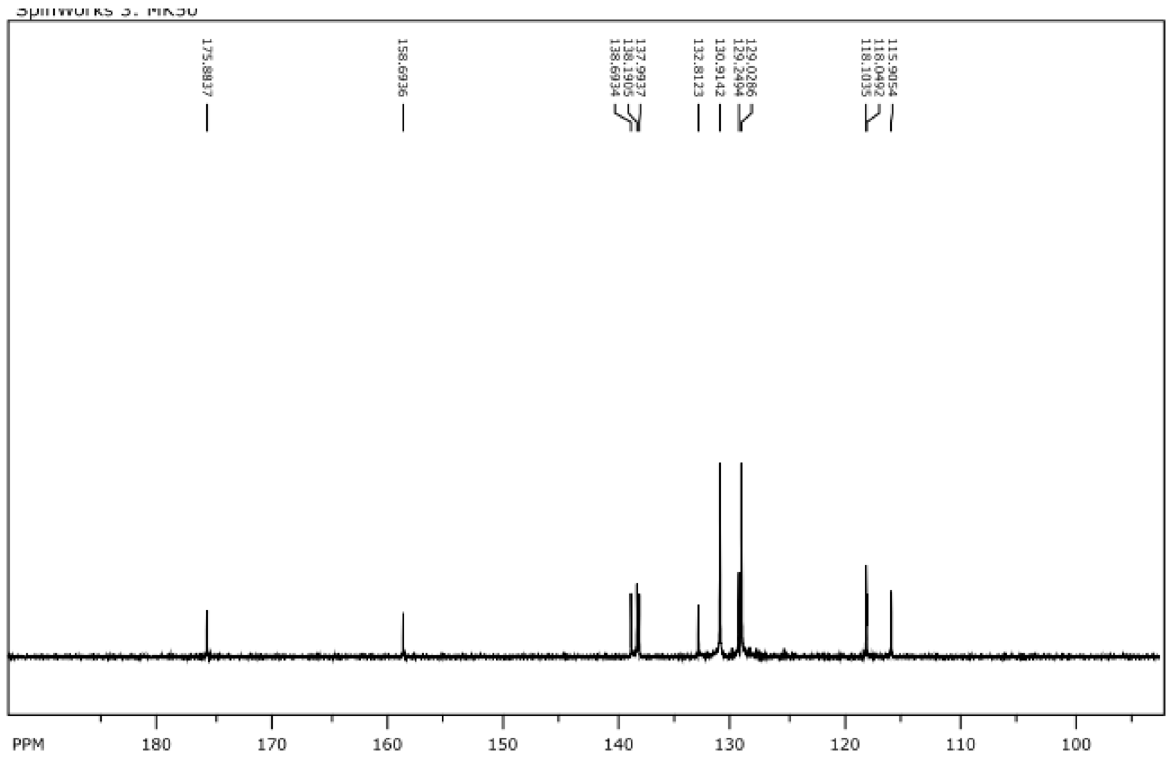

6-bromo-3-(4-bromophenyl)-2-mercaptoquinazolin-4(3H)-one (8i)

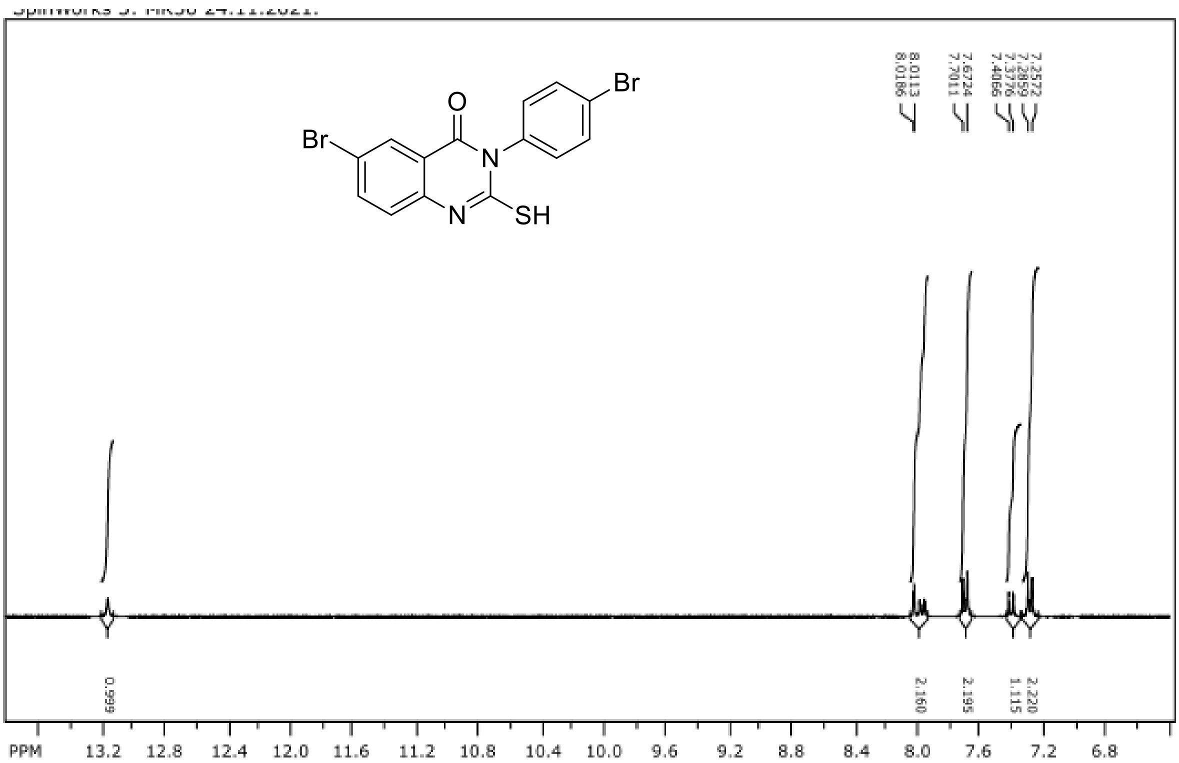

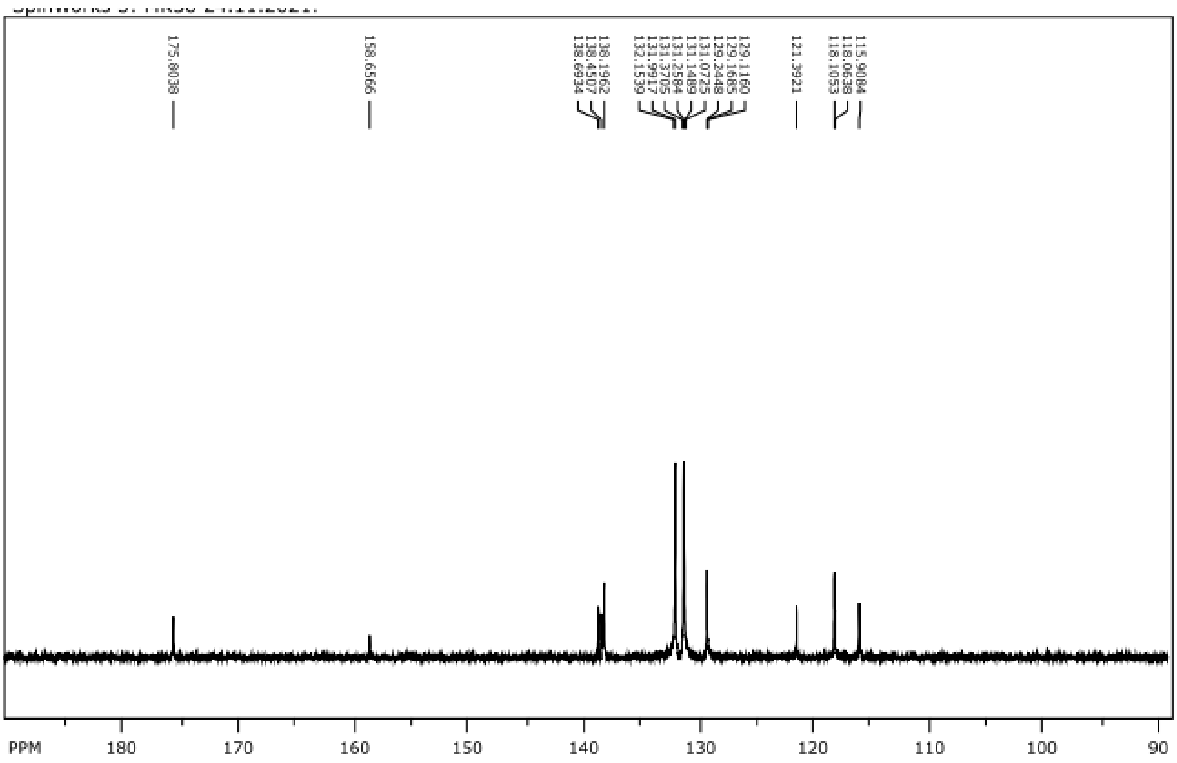

6-bromo-2-mercapto-3-(3-methoxyphenyl)quinazolin-4(3H)-one (8j)

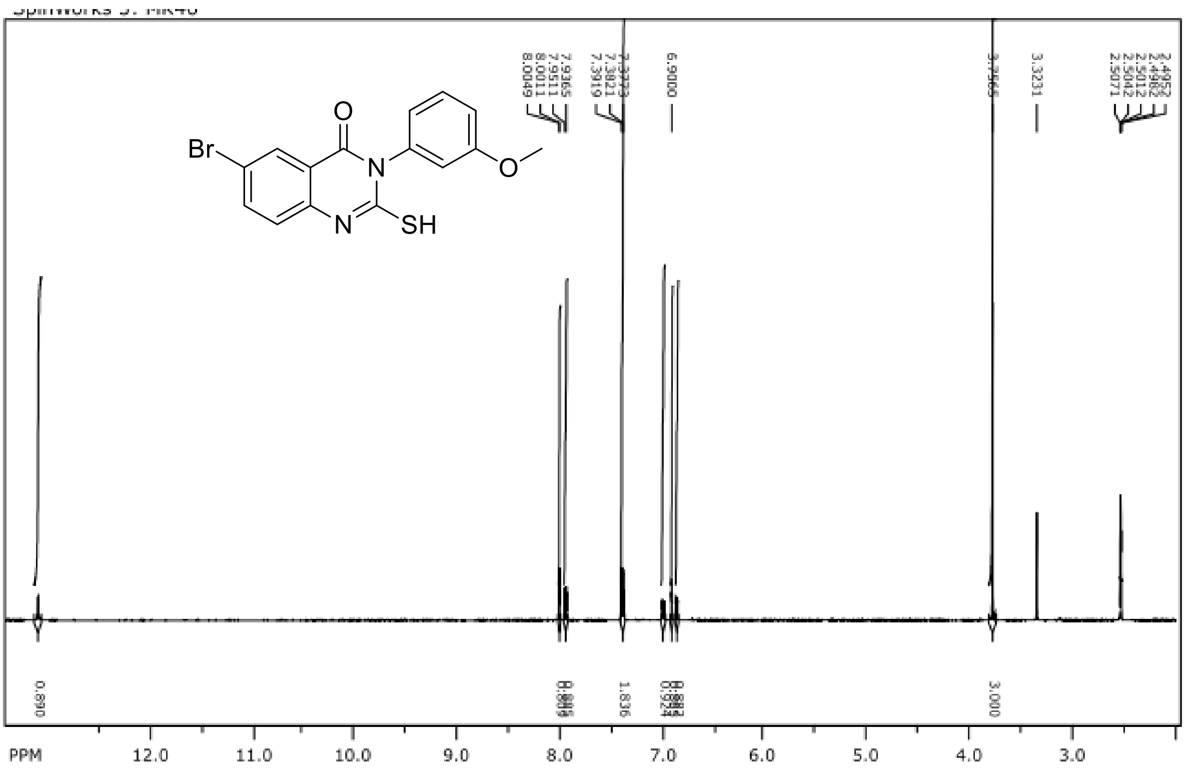

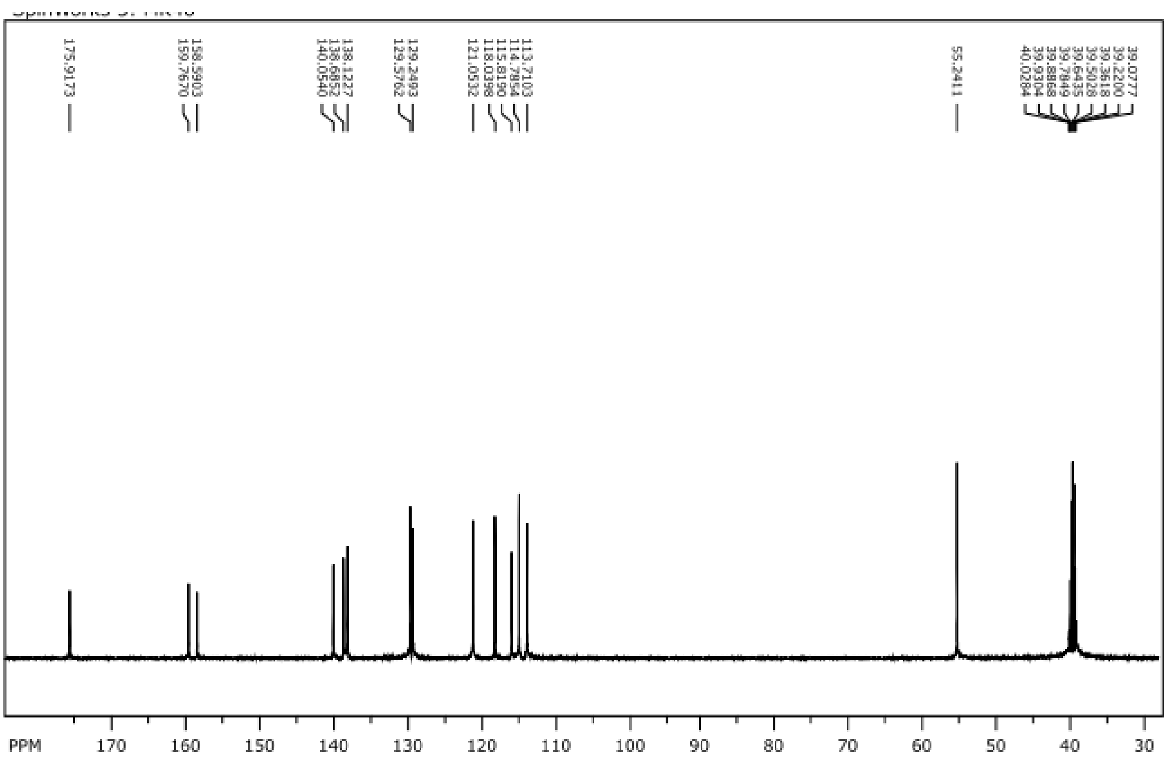

6-bromo-3-(3-chlorophenyl)-2-mercaptoquinazolin-4(3H)-one (8k)

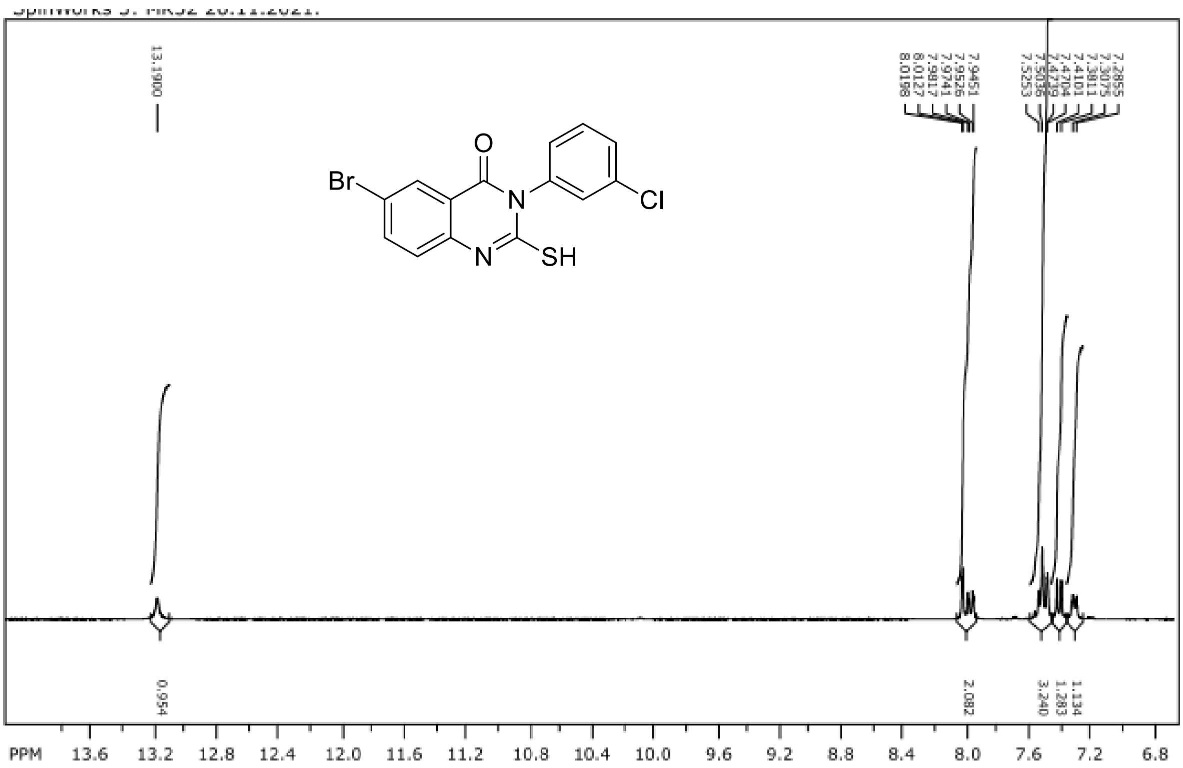

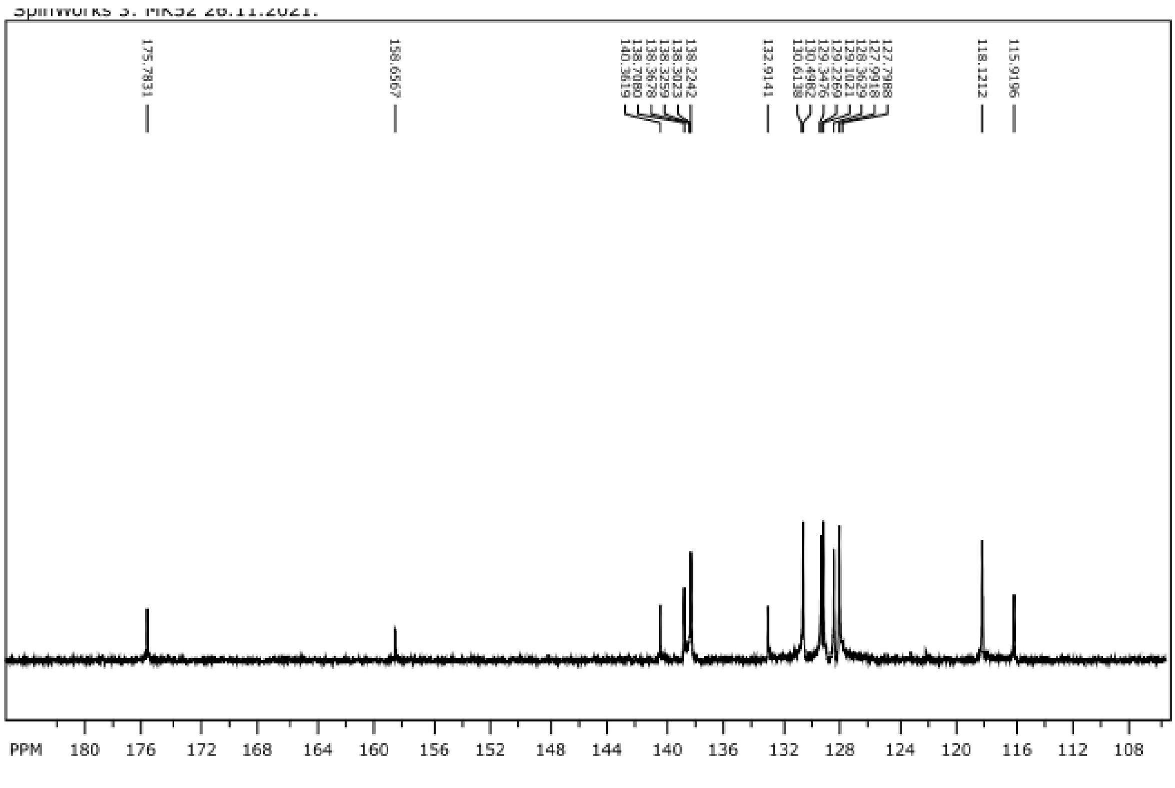

7-chloro-2-mercapto-3-methylquinazolin-4(3H)-one (9a)

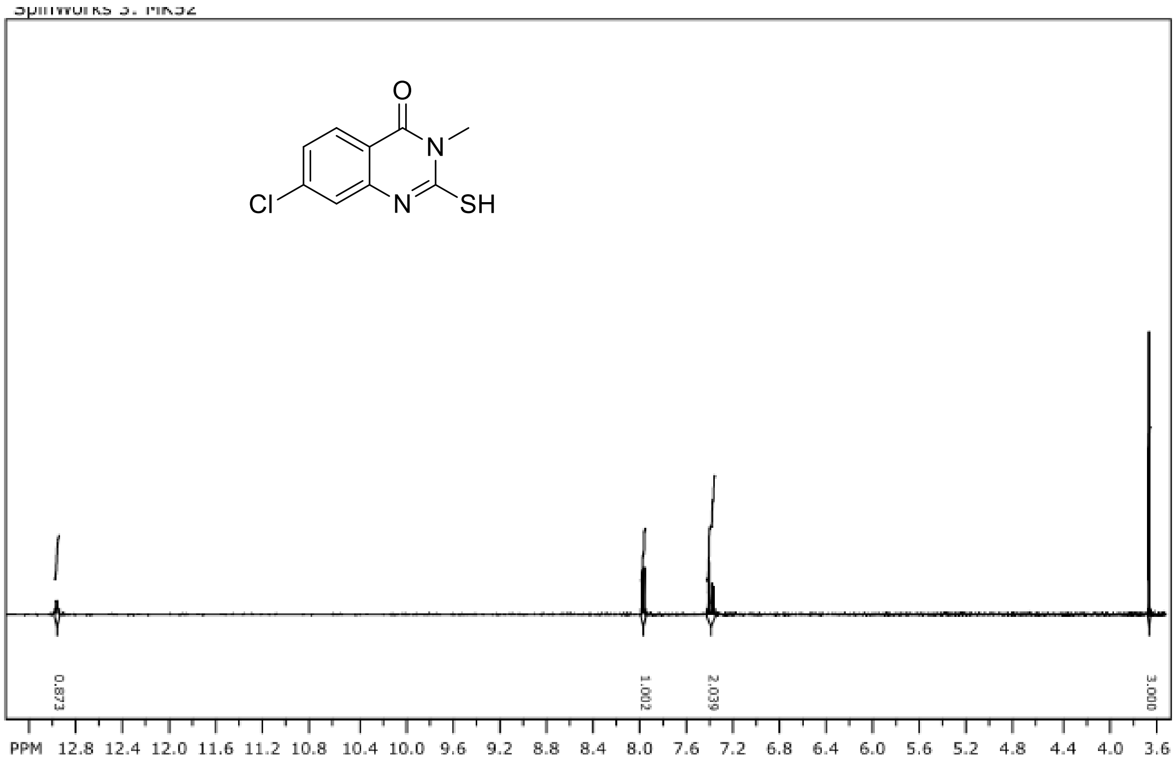

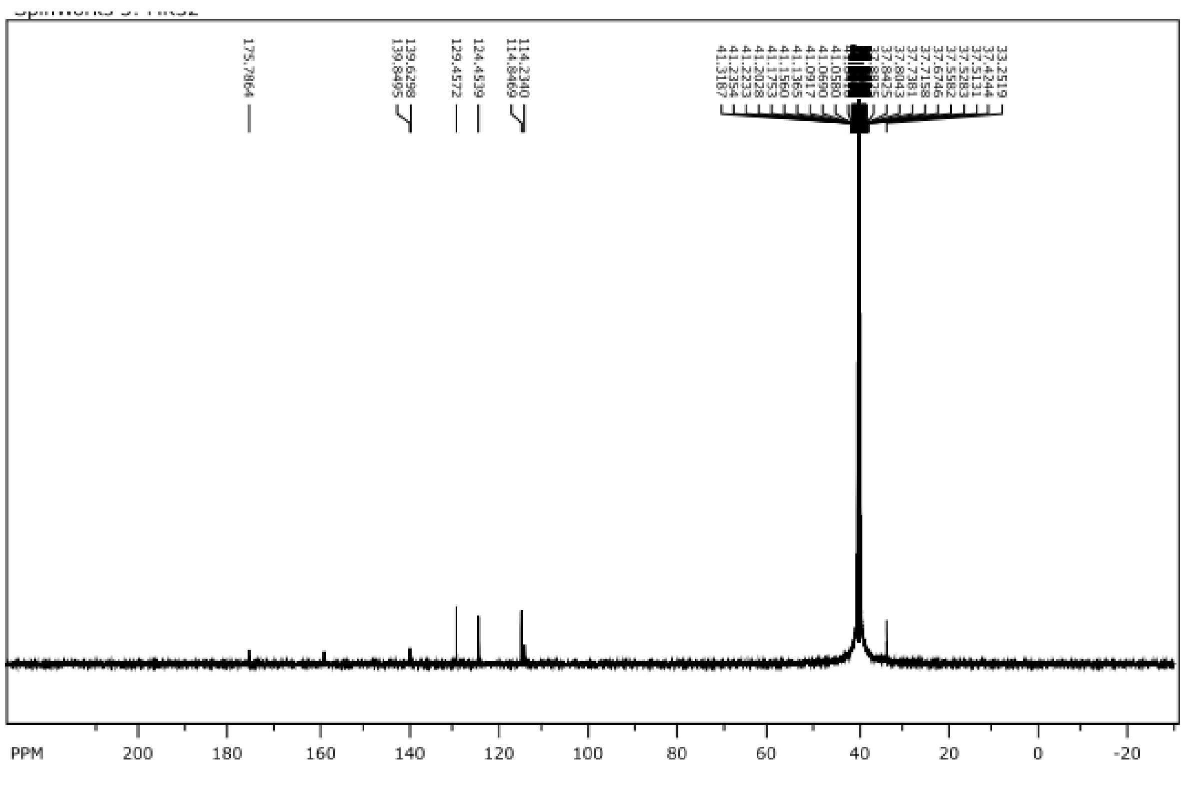

7-chloro-3-ethyl-2-mercaptoquinazolin-4(3H)-one (9b)

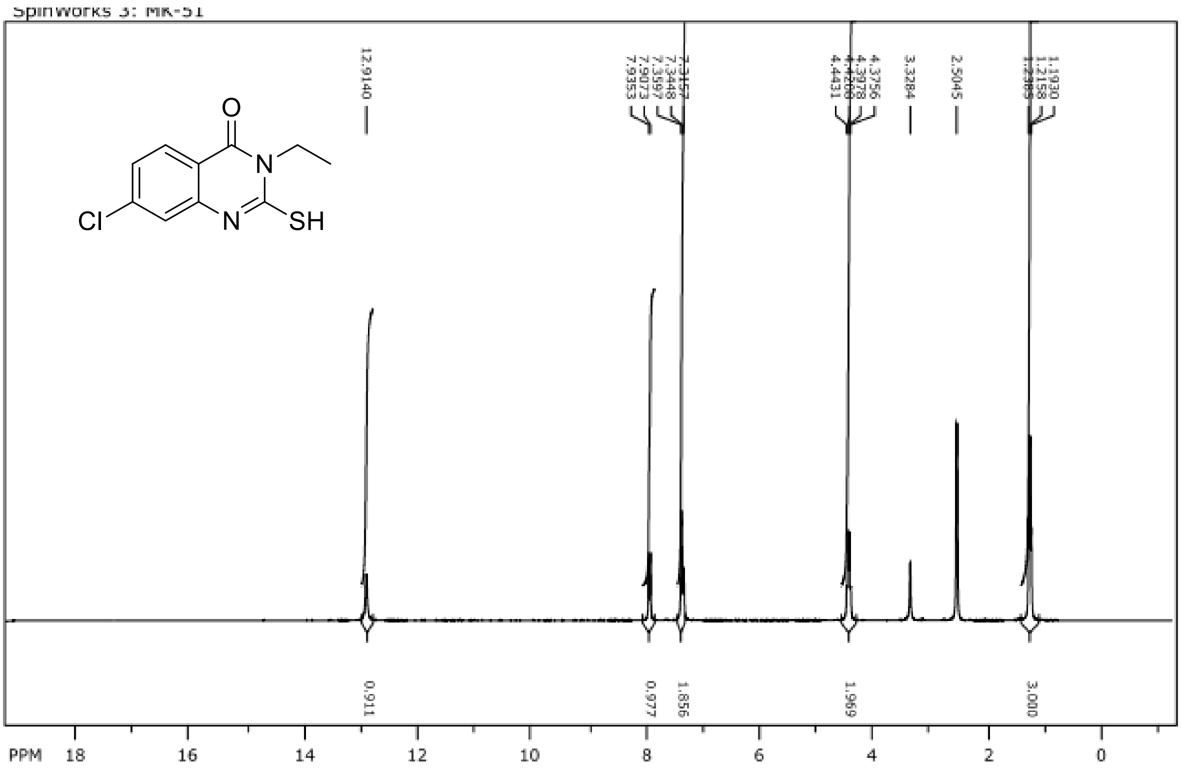

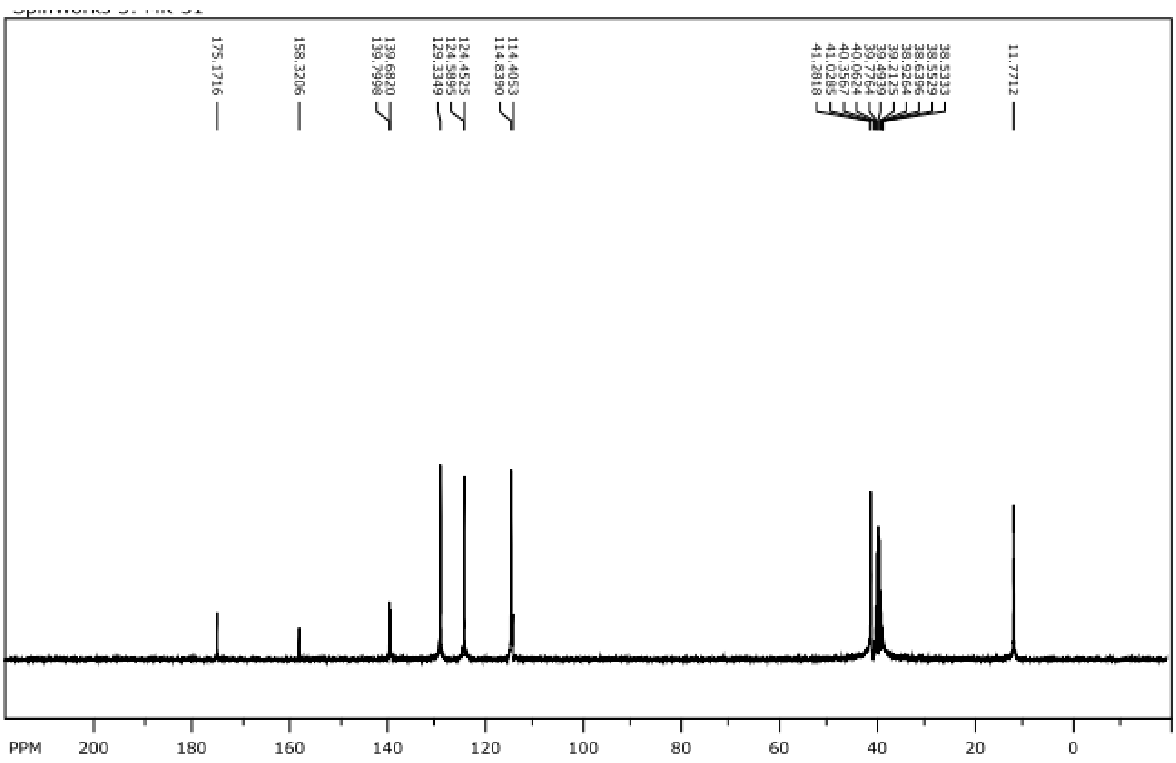

3-allyl-7-chloro-2-mercaptoquinazolin-4(3H)-one (9c)

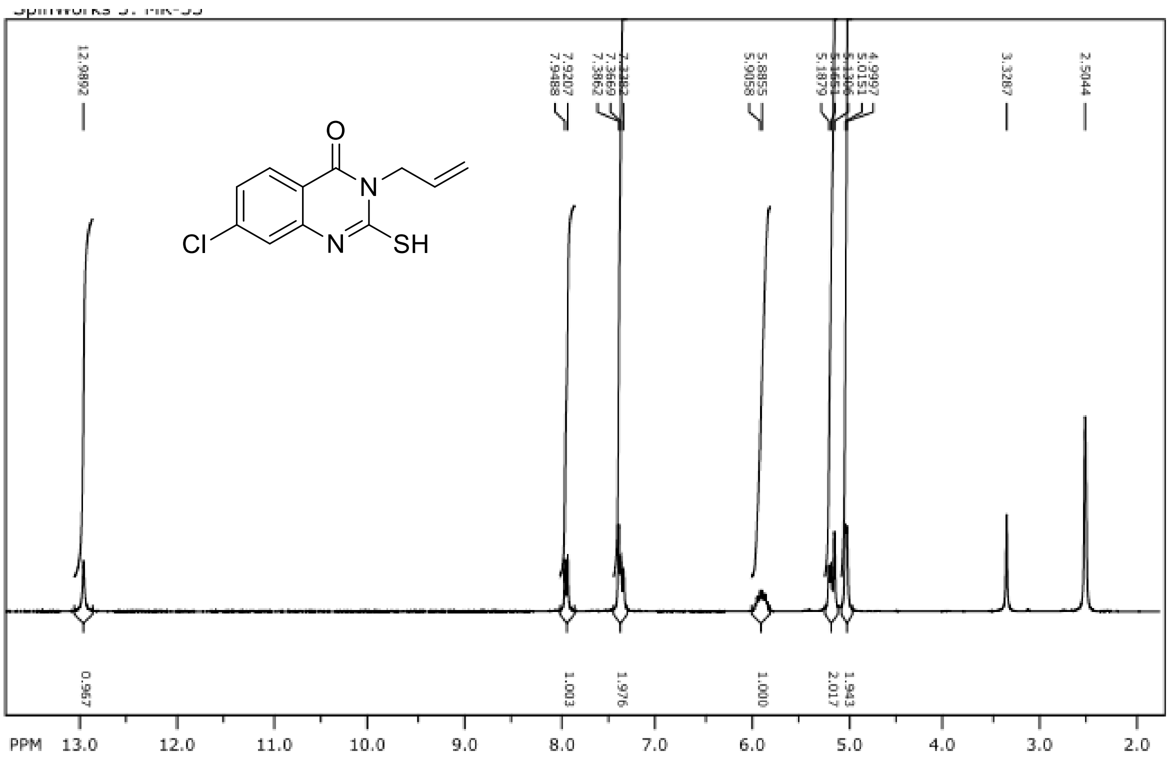

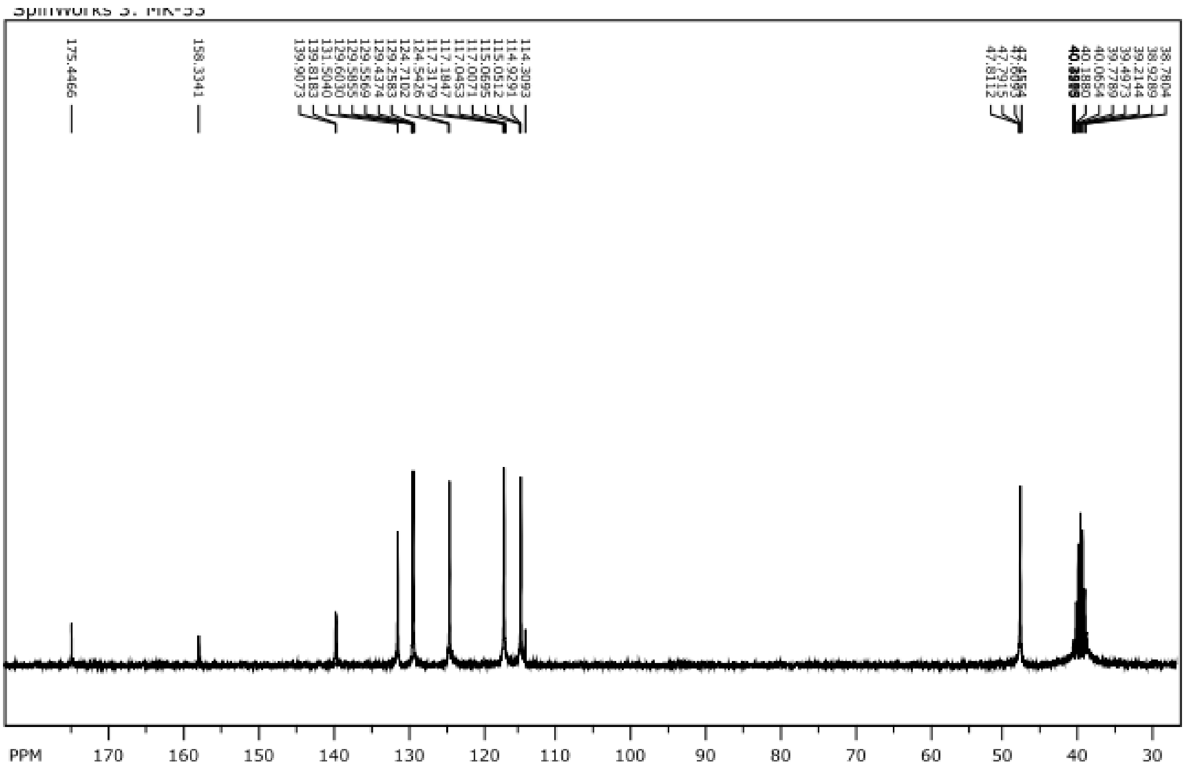

7-chloro-2-mercapto-3-phenylquinazolin-4(3H)-one (9d)

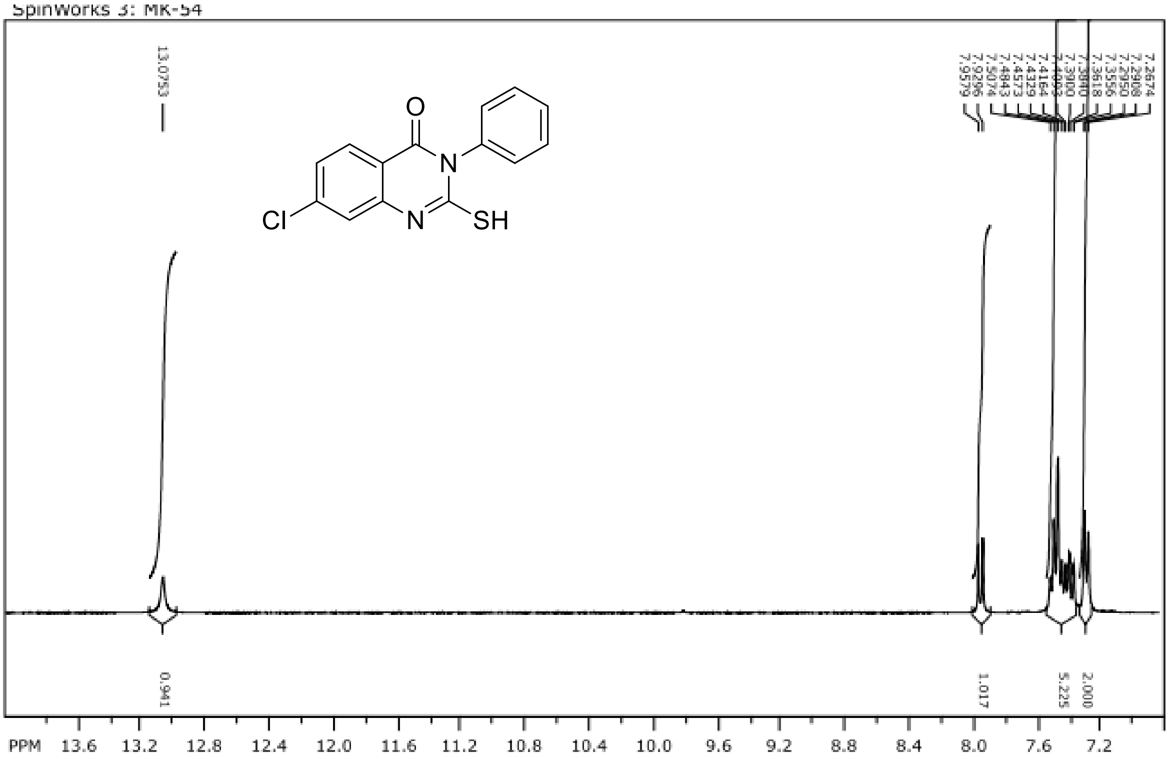

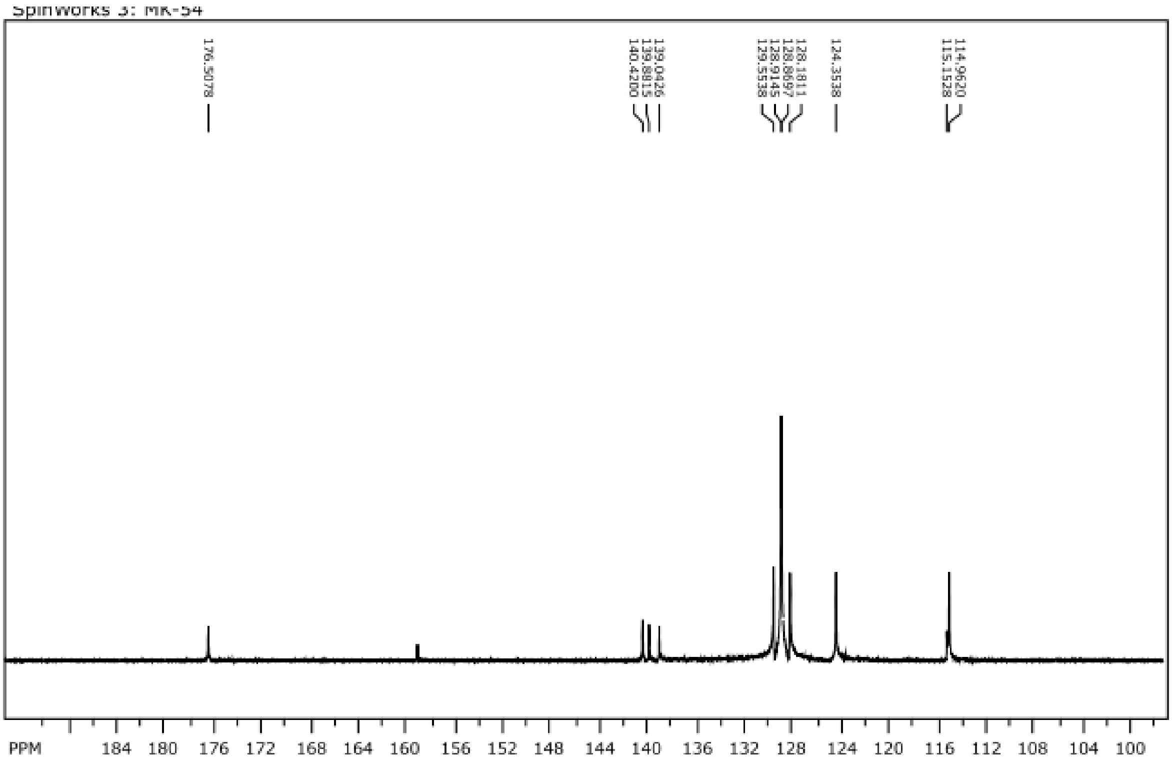

3-benzyl-7-chloro-2-mercaptoquinazolin-4(3H)-one (9e)

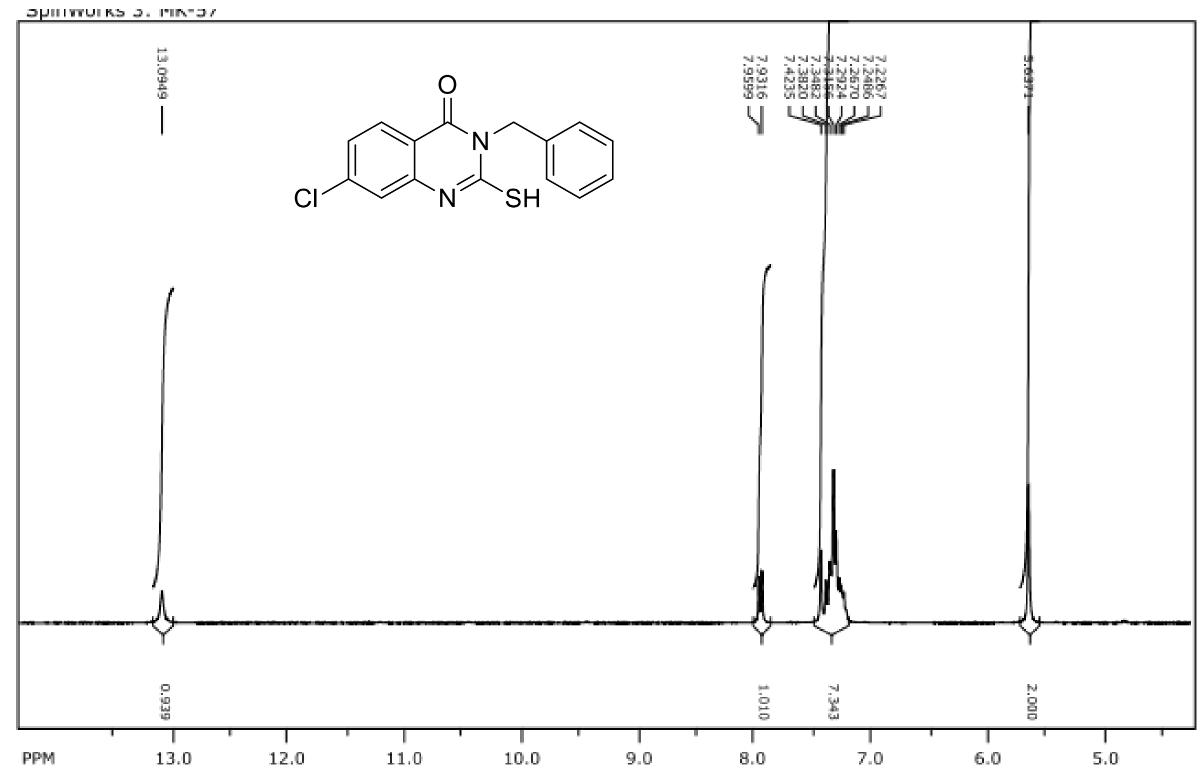

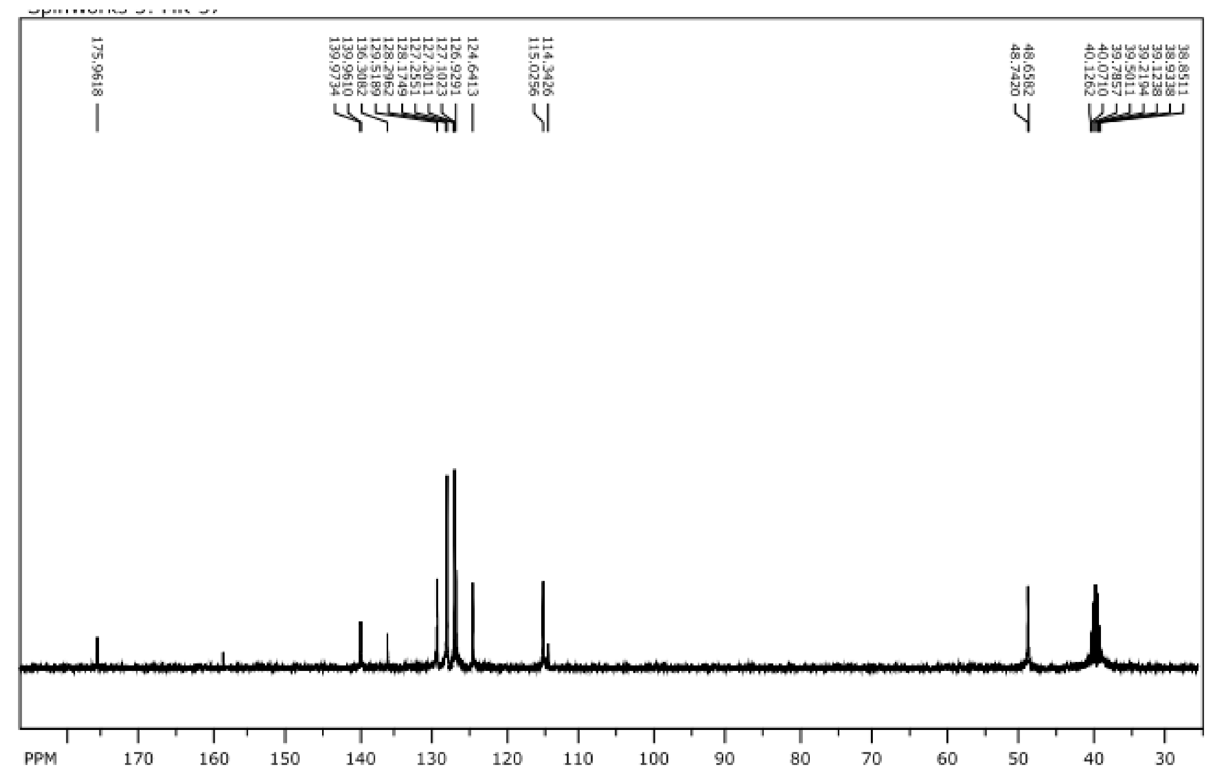

7-chloro-2-mercapto-3-(p-tolyl)quinazolin-4(3H)-one (9f)

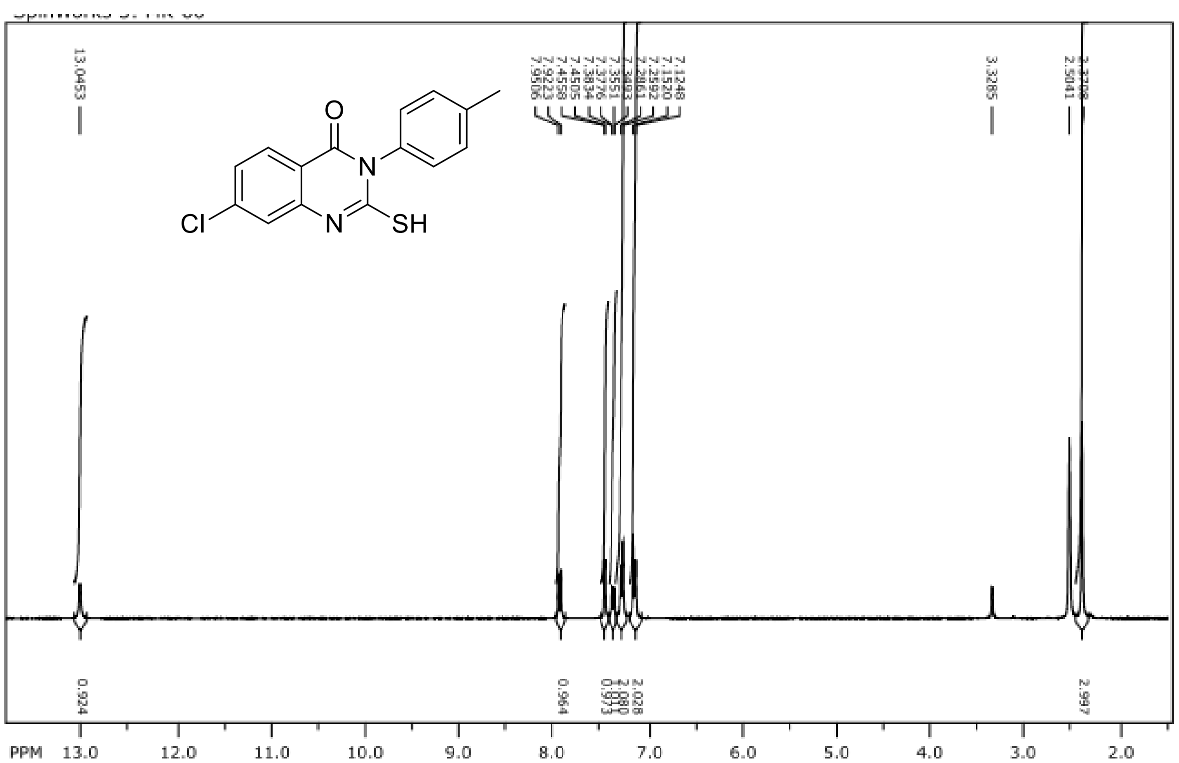

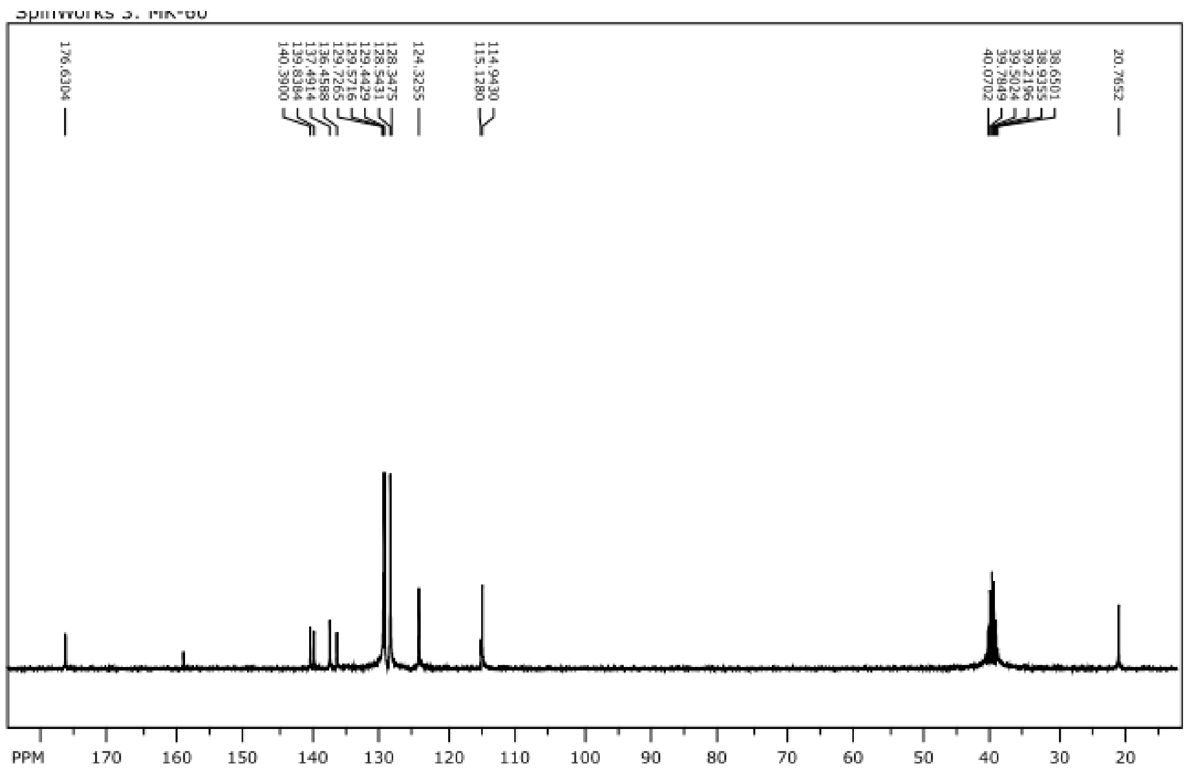

7-chloro-3-(4-fluorophenyl)-2-mercaptoquinazolin-4(3H)-one (9g)

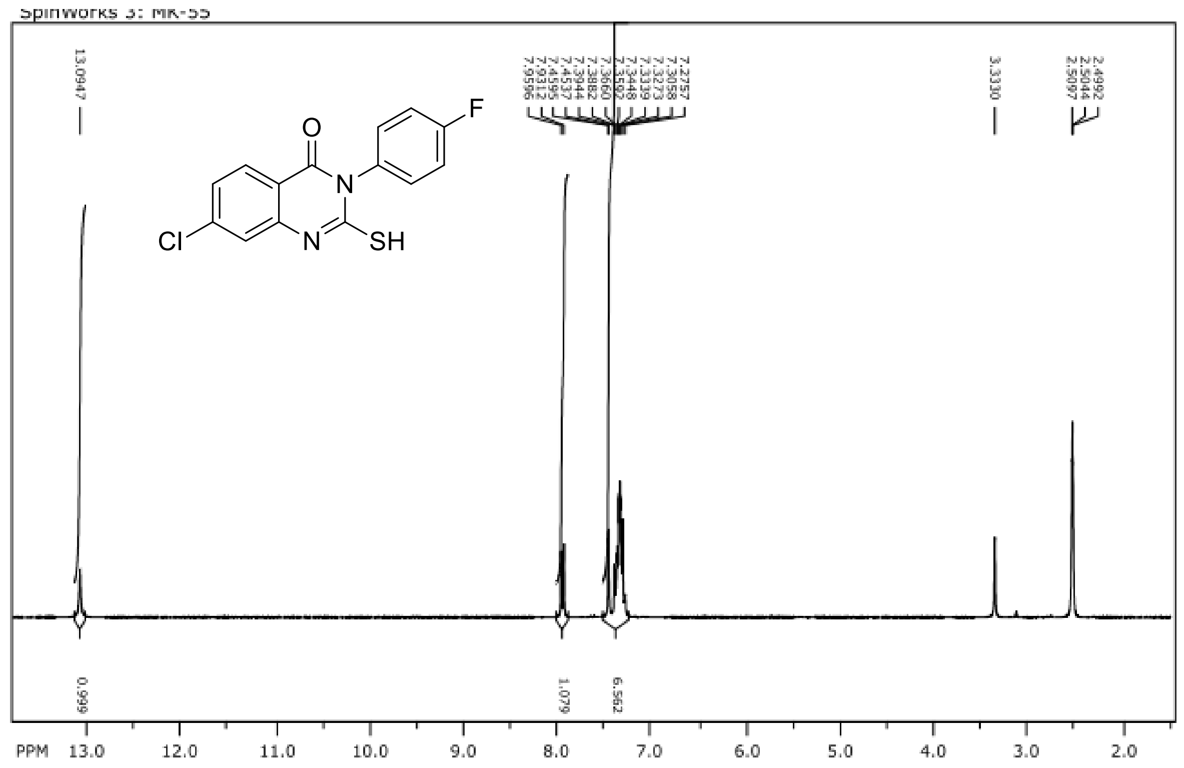

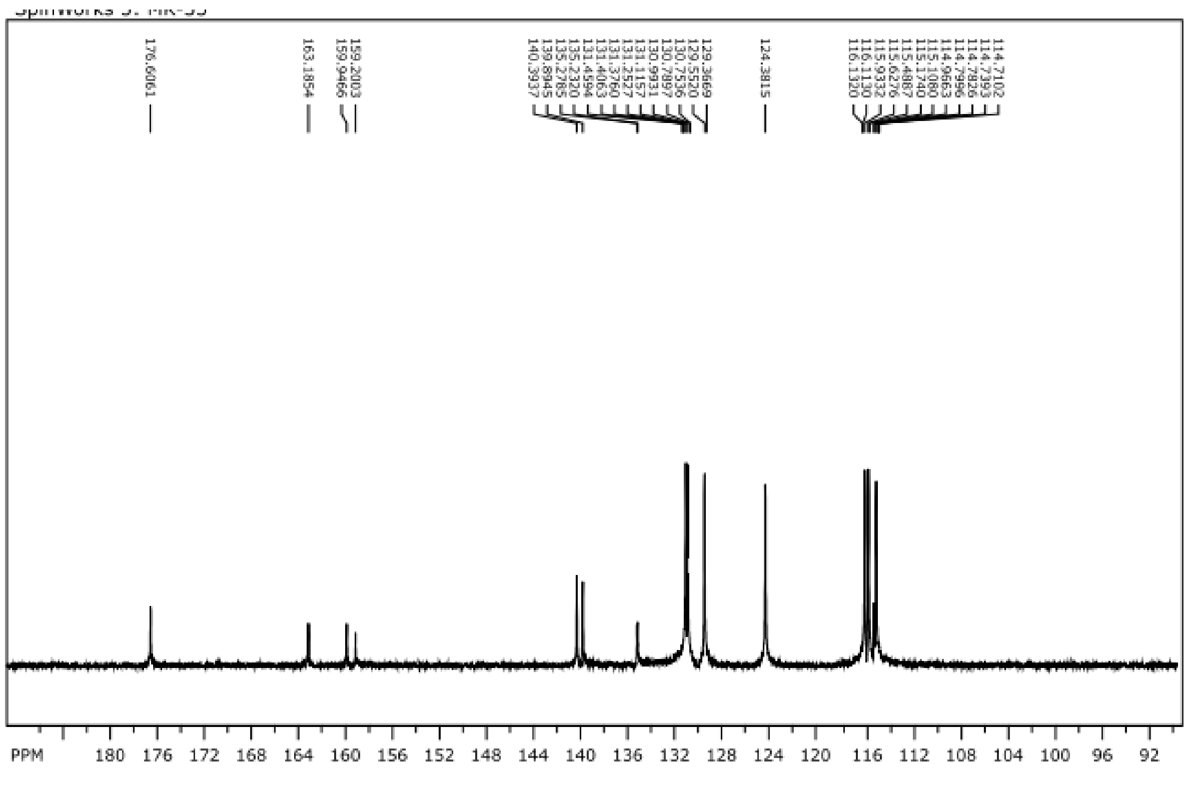

7-chloro-3-(4-chlorophenyl)-2-mercaptoquinazolin-4(3H)-one (9h)

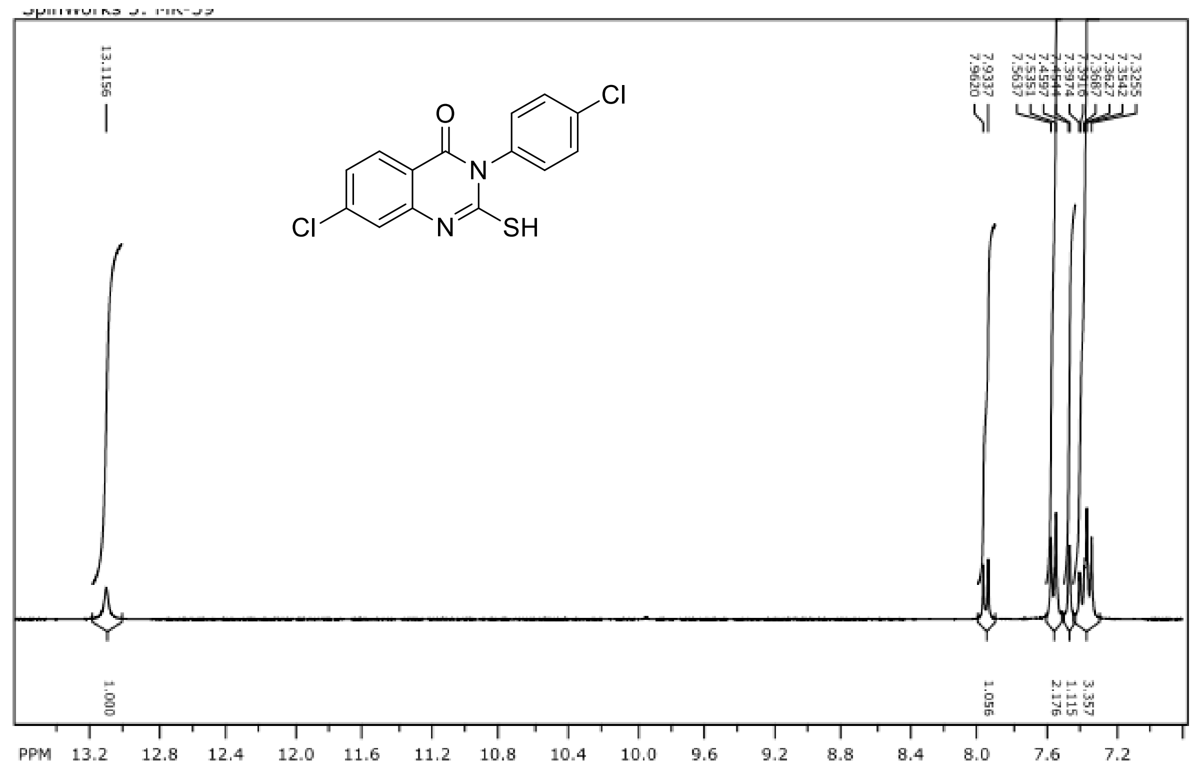

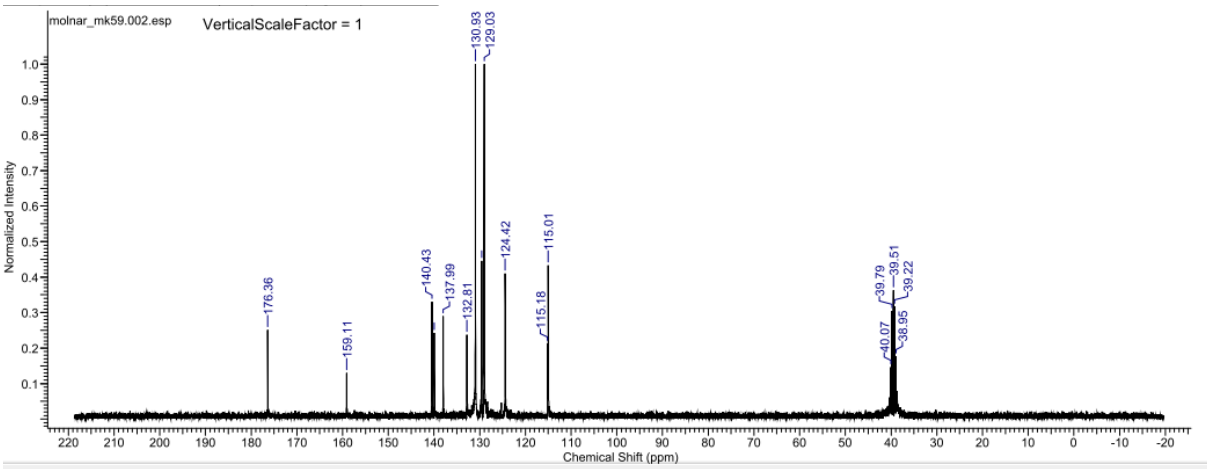

3-(4-bromophenyl)-7-chloro-2-mercaptoquinazolin-4(3H)-one (9i)

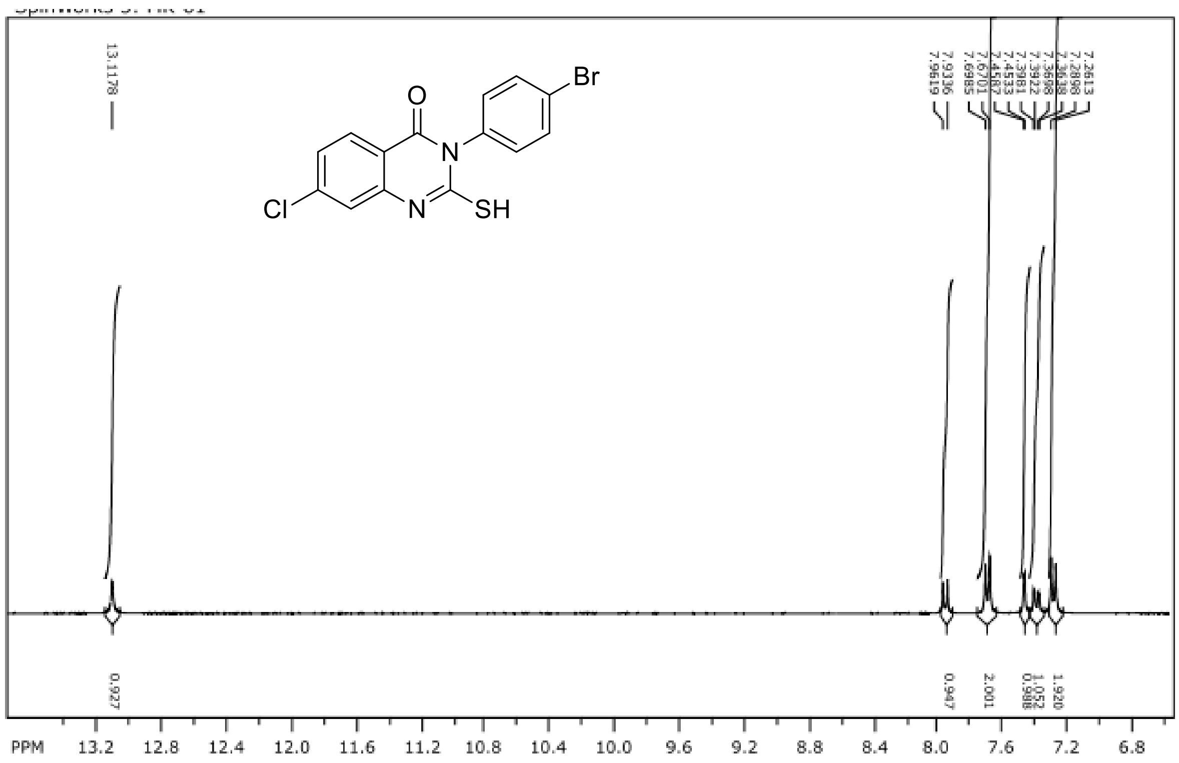

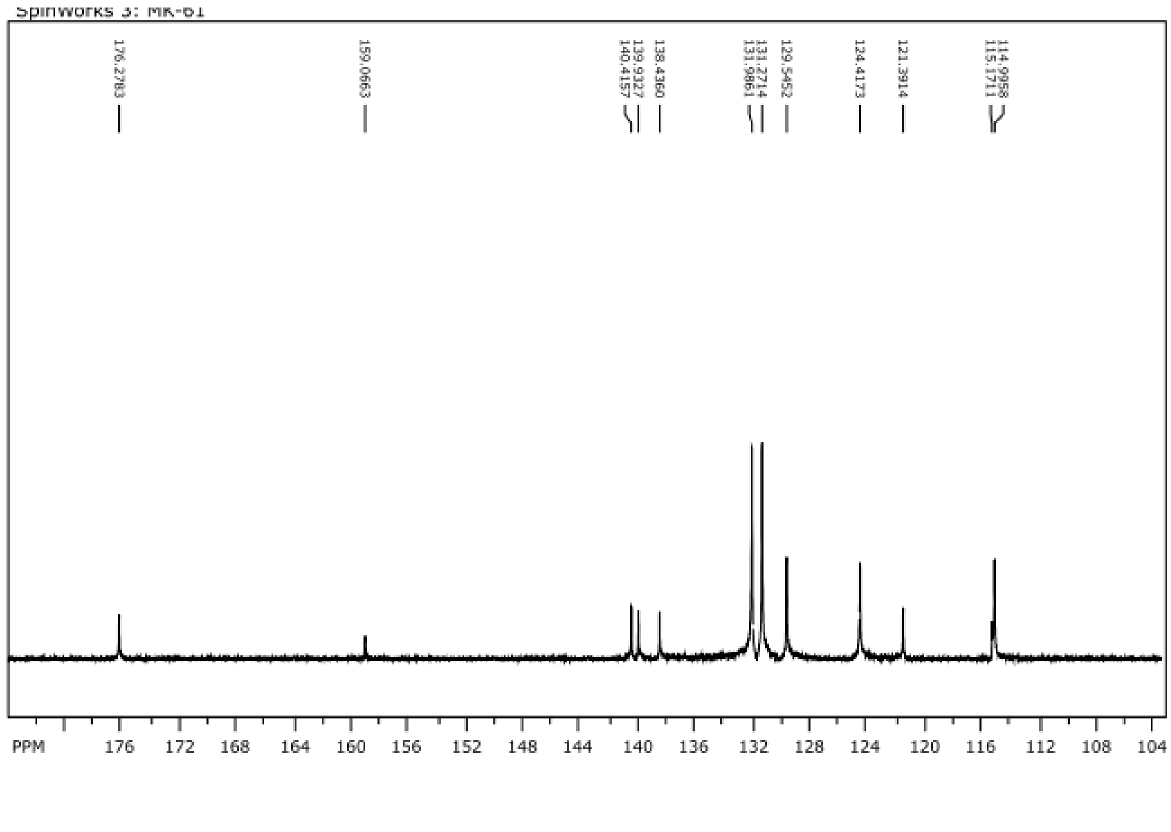

7-chloro-2-mercapto-3-(3-methoxyphenyl)quinazolin-4(3H)-one (9j)

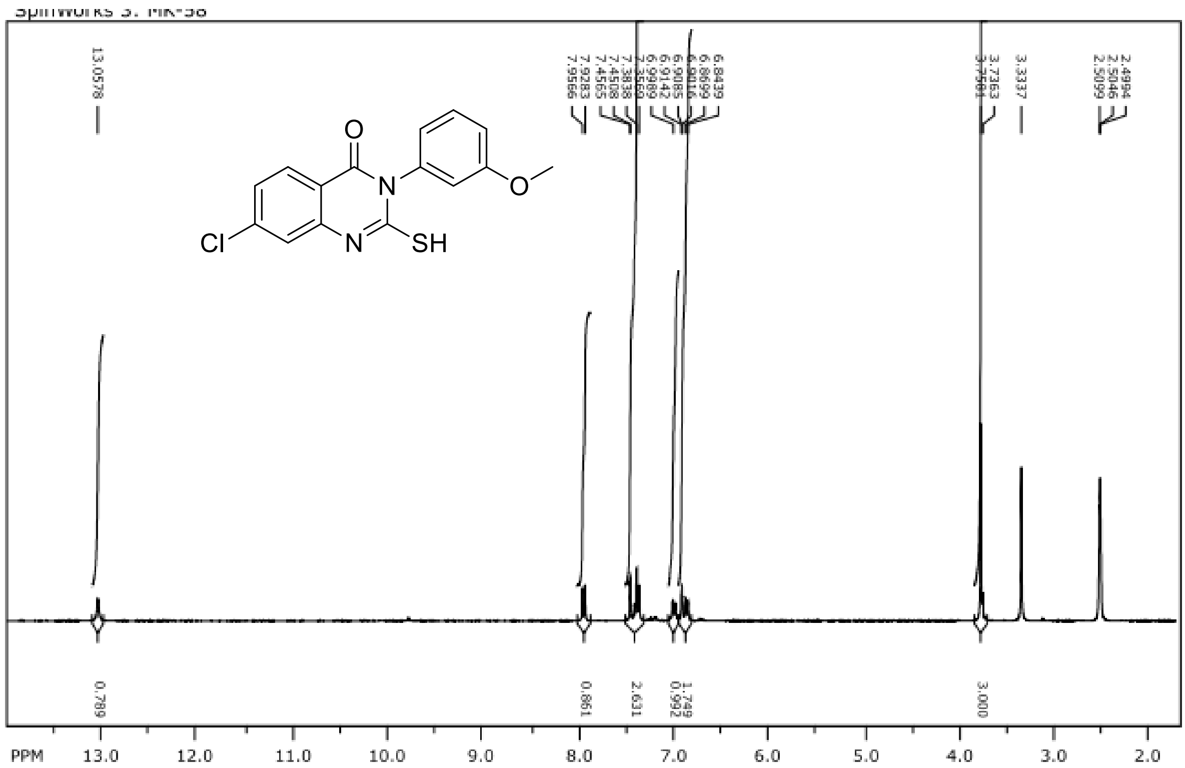

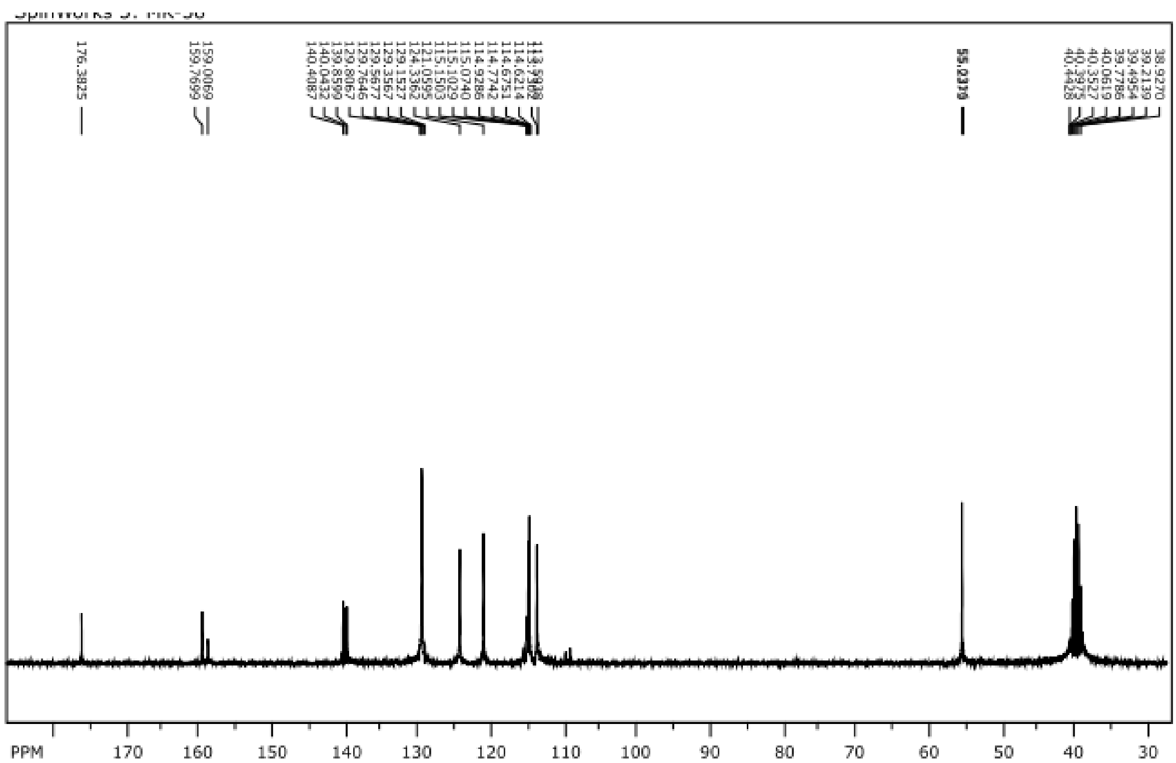

7-chloro-3-(3-chlorophenyl)-2-mercaptoquinazolin-4(3H)-one (9k)

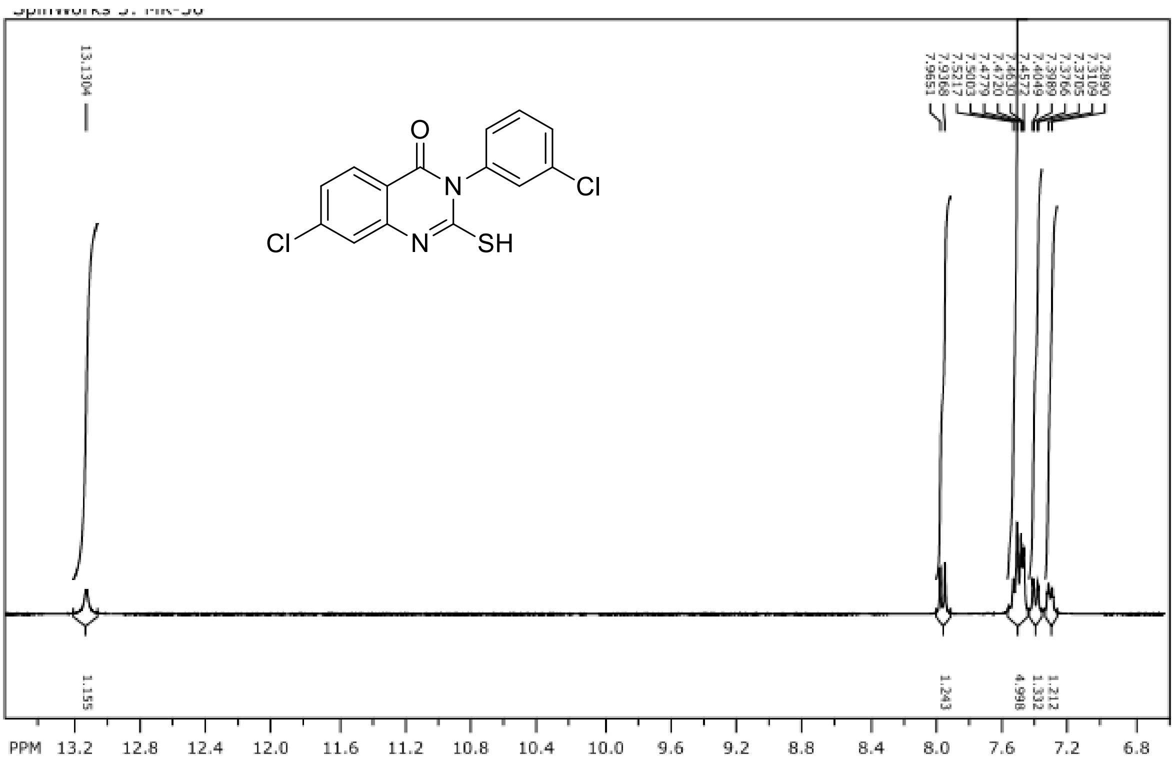

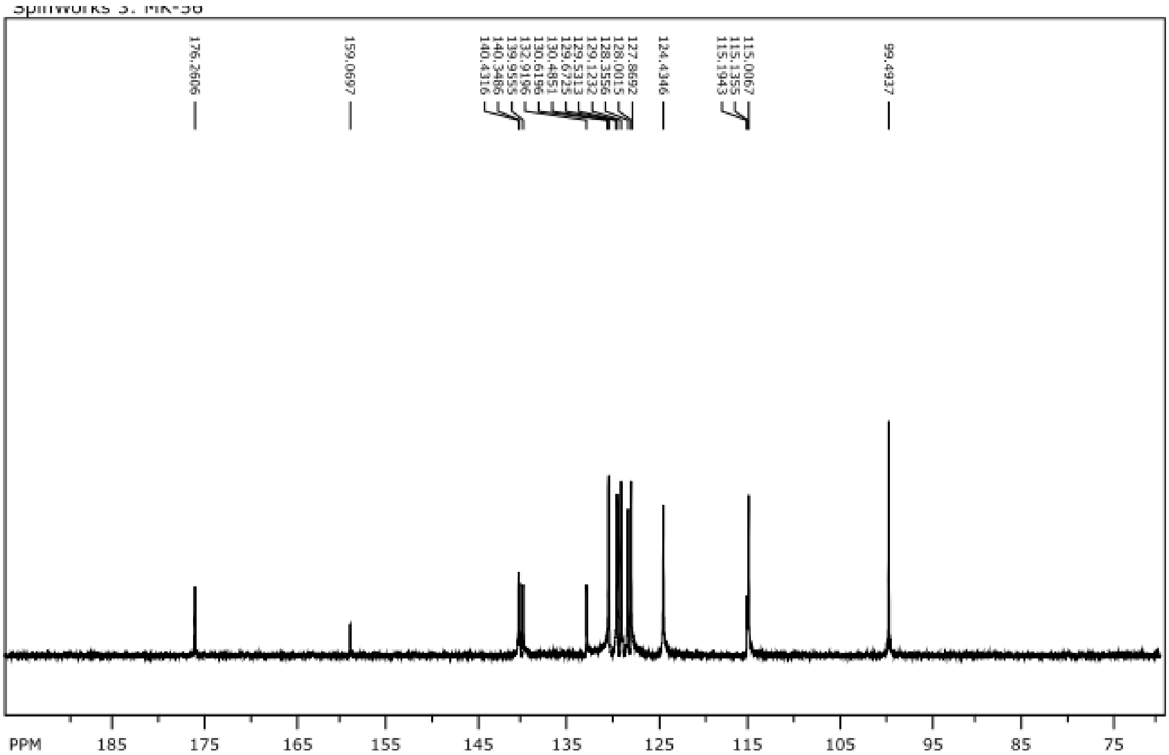

6,8-dichloro-2-mercapto-3-methylquinazolin-4(3H)-one (10a)

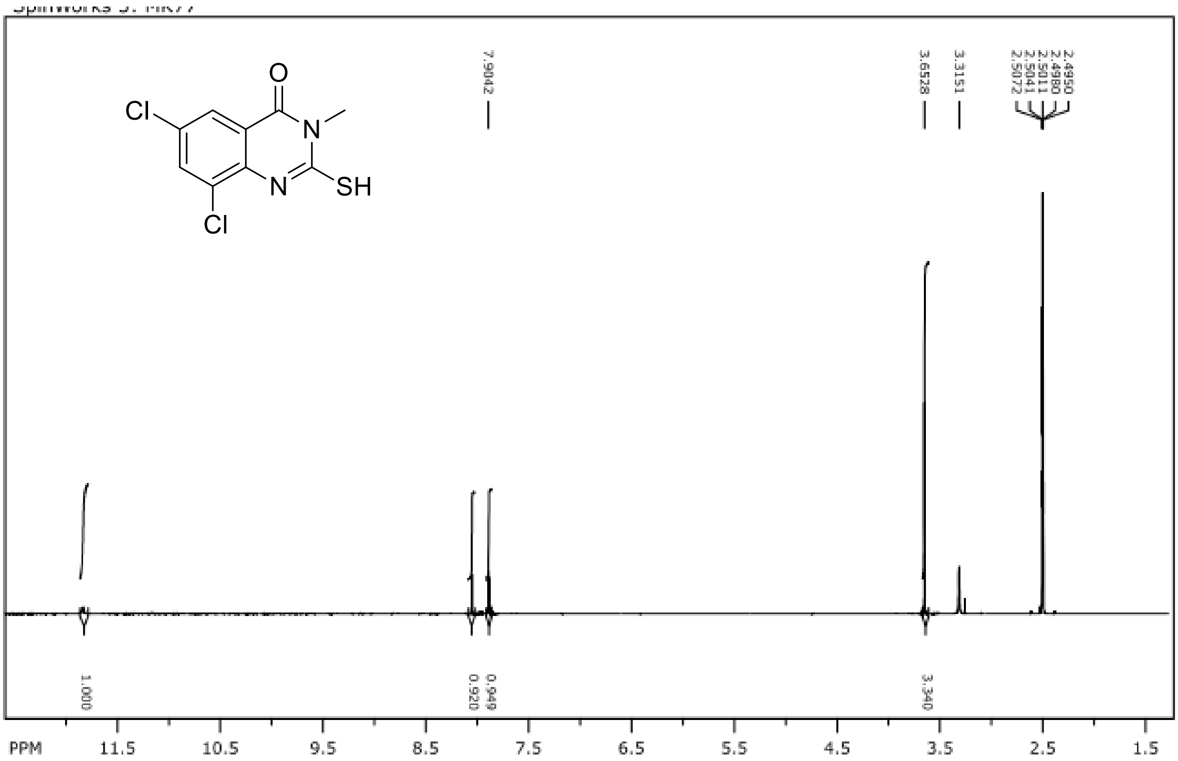

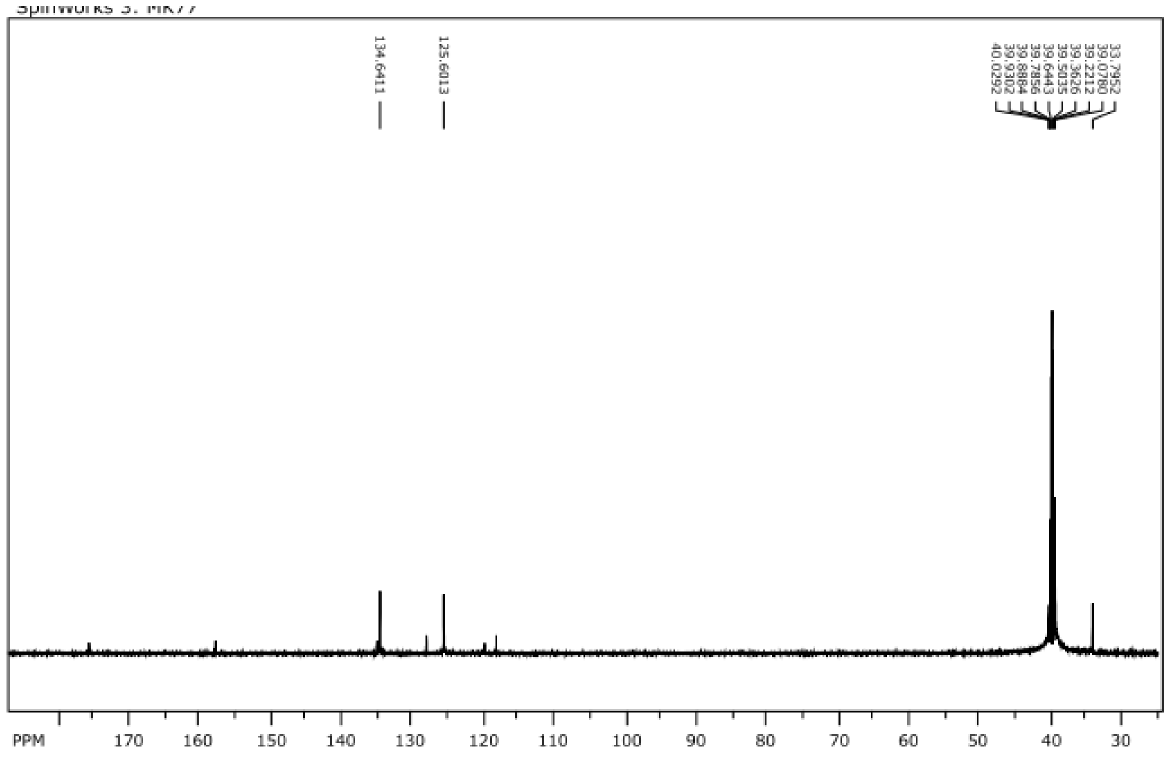

6,8-dichloro-3-ethyl-2-mercaptoquinazolin-4(3H)-one (10b)

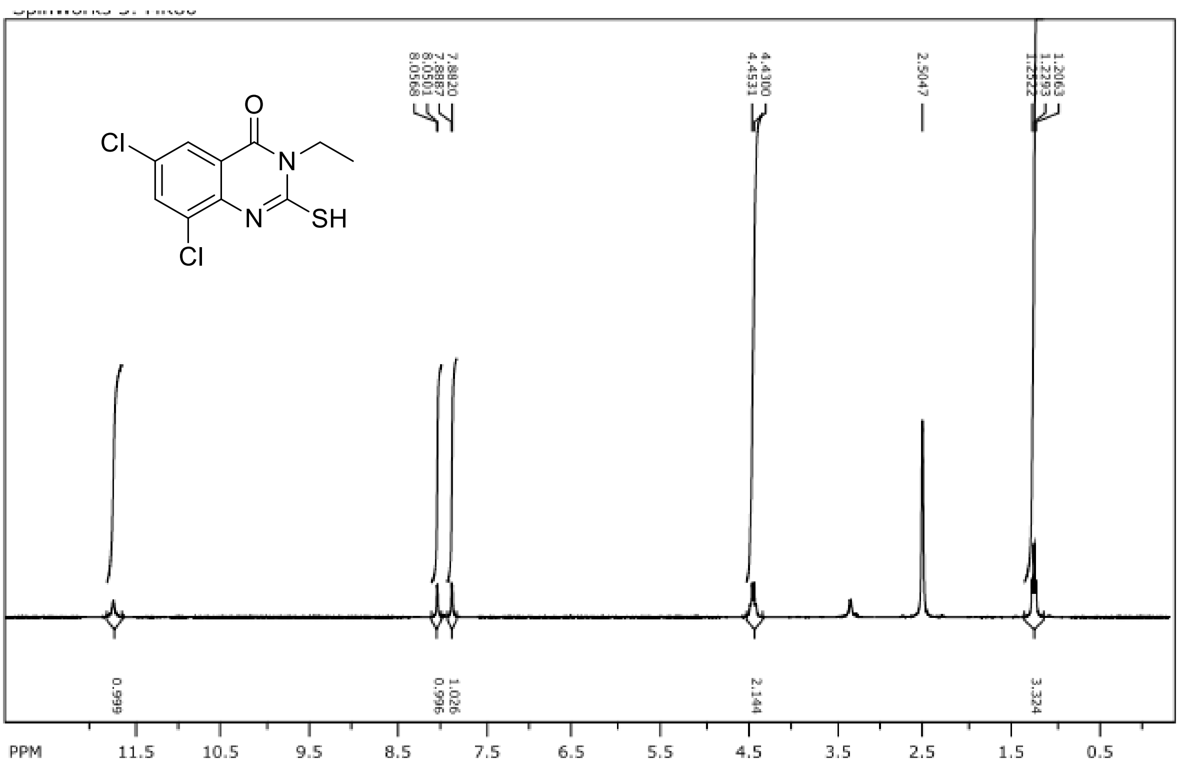

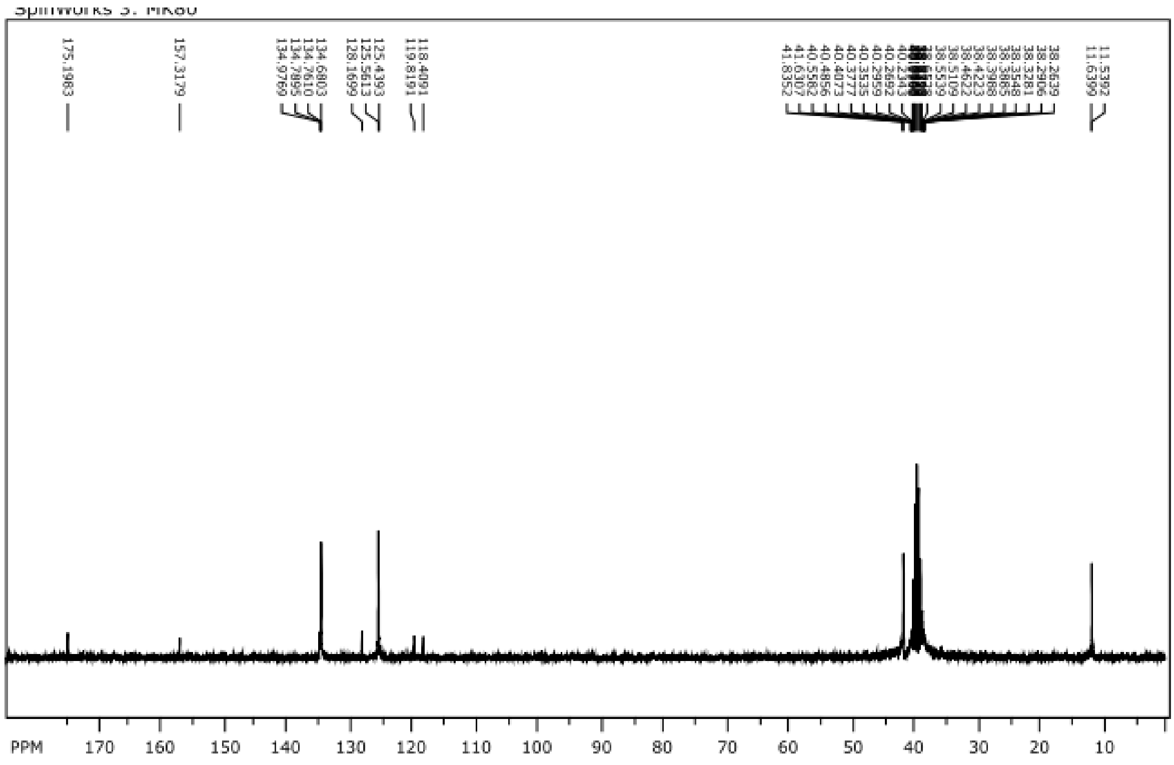

3-allyl-6,8-dichloro-2-mercaptoquinazolin-4(3H)-one (10c)

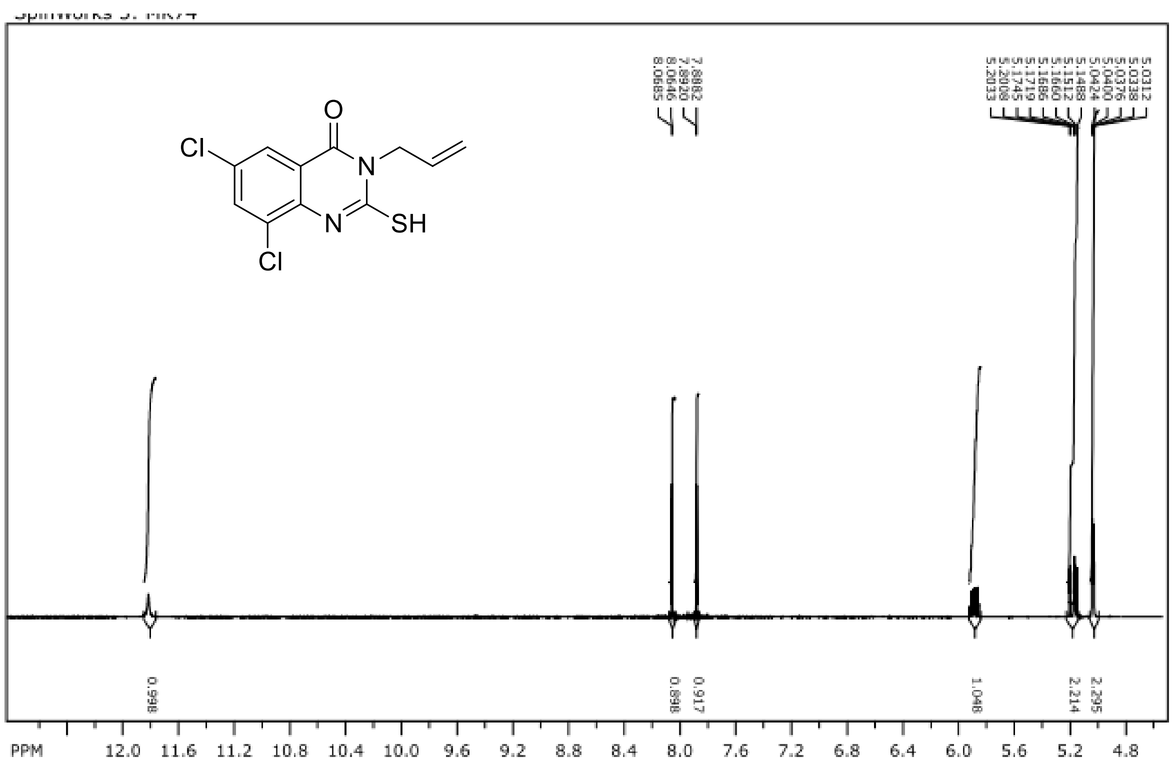

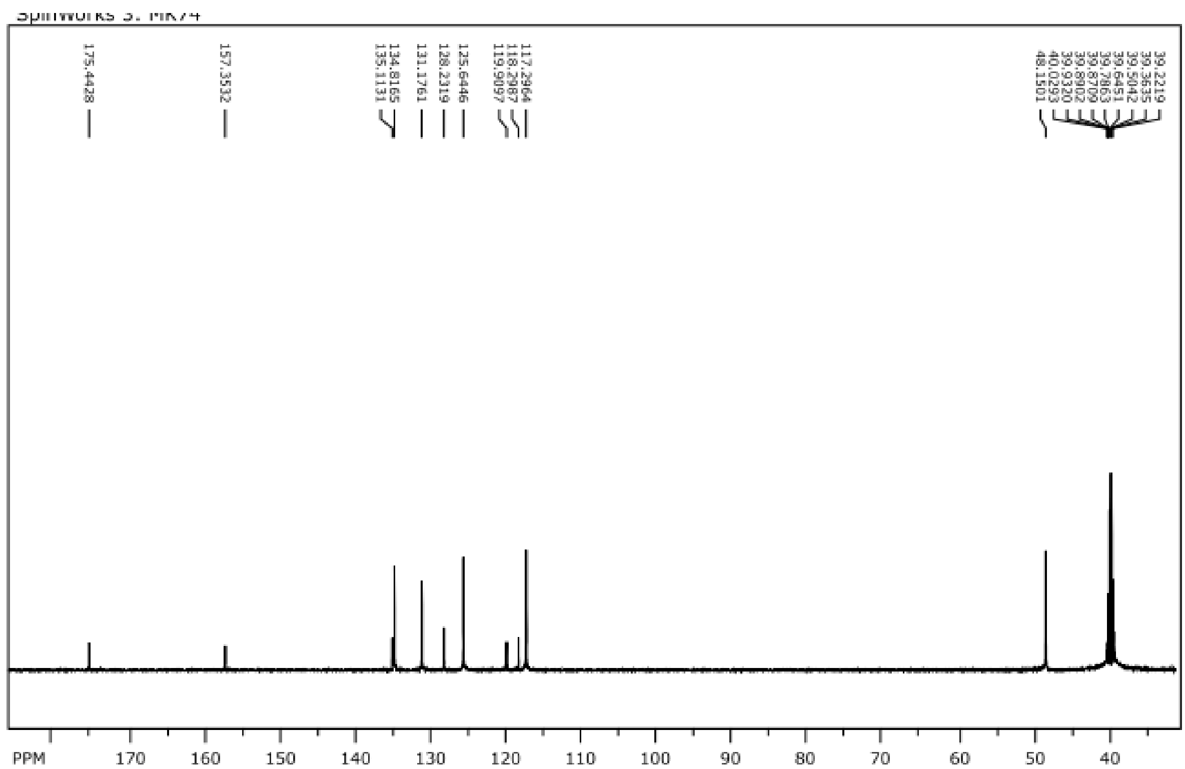

6,8-dichloro-2-mercapto-3-phenylquinazolin-4(3H)-one (10d)

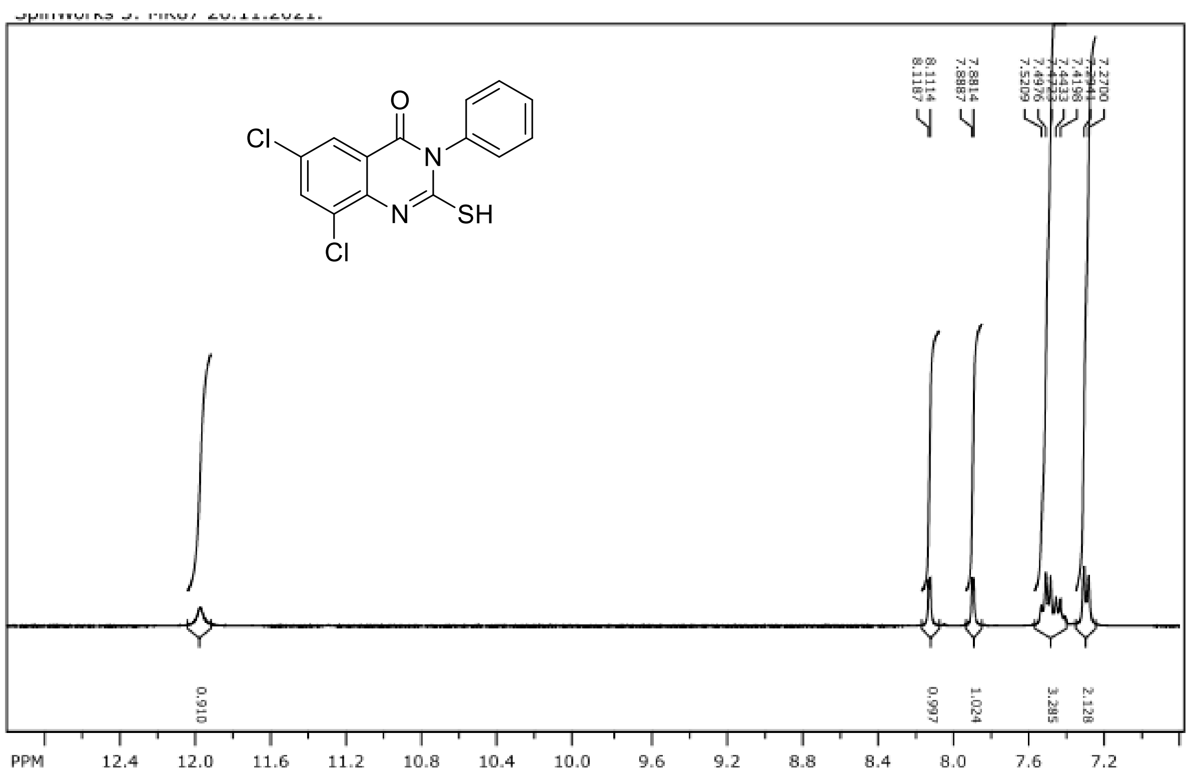

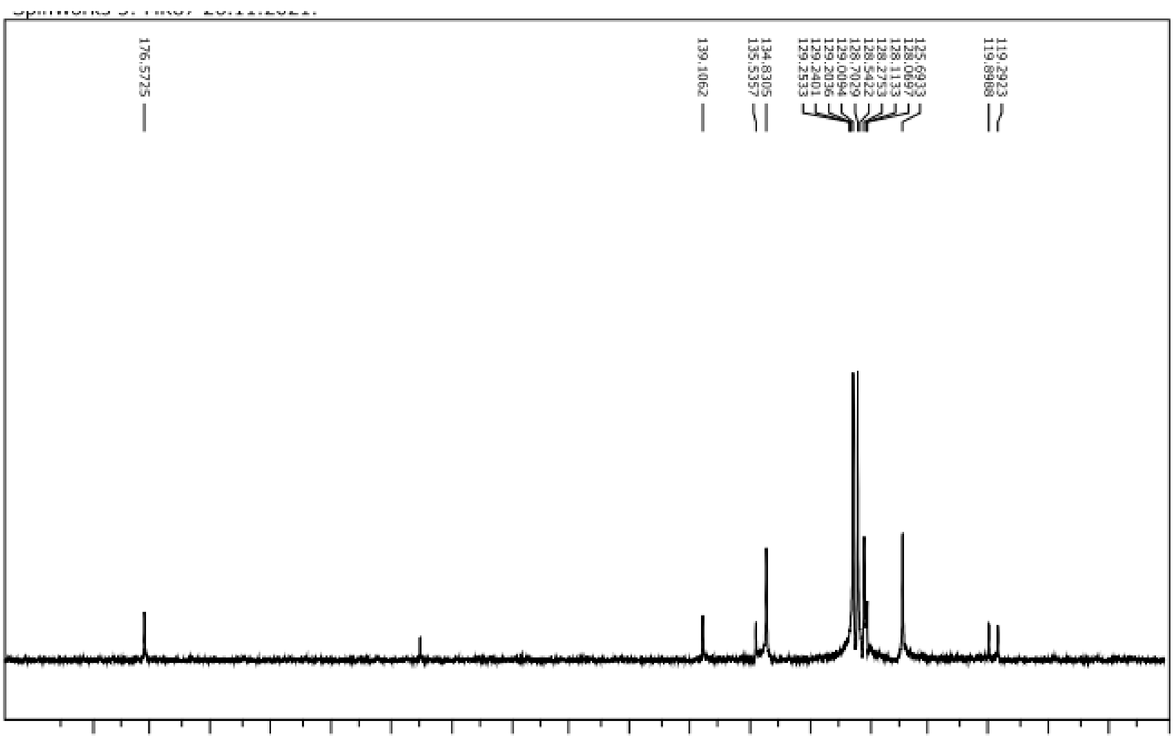

3-benzyl-6,8-dichloro-2-mercaptoquinazolin-4(3H)-one (10e)

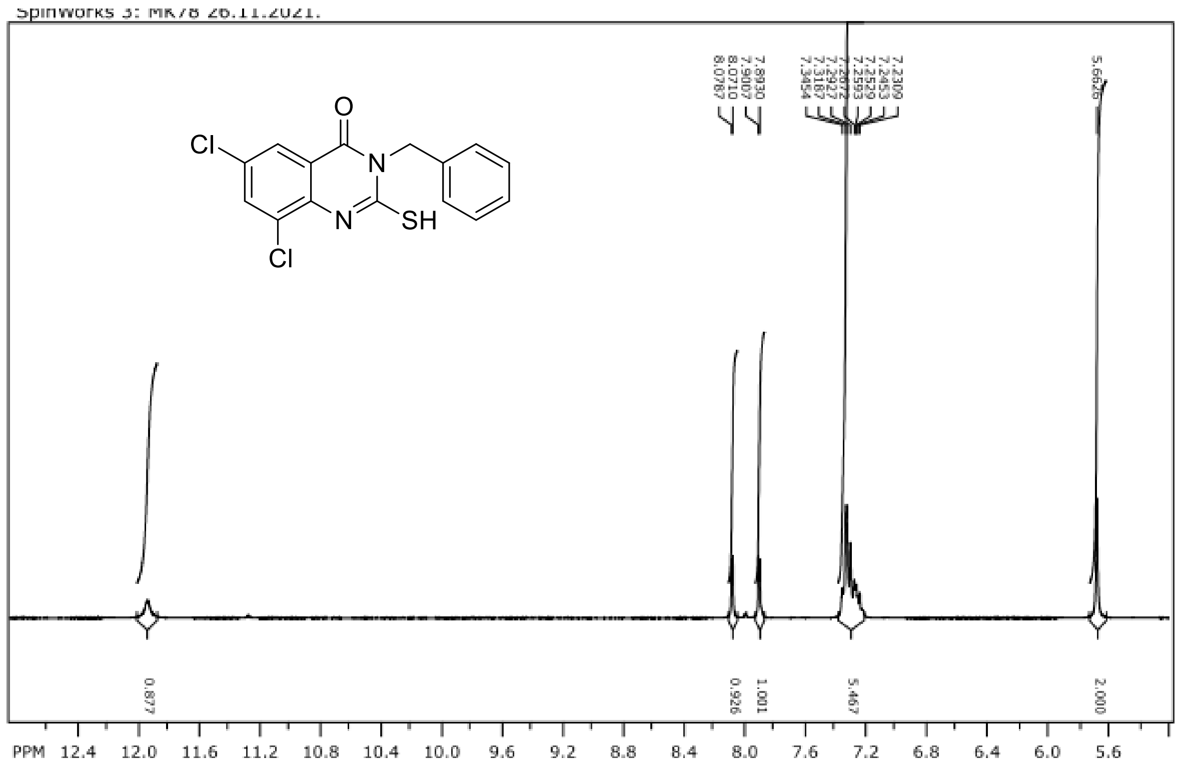

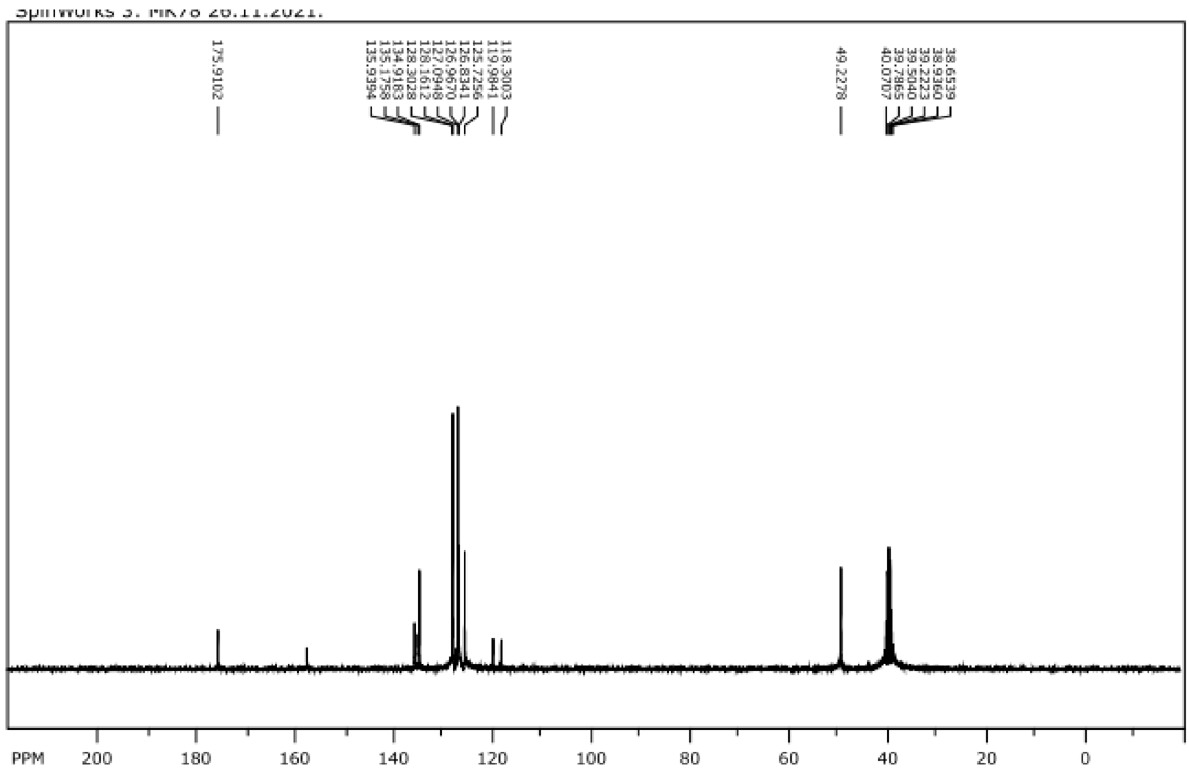

6,8-dichloro-2-mercapto-3-(p-tolyl)quinazolin-4(3H)-one (10f)

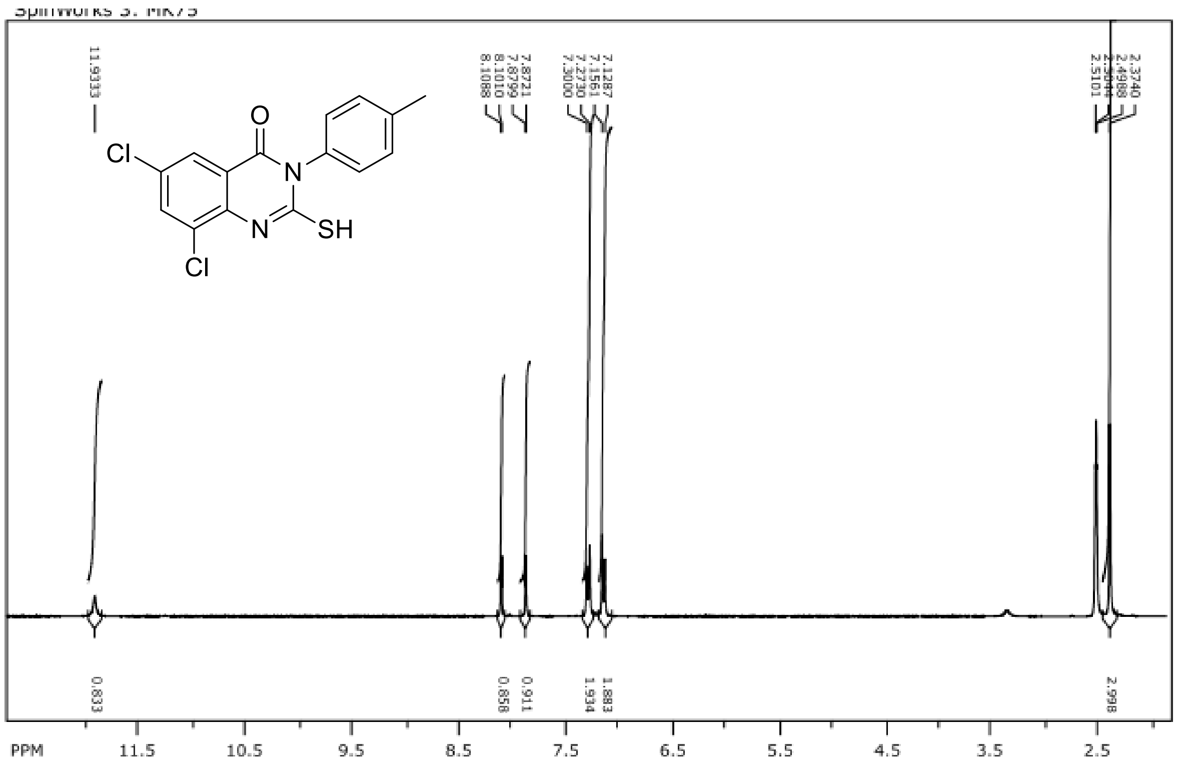

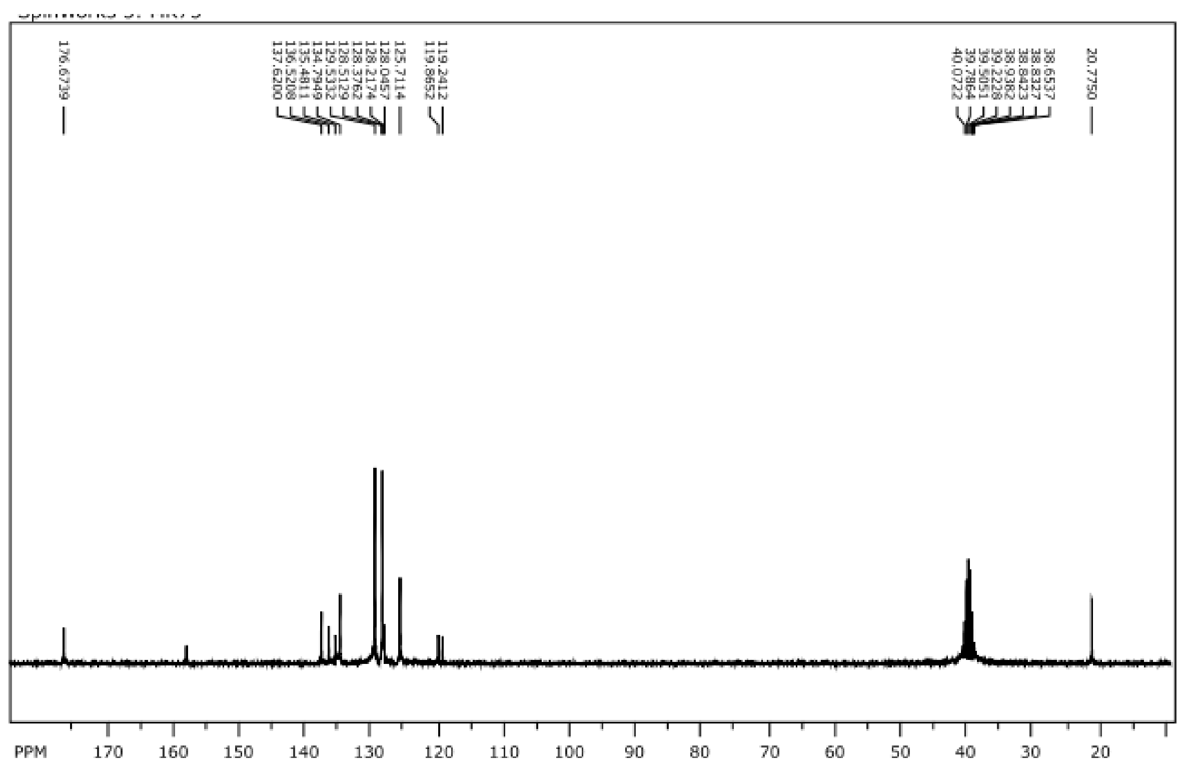

6,8-dichloro-3-(4-fluorophenyl)-2-mercaptoquinazolin-4(3H)-one (10g)

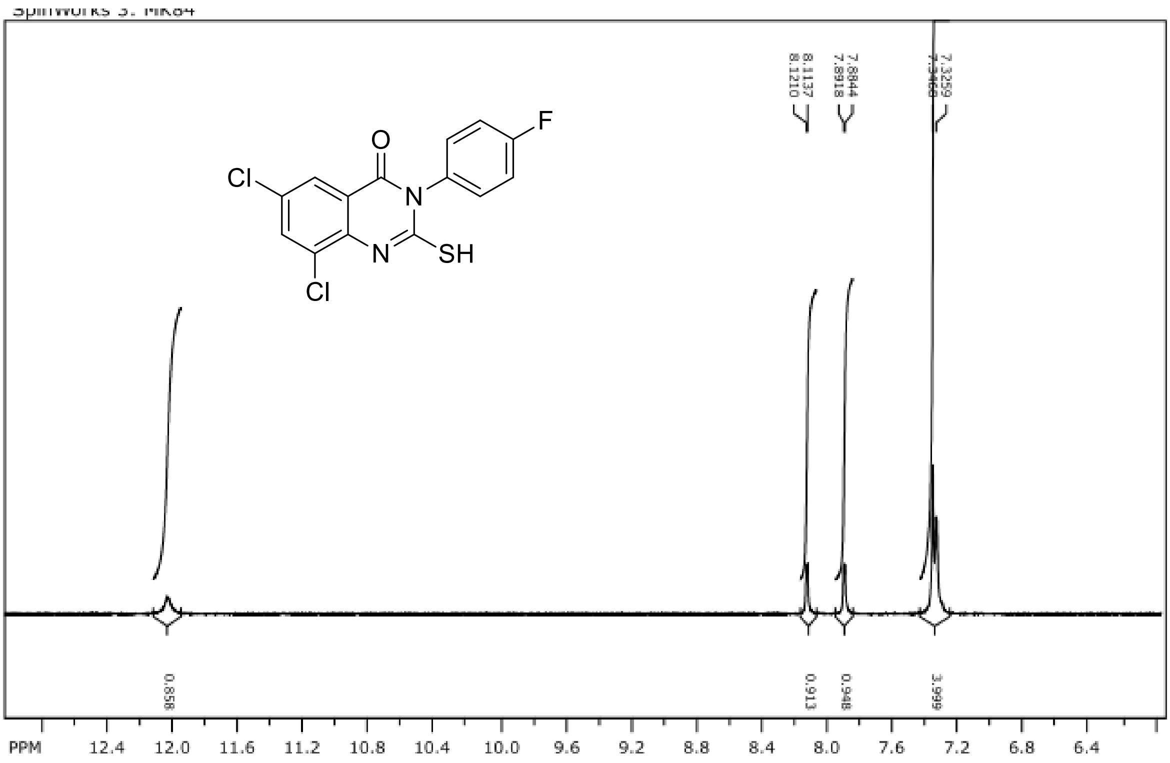

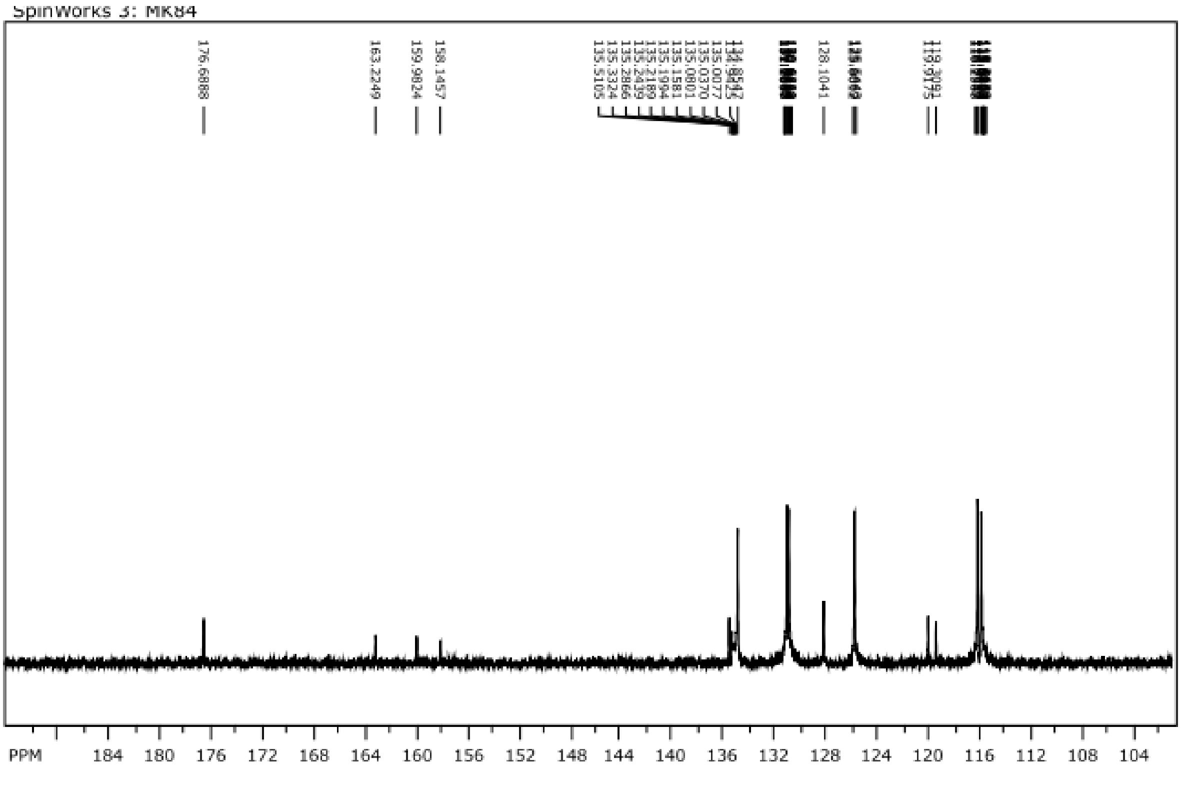

6,8-dichloro-3-(4-chlorophenyl)-2-mercaptoquinazolin-4(3H)-one (10h)

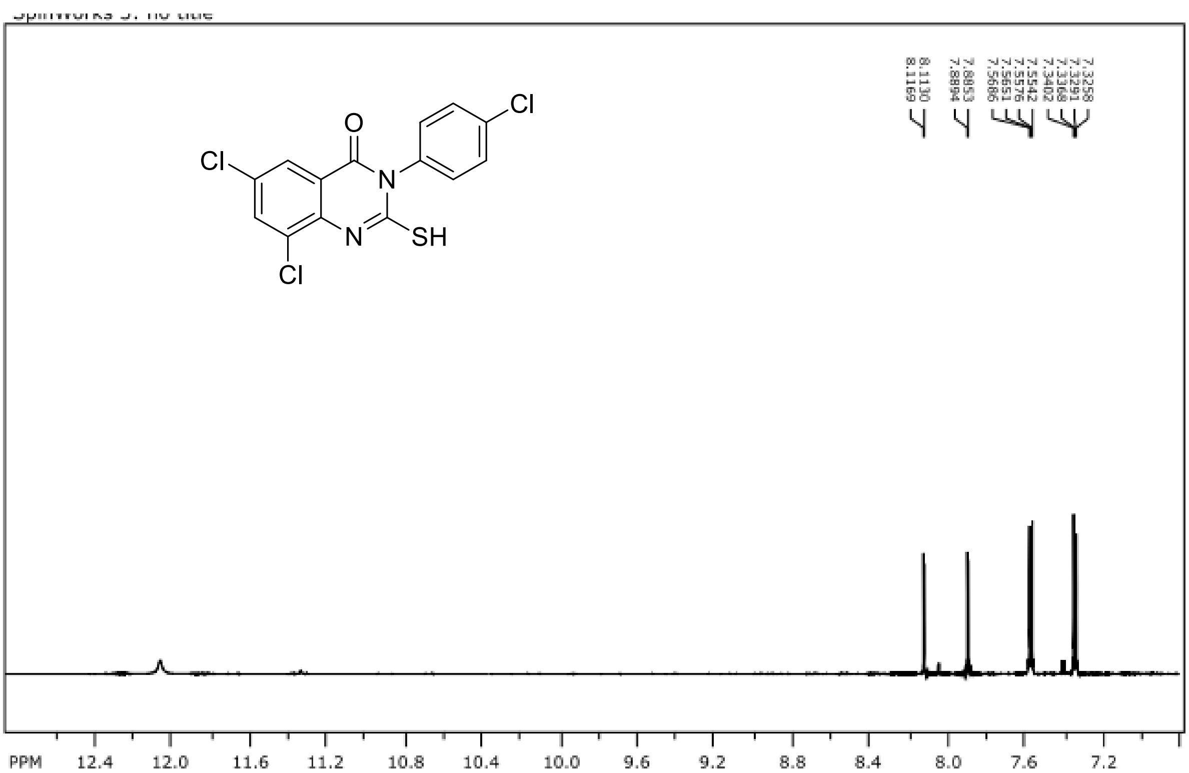

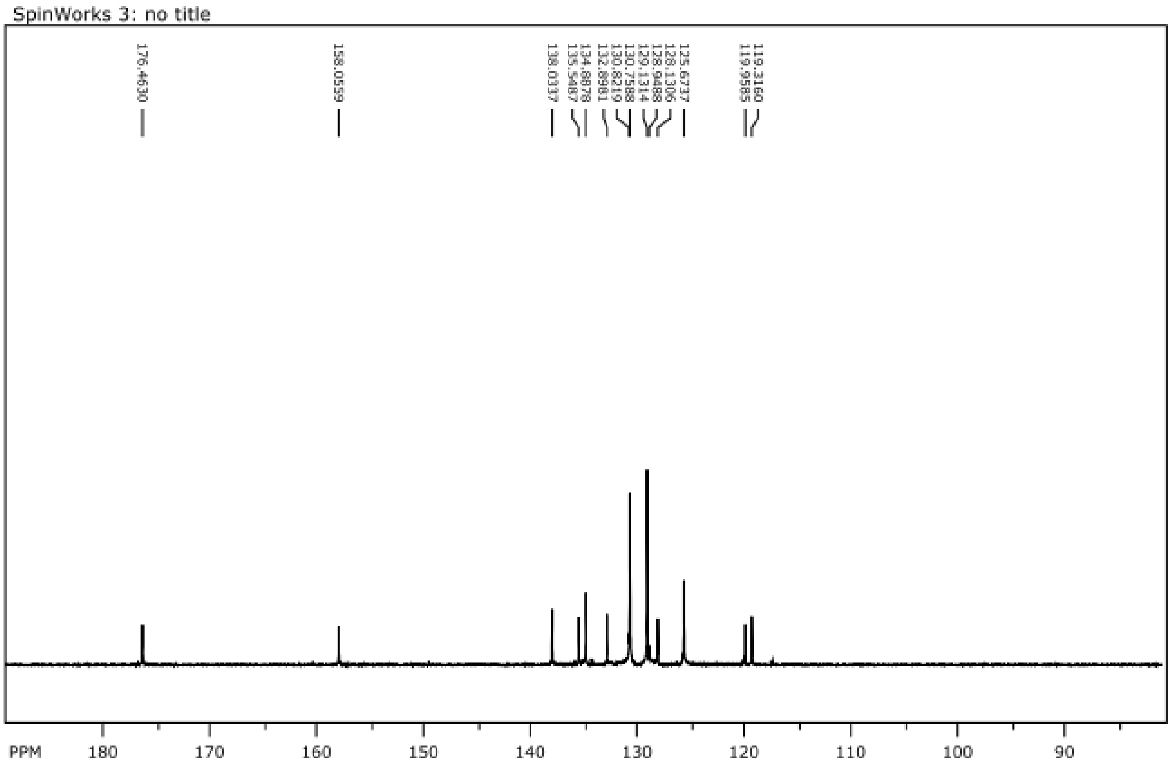

3-(4-bromophenyl)-6,8-dichloro-2-mercaptoquinazolin-4(3H)-one (10i)

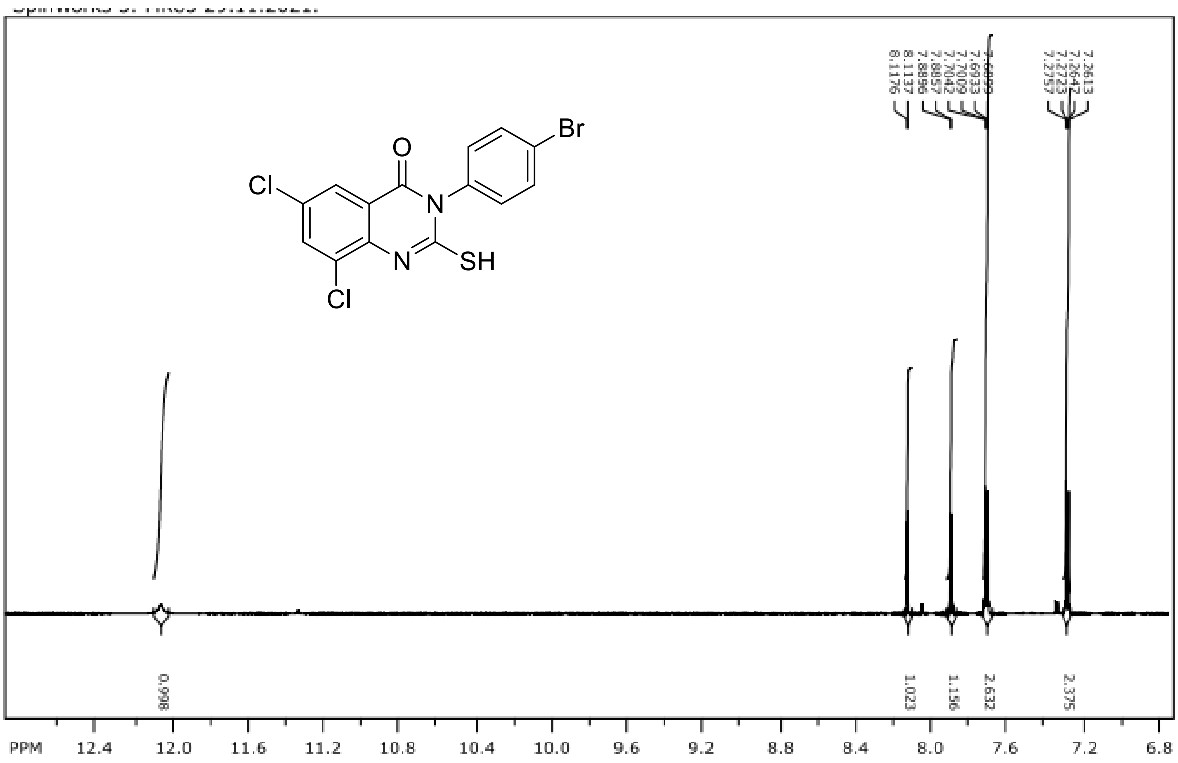

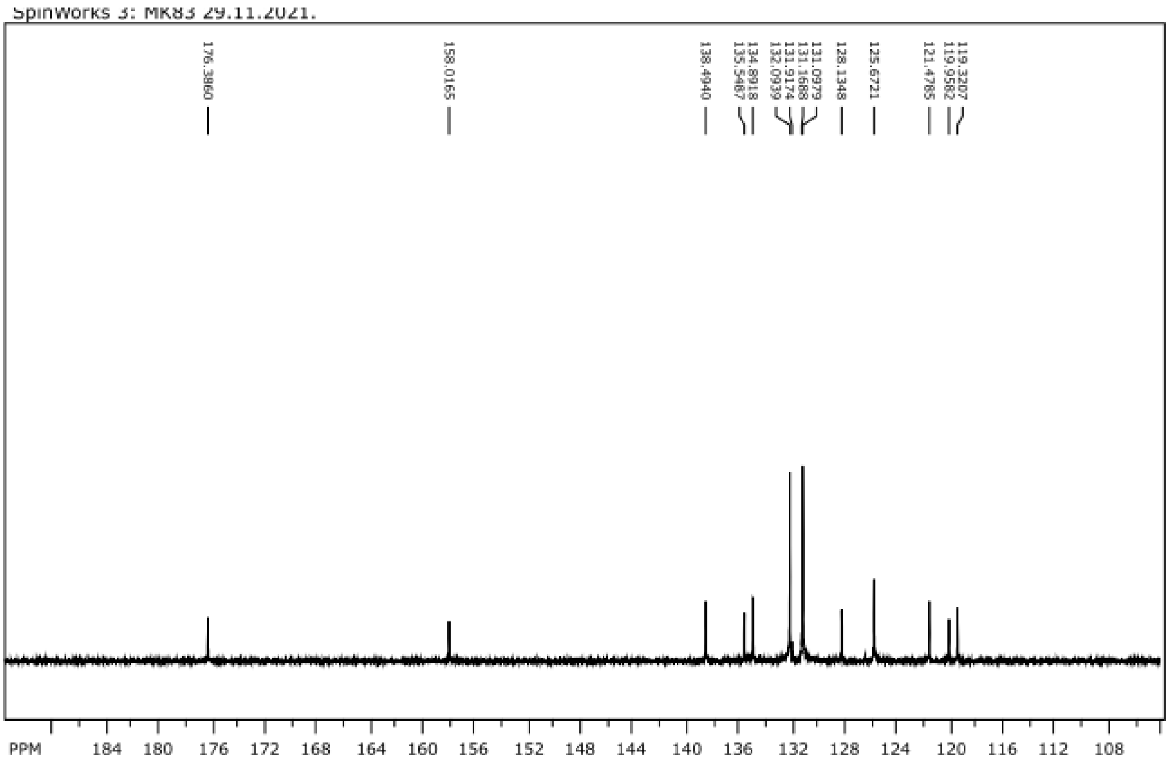

6,8-dichloro-2-mercapto-3-(3-methoxyphenyl)quinazolin-4(3H)-one (10j)

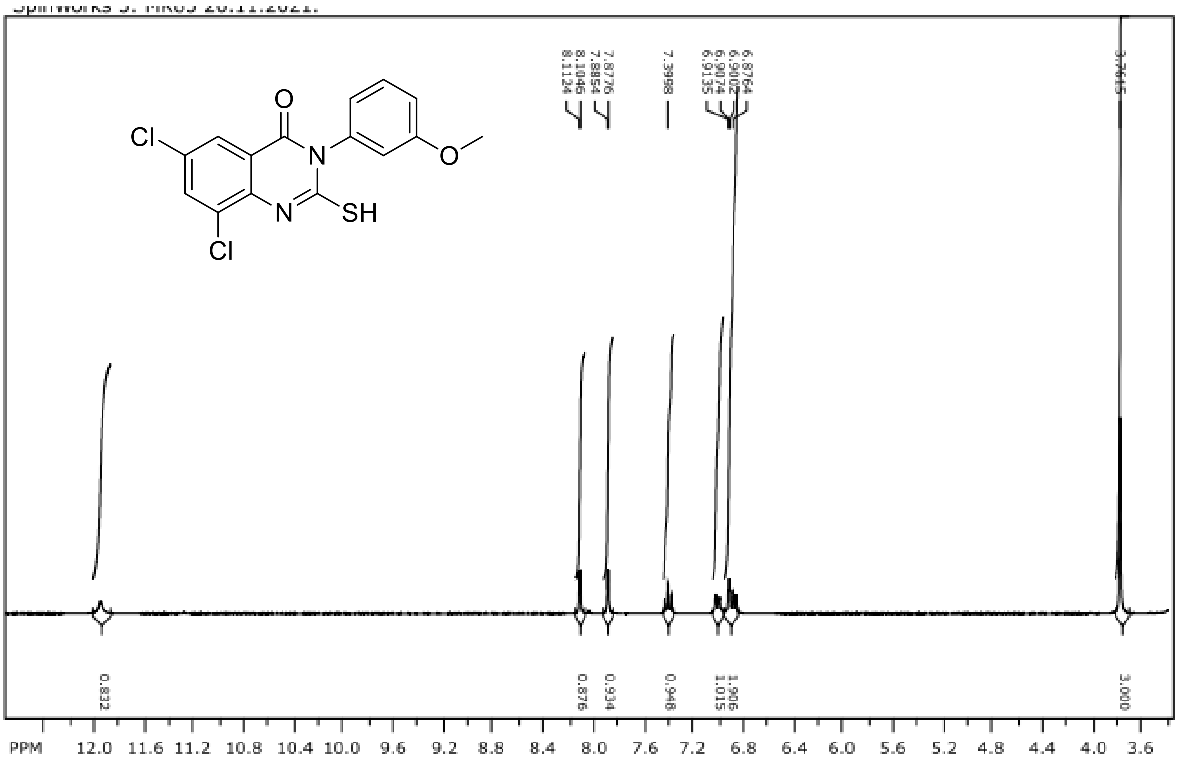

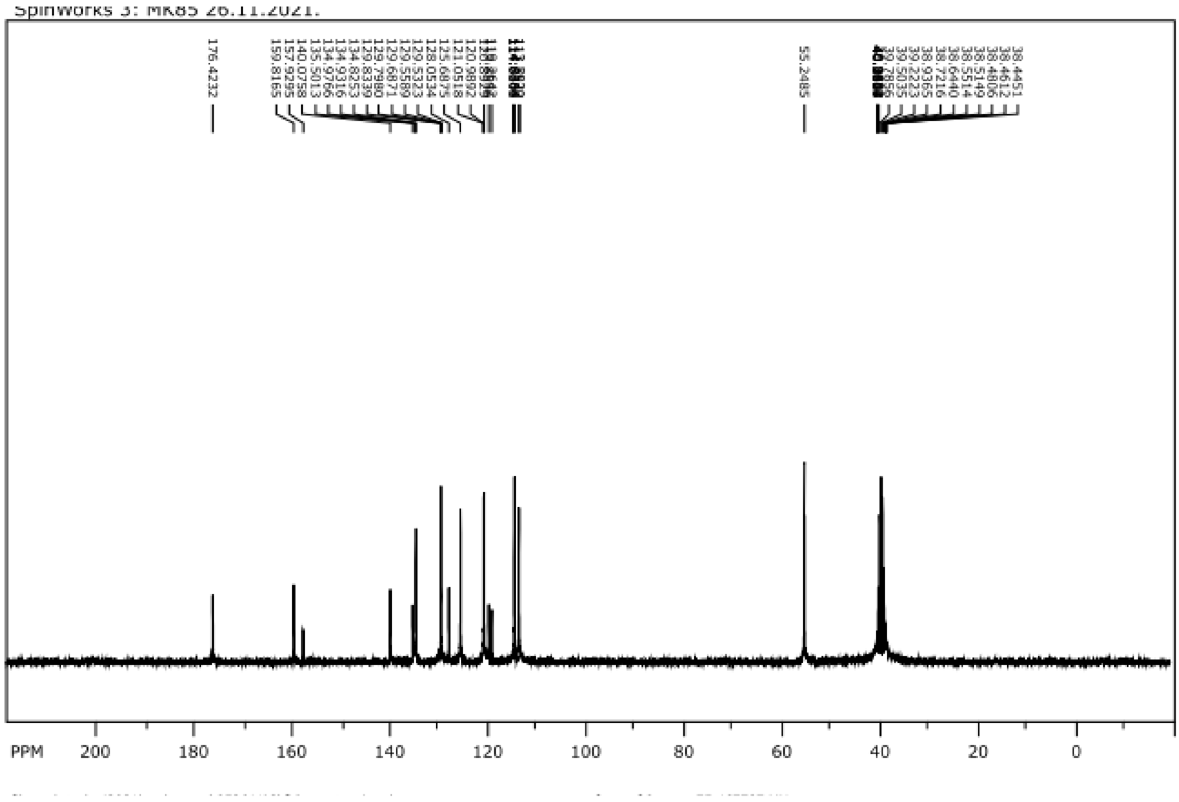

Supplement: Supplementary file 1 [file molecules-27-00558-s001.zip › molecules-1540454-supplementary.pdf]
